# Clonal diploid and autopolyploid breeding strategies to harness heterosis: insights from stochastic simulation

**DOI:** 10.1007/s00122-023-04377-z

**Published:** 2023-06-08

**Authors:** Marlee R. Labroo, Jeffrey B. Endelman, Dorcus C. Gemenet, Christian R. Werner, Robert Chris Gaynor, Giovanny E. Covarrubias-Pazaran

**Affiliations:** 1 Excellence in Breeding Platform, Consultative Group of International Agricultural Research, Texcoco, Mexico; 2 International Maize and Wheat Improvement Center (CIMMYT), Texcoco, Mexico; 3 Department of Horticulture, University of Wisconsin-Madison, Madison, WI 53706 USA; 4 Bayer Crop Science, 700 Chesterfield Pkwy W., Chesterfield, MO 63017 USA

## Abstract

**Key message:**

Reciprocal recurrent selection sometimes increases genetic gain per unit cost in clonal diploids with heterosis due to dominance, but it typically does not benefit autopolyploids.

**Abstract:**

Breeding can change the dominance as well as additive genetic value of populations, thus utilizing heterosis. A common hybrid breeding strategy is reciprocal recurrent selection (RRS), in which parents of hybrids are typically recycled within pools based on general combining ability. However, the relative performances of RRS and other breeding strategies have not been thoroughly compared. RRS can have relatively increased costs and longer cycle lengths, but these are sometimes outweighed by its ability to harness heterosis due to dominance. Here, we used stochastic simulation to compare genetic gain per unit cost of RRS, terminal crossing, recurrent selection on breeding value, and recurrent selection on cross performance considering different amounts of population heterosis due to dominance, relative cycle lengths, time horizons, estimation methods, selection intensities, and ploidy levels. In diploids with phenotypic selection at high intensity, whether RRS was the optimal breeding strategy depended on the initial population heterosis. However, in diploids with rapid-cycling genomic selection at high intensity, RRS was the optimal breeding strategy after 50 years over almost all amounts of initial population heterosis under the study assumptions. Diploid RRS required more population heterosis to outperform other strategies as its relative cycle length increased and as selection intensity and time horizon decreased. The optimal strategy depended on selection intensity, a proxy for inbreeding rate. Use of diploid fully inbred parents vs. outbred parents with RRS typically did not affect genetic gain. In autopolyploids, RRS typically did not outperform one-pool strategies regardless of the initial population heterosis.

**Supplementary Information:**

The online version contains supplementary material available at 10.1007/s00122-023-04377-z.

## Introduction

Breeding strategies can change the dominance as well as additive genetic value of populations over breeding cycles. Even though hybrid breeding strategies such as reciprocal recurrent selection (RRS) are widely used in diploid inbred-hybrid crops, such as maize (*Zea mays* L.), it is unknown when they increase the rate of genetic gain compared to other strategies because of trade-offs between their typically increased cycle length and costs vs. their increased use of dominance heterosis (Duvick 2005; Longin et al. 2012). Breeding programs considering changing to hybrid strategies to increase genetic gain per unit cost need additional research to inform their decisions. This is especially true for clonal crops, which show inbreeding depression and heterosis in some economically important traits but have unique features that may decrease the competitiveness of hybrid breeding strategies such as autopolyploidy, delayed flowering, and low multiplication rates (Aighewi et al. 2015; Diaz et al. 2021; Ceballos et al. 2015, 2020; Darkwa et al. 2020; Batte et al 2020; Lindhout et al. 2018; Werner et al. 2023; Amadeu et al. 2020).

The key reason to pursue a hybrid breeding strategy is to utilize heterosis and avoid inbreeding depression due to dominance while also increasing additive value. Fundamentally, dominance value refers to deviation of heterozygote genetic value from mean homozygote value at a locus (Falconer and Mackay 1996). For evolutionary and/or biochemical reasons, dominance may tend to be directional, i.e., positive in the direction of fitness (Lynch and Walsh 1998; Manna et al. 2011; Yang et al. 2017). The biologically dominant gene action of individual alleles of quantitative traits leads to population-wide heterosis and inbreeding depression (Hallauer et al. 2010; Lamkey and Edwards 1999; Labroo et al. 2021). Falconer and Mackay (1996) define inbreeding depression as the difference in mean value between a population at Hardy–Weinberg equilibrium (HWE) and the population if fully inbred to homozygosity; this is the definition used here. Heterosis is then sometimes considered the opposite of inbreeding depression (Falconer and Mackay 1996). However, heterosis can further include the difference in means between a population that exceeds heterozygosity of HWE and the population at HWE, a value of exceptional relevance to hybrid breeding. Lamkey and Edwards (1999) partition heterosis into values relevant to RRS programs. Panmictic heterosis is the difference in the inter-pool hybrid value to the mean of the intra-pool genotypes at HWE. Baseline heterosis refers to the difference in value of the intra-pool genotypes at HWE to the value of the intra-pool genotypes if fully inbred to homozygosity. It is a common misconception that the purpose of RRS is to obtain baseline heterosis, but in fact baseline heterosis can be utilized even in a single pool, and RRS specifically makes use of panmictic heterosis. Heterosis due to epistasis is possible and is not always the reversal of inbreeding depression; we do not discuss epistasis here (Lynch 1991; Lynch and Walsh 1998).

To harness heterosis due to heterozygote dominance, breeding strategies must attend to the frequency of heterozygote genotypes as well as the frequency of favorable alleles. Fixing a favorable allele leads to higher mean genetic value than maintaining the heterozygous genotype if dominance is incomplete—so maximizing heterosis is suboptimal with incomplete dominance—but before the favorable allele is fixed, the heterozygote has much higher value than the deleterious recessive state (Rembe et al. 2019). With overdominance, maintaining the heterozygote leads to higher mean genetic value than fixing the favorable allele. Even in the absence of true overdominance, linkage disequilibrium of dominant alleles in breeding populations can lead to pseudooverdominant haplotypes, which can behave as overdominant loci if not broken over the breeding time horizon (Jones 1917; Werner et al. 2023).

Breeding strategies differ in their ability to increase the frequency of favorable alleles and heterozygous genotypes and therefore differ in their ability to utilize heterosis due to dominance, which can entail both avoiding inbreeding depression due to dominance and building additional heterosis over HWE. Selection on One-Pool Breeding Value with random mating concentrates favorable alleles in the next generation, but it is challenging to increase the frequency of heterozygotes above approximately 0.5 in diploids, because HWE in the current generation is nearly constantly approached due to random mating (Hallauer et al. 2010; Falconer and Mackay 1996; Weinberg 1909). Fixation of favorable alleles with incomplete dominance can take many breeding cycles and recombination events for quantitative traits. Therefore, two-pool RRS on general combining ability (GCA) can be a viable alternative strategy when traits have appreciable dominance (Comstock et al. 1949; Schnell 1961; Hallauer et al. 2010). This strategy leads to the formation of heterotic pools with diverging allele frequencies as selection on GCA not only increases the frequency of favorable alleles, but also drives and drifts apart the frequencies of alleles between pools, particularly those which exhibit dominance (Duvick et al. 2004; Rembe et al. 2019). Upon inter-pool crossing, this difference in allele frequency produces an excess of heterozygous genotypes in the *F*_1_ hybrids compared to the frequency of heterozygous genotypes in the parent pools (Lamkey and Edwards 1999). The excess of heterozygosity leads to population-wide panmictic heterosis as excess dominance value is expressed in the inter-pool hybrids over the intra-pool parents, and the hybrids can even surpass HWE dominance value (Schnell 1982; Wricke and Weber 2010).

Panmictic heterosis occurs regardless of whether the intra-pool genotypes are fully inbred. If the intra-pool genotypes are inbred, as in maize, upon inter-pool crossing both panmictic heterosis and baseline heterosis (i.e., inbred-midparent heterosis) are observed in their hybrids, as heterozygosity exceeds not only the diverged pools if they were outbred but also the fully inbred lines (Lamkey and Edwards 1999). Fully inbred lines are not necessary to harness panmictic heterosis, and inbreds’ loss of baseline heterosis (inbreeding depression) is tolerated in non-clonal species because only inbred lines can stably reproduce genotypes (Schnell 1961).

Although RRS can systematically utilize heterosis, it can increase cycle length and costs compared to one-pool recurrent selection on breeding value, so RRS only is thought to increase genetic gain per unit cost if population heterosis is adequate. Potentially increased costs are due to maintaining separate pools of germplasm and evaluating both intra- and inter-pool materials (Longin et al. 2014). Increased cycle length is due to the need for inter-pool crossing and hybrid phenotypes to calculate GCA (Longin et al. 2014). Clearly, the use of genomic selection (GS) to decrease cycle length can increase the competitiveness of RRS with other strategies (Kinghorn et al. 2010; Rembe et al. 2019). Two-pool reciprocal recurrent genomic selection on GCA can achieve cycle lengths equal to one-pool recurrent genomic selection on breeding value because parents can be recycled on estimates of their value using their genotypes and relatives’ genotypes and phenotypes in a genomic prediction model rather than estimates requiring the hybrid progeny phenotypes (Kinghorn et al. 2010; Powell et al. 2020).

Animal breeders have developed intermediate strategies to avoid the challenges of RRS while still making some use of heterosis such as terminal crossing, or Two-Pool Breeding Value (Leroy et al. 2016; Swan and Kinghorn 1992). Terminal crossing involves parent selection on breeding value within two pools, which are subsequently crossed to obtain panmictic heterosis in inter-pool products via drift. With PS, terminal crossing has a shorter cycle length than RRS because parents can be recycled on breeding value without waiting for their hybrid progeny phenotypes to calculate GCA. Terminal crossing can be logistically simpler than RRS because no testcrossing is needed. Terminal crossing exploits some panmictic heterosis because allele frequencies within pools come to diverge by drift, but it exploits less panmictic heterosis than RRS on GCA because it does not actively select for divergence between pools. Therefore, the relative genetic gain per unit cost of these strategies may depend on population heterosis due to dominance.

A recently developed strategy to address dominance heterosis is selection on cross performance, particularly genomic estimated cross performance (Werner et al. 2023; Wolfe et al. 2021). Cross performance selects pairs of individuals for crossing in a single pool by the expected mean value of their progeny rather than mating randomly after selection on breeding value. Non-random mating allows combinations of alleles within a locus (i.e., genotypes) to be “cut-and-paste” from parents into progeny, so more heterozygosity and thus more dominance value is maintained than with random mating. In the presence of dominance, genomic prediction of cross performance has been demonstrated to outperform selection on genomic estimated breeding value with random mating in a single pool in clonal diploids (Werner et al. 2023). It may be that the cost savings and partial utilization of heterosis of cross performance could affect its relative genetic gain per unit cost compared to two-pool RRS, but this has not been explored previously.

Finally, the long-term benefit and short-term cost of stricter control of inbreeding in breeding populations is well understood (Woolliams et al. 2015). We further contend that hybrid breeding strategies’ relative performance reacts to different degrees of inbreeding control, and hybrid breeding can partly be viewed as a method to manage inbreeding depression in a population, as demonstrated by Fernández et al. (2021). For example, with dominance, recurrent selection on One-Pool Breeding Value may decrease genetic gain at high intensity due to high inbreeding and therefore high inbreeding depression, so a lower intensity may be optimal even at a relatively short time horizon. In contrast, RRS may be able to achieve higher intensity at the same time horizon, as inbreeding and inbreeding depression are relieved in the hybrids by the use of two pools and selection on GCA. Inbreeding is caused by selection and drift over breeding cycles, which lead to overrepresentation of homozygous genotypes in breeding generations compared to the base population at HWE. Populations at a given cycle may be approximately at HWE in terms of genotype frequencies, and thus not inbred per se, while still being inbred relative to the base population. Inbreeding due to concentration or fixation of favorable alleles, which can increase overall genetic value, is desirable. However, inbreeding due to drift can increase the frequency of unfavorable alleles and their homozygotes inadvertently. Inbreeding control prevents random loss of heterozygosity which decreases mean genetic value (i.e., causes inbreeding depression) in the presence of directional dominance. Of course, inbreeding control also limits drift of allele frequencies in favorable directions, which often leads to short-term costs. The optimal or acceptable inbreeding rate fundamentally depends on the time horizon of a breeding pipeline (Moeinizade et al. 2019). Different hybrid breeding strategies may have different relative performances at different time horizons as well as at different inbreeding rates, so inbreeding control may be practically necessary to reveal these differences.

Therefore, we compare selection on One-Pool Breeding Value, One-Pool Cross Performance, Two-Pool Breeding Value (terminal crossbreeding), and Two-Pool GCA (RRS) in model clonal crop breeding programs in terms of genetic gain per unit cost by stochastic simulation. We consider varied genetic architectures of heterosis due to dominance, ploidy levels, selection intensities (inbreeding rates), time horizons, estimation methods, and relative cycle lengths. These include scenarios with delayed flowering and slow multiplication and RRS scenarios that use fully inbred lines and/or combined intra- and inter-pool evaluation.

## Materials and methods

**Fig. 1:**
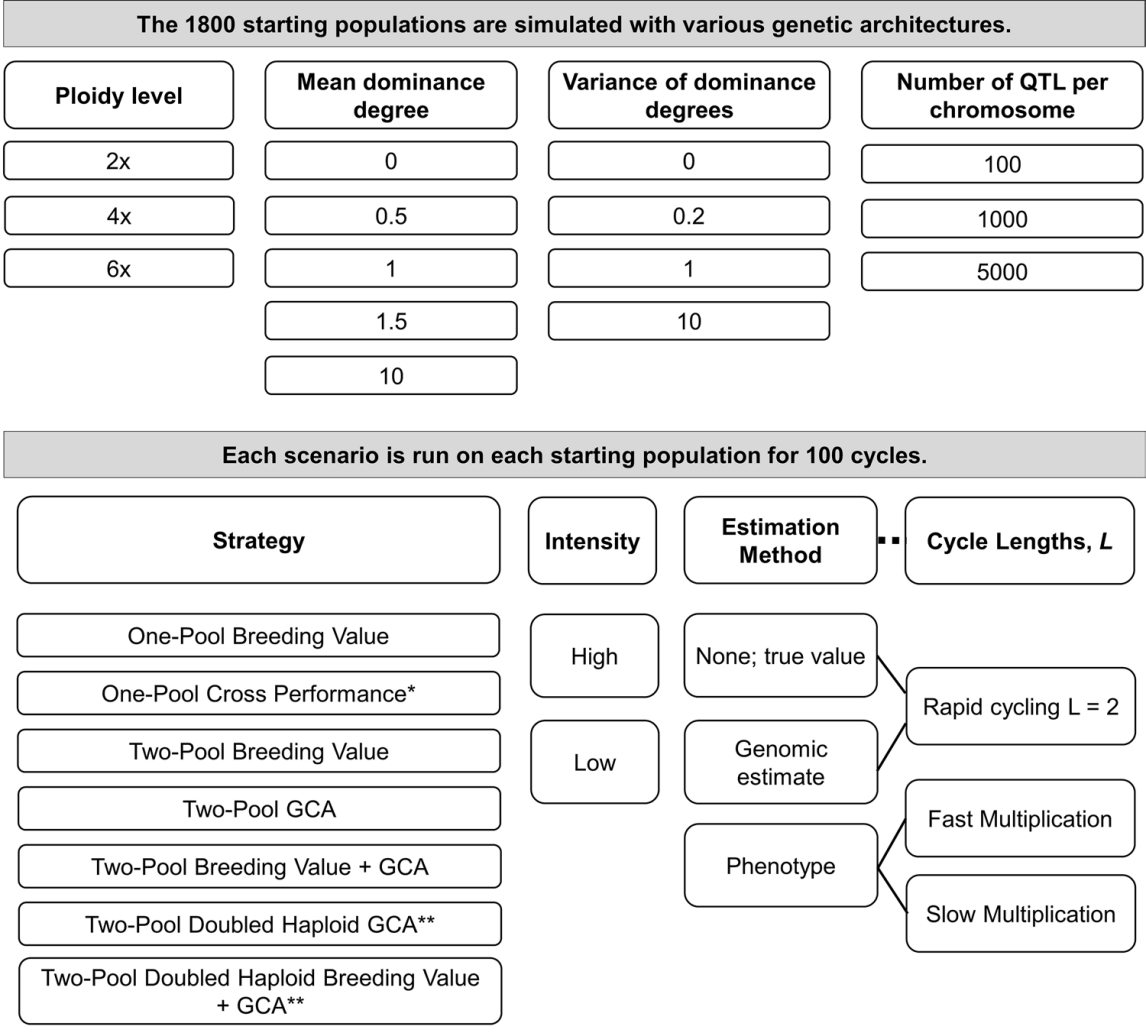
Overview of the study methods. All combinations of the scenario factors were assessed, except that the cycle lengths depended on the estimation method (solid lines) and a phenotypic estimate of One-Pool Cross Performance was not considered (*). Strategies with doubled haploids were only run for ploidy = 2 (**). Because multiple cohorts per cycle were not simulated, cycle length was varied by multiplying cycle number by the appropriate value and not by running an independent simulation (dashed line). Cycle lengths (*L*) by strategy and estimation method are given in Table 1

Stochastic simulations were conducted in the R 4.0.4 or 4.0.5 computing environment with the package AlphaSimR 1.0.1 on the International Maize and Wheat Improvement Center High-Performance Computing Cluster and the University of Wisconsin-Madison Center For High Throughput Computing Cluster (R Core Team 2021; Gaynor et al. 2021). The general procedure was that 180 different genetic architectures were simulated, with ten population replicates per architecture, then combinations of breeding strategies, selection intensities, and estimation methods were applied to each of the 1800 populations for 100 breeding cycles (Fig. 1). The responses were then measured with variously assumed cycle lengths.

### Trait genetic architecture simulation

The following steps were common to all scenarios. A genome with haploid *n* = 10 chromosomes was simulated using the Markovian Coalescent Simulator of Chen et al. (2009) within AlphaSimR. The “*GENERIC*” species history was used, which implied starting effective population size (*N*_*e*_) of 100 * ploidy/2, following the scaling recommendations of Arnold et al. (2012), and a mutation rate of 2.5 * 10^−8^ mutations per base pair. Following the genome simulation, a founder population of 80 non-inbred hermaphroditic individuals was drawn. We assumed no historic population split, which could affect the relative efficiency of the strategies (Lamkey and Edwards 1999).

Next, a trait with additive and dominance effects was simulated with a starting mean genetic value of zero and additive genetic variance of one. Dominance variance and thus total genetic variance varied depending on the subsequent dominance parameters. We did not consider epistatic, environment, or genotype x environment effects. To create trait genetic architectures for each scenario, all combinations of the following factors and their levels were simulated: number of quantitative trait loci (QTL) per chromosome of 100, 1000, or 5000; mean dominance degree (meanDD) of 0, 0.5, 1, 1.5, or 10; variance of the dominance degrees (varDD) of 0, 0.2, 1, or 10; and, ploidy of 2×, 4×, or 6× (Fig. 1).

As discussed more broadly in the AlphaSimR “Traits” vignette and Supplemental File 1, individuals’ true total genetic values were simulated as the sum of an intercept, total additive effect, and total dominance effect across all QTL controlling the trait, such that:

$$GV\left(x\right)= \mu +A\left(x\right)+D(x)$$

where *GV* is the individual total genetic value, *x* is vector of the individual’s QTL genotype dosages encoded as counts of the alternate allele from 0… $\phi$, *μ* is the intercept, *A*(*x*) is a vector of the summed additive QTL effects, and *D*(*x*) is a vector of the summed dominance QTL effects (Gaynor 2021). Please see Supplemental File 1 for the methods to simulate additive and dominance effects. Autopolyploid genetic values were assigned assuming digenic dominance interactions only (Gaynor 2021; Gallais 2003). Varying the number of QTL, mean dominance degree, variance of the dominance degrees, and ploidy with ten replicates per combination led to 1800 populations (3 * 5 * 4 * 3 * 10) with varied amounts of initial population heterosis (*H*_0_) as well as varied starting dominance and total genetic variance, all of which were recorded (Supplemental Fig. 1; Gaynor et al. 2018).

*H*_0_ was the difference in mean value of the initial population at HWE ($${P}_{{0}_{HWE}})$$ from the initial population if fully inbred to homozygosity ($${P}_{{0}_{I}})$$; it was divided by the initial genetic standard deviation ($${\sigma }_{{G}_{0}})$$ to allow comparison across populations with traits at different scales (Supplemental Fig. 1). As such, $${H}_{0}={(P}_{{0}_{HWE}}- {P}_{{0}_{I}}) / {\sigma }_{{G}_{0}}$$ (Falconer and Mackay 1996). With all else equal, the amount of *H*_0_ increases as the mean dominance degree and the square root of the number of QTL increase and decreases as the variance of the dominance degrees increases; however, the effect of the variance of the dominance degrees is relatively smaller (Supplemental Fig. 1; Gaynor et al. 2018). We did not control linkage disequilibrium, which also affects *H*_0_, so simulating populations with identical parameters as in this study may lead to slightly different *H*_0_ as their linkage disequilibrium varies (Gaynor et al. 2018). Occasional negative H_0_ was observed in architectures with meanDD = 0 and varDD > 0 due to random sampling of dominance degrees, which sometimes led to negative directional dominance in the starting population. Each single trait modeled can be interpreted as representing an index of quantitative traits.

### Breeding scenario and strategy description

Each simulation was initiated by drawing 40 individuals from a given founder population. For simulations with two pools, the 40 individuals were randomly split into two pools of 20 (Cowling et al. 2020). Then, a combination of strategy, selection intensity, and estimation method was applied for 100 cycles. Responses were subsequently interpreted with variously assumed cycle lengths. A scenario was defined as a combination of strategy, estimation method, selection intensity, and assumed relative cycle lengths (Fig. 1). Code for all scenarios is available at https://github.com/gaynorr/ClonalHybridStrategies.

**Fig. 2:**
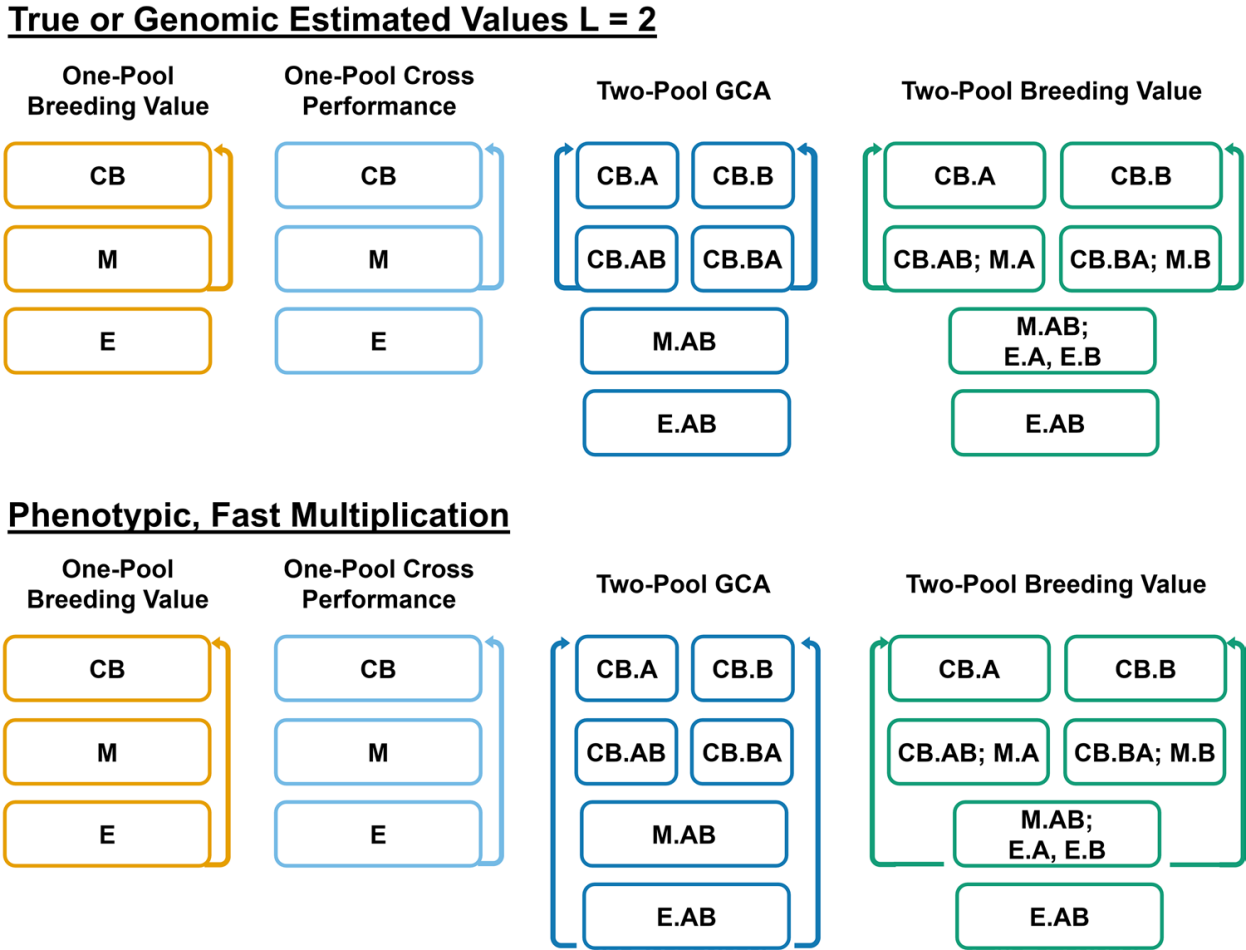
Graphical overview of the core breeding strategies with true, genomic estimated values, or phenotypic values and fast multiplication. Crossing blocks (CB), multiplication seasons (M), and evaluation seasons (E) are indicated. For the two-pool strategies, activities for intra-pool A (.A), intra-pool B (.B), and inter-pool hybrids AB (.AB) are indicated. Arrows indicate the recycling stage

A brief description of each strategy follows. For conciseness, the program sizes are represented by variables, and the values of variables for each scenario are given in Supplemental Table 1. Detailed scenario descriptions are given in Supplemental Table 2. A graphical overview for some scenarios is in Fig. 2. Parents were randomly mated in the first cycle, and in all subsequent cycles a crossing plan conferring maximum avoidance of inbreeding was used (Kimura and Crow 1963). To achieve maximum avoidance of inbreeding, two full siblings per family (cross) were always selected. These individuals were then mated to the pair of siblings least related to them in the population (Wright 1921). At low intensity, 200 parents per pool were selected for one-pool strategies, and 100 parents per pool were selected for two-pool strategies. At high intensity, 40 parents per pool were selected for one-pool strategies, and 20 parents per pool were selected for two-pool strategies.

- One-Pool Breeding Value: This is a specific type of recurrent selection. The *r* parents are made into *x* crosses with *y* progeny per cross, totaling *z* individuals. The *z* progeny are phenotyped. At the appropriate scenario timepoint, two individuals per family (cross) are selected using the scenario estimate of breeding value. The cycle restarts with the selected individuals.
- Two-Pool Breeding Value: This is synonymous with terminal crossing. Within each pool, the *r* parents are made into *x* crosses with *y* progeny per cross, totaling *z* intra-pool progeny per pool. The *z* intra-pool progeny are phenotyped. From each pool, two individuals are then selected randomly. For both pools, all *z* intra-pool progeny per pool are crossed to both individuals selected from the opposing pool, and each inter-pool cross produces one progeny, creating *w* inter-pool progeny. The inter-pool progeny are phenotyped. At the appropriate scenario timepoint, two individuals per family (cross) within each pool are selected on the scenario estimate of intra-pool breeding value. The cycle restarts with the selected individuals.
- One-Pool Cross Performance: This is a specific type of recurrent selection. The *r* parents are made into *x* crosses with *y* progeny per cross, totaling z individuals. The *z* progeny are evaluated. To conduct maximum avoidance of inbreeding with cross performance, the pairs of families (crosses) which satisfy a maximum avoidance of inbreeding plan are identified. Within those pairs of families, the value of all inter-family crosses of their individual members are calculated or estimated according to scenario. The two best crosses from each set of paired families are selected at the scenario appropriate timepoint. The cycle restarts with the selected crosses. Scenarios with a phenotypic estimation method were not considered; although phenotypic cross performance can be estimated as the mean of the parental phenotypes, this scenario was too computationally intensive with phenotypic program sizes used.
- Two-Pool GCA: This is a specific type of RRS. Within each pool, the *r* parents are made into *x* crosses with *y* progeny per cross, totaling z intra-pool progeny per pool. From each pool, two individuals are selected randomly. For both pools, all *z* intra-pool progeny per pool are crossed to both individuals selected from the opposing pool, and each inter-pool cross produces one progeny, creating *w* inter-pool progeny. The inter-pool progeny are phenotyped. At the scenario appropriate timepoint, two individuals per family (cross) are selected on the scenario estimate of GCA within each pool as parents. The cycle restarts with the selected individuals.
- Two-Pool Breeding Value + GCA: This is a specific type of RRS. These strategies have the same structure as Two-Pool GCA, except that the intra-pool progeny are evaluated before testcrossing. The top ~ 75% of individuals per family (cross) are advanced on the scenario estimate of breeding value according to scenario, and only the advanced individuals are used in testcrossing.

**Table 1:** Sets of cycle lengths (*L*) in seasons assumed to be required for each strategy in a given scenario

	True values *L* = 2	Genomic estimated values *L* = 2	Phenotypic values, fast multiplication	Phenotypic values, slow multiplication
One-pool breeding value	2	2	3	4
One-pool cross performance	2	2		
Two-pool breeding value	2	2	3	4
Two-pool GCA	2	2	4	7
Two-pool doubled haploid GCA	2	2	5	8
Two-pool breeding value + GCA	2	2	4	7
Two-pool doubled haploid breeding value + GCA	2	2	5	8

For ploidy = 2 only, we considered two additional selection strategies to address proposed inbred-hybrid clonal crops:

- Two-Pool Doubled Haploid GCA: This is a specific type of RRS. These strategies have the same structure as Two-Pool GCA, except that all intra-pool progeny were used to create a single doubled haploid line per progeny in the season before testcrossing.
- Two-Pool Doubled Haploid Breeding Value + GCA: This is a specific type of RRS. These strategies had the same structure as Two-Pool GCA, except that all intra-pool progeny were used to create a single doubled haploid line per progeny in the season following intra-pool crossing. The intra-pool doubled haploid lines were evaluated before testcrossing and the top ~ 75% of individuals per family (cross) were advanced on the scenario estimate of breeding value, and only these advanced individuals were used in testcrossing.

### True, genomic estimated, and phenotypic values

Details of the calculation of true and estimated breeding value, expected mean cross performance, and GCA are provided in Supplemental File 1.

Genomic prediction models used a genotypic, rather than breeding value, parameterization (Vitezica et al. 2013). The 2000 most recently evaluated inter-pool individuals comprised the training set for the two-pool programs, and the 2000 most recently evaluated intra-pool individuals comprised the training set for the one-pool programs (Supplemental Table 1). We did not explore use of intra-pool information to predict intra-pool breeding value with the Two-Pool Breeding Value strategy, nor to predict intra-pool breeding value in the Two-Pool Breeding Value + GCA and Two-Pool Doubled Haploid Breeding Value + GCA scenarios.

For scenarios which used genomic estimates, either of two models were used. The first was best linear unbiased prediction with directional dominance (RRBLUP_D), as described by Xiang et al. (2016). The model is $${\varvec{y}}=\mathbf{X}\upbeta +\mathbf{f}b+ \mathbf{Z}\mathbf{a}+\mathbf{W}{\mathbf{d}}^{\boldsymbol{*}}+$$** e**. The response ***y*** is the vector of phenotypic values; $$\upbeta$$ are the fixed effects of the intercept with the associated design matrix **X**; $$b$$ are the fixed effects of inbreeding depression with the associated marker inbreeding coefficients **f**; $$\mathbf{a}$$ are the random additive effects with the associated design matrix **Z**; $${\mathbf{d}}^{\boldsymbol{*}}$$ are the random dominance effects with the associated design matrix **W**; and, $$\mathbf{e}$$ is random error assumed to be normally, identically, and independently distributed. **Z** is a matrix of *i* individuals in rows, *j* SNPs in columns, and the respective scaled additive genotype dosages ($${x}_{{A}_{ij}})$$ per cell such that $${x}_{{A}_{ij}}=({x}_{ij}- \frac{\upphi }{2})(\frac{2}{\upphi }$$), where $${x}_{ij}$$ is the raw genotype dosage of the individual SNP locus *ij* and $$\upphi$$ is the ploidy level (Gaynor 2021). **W** is an identical matrix except it contains scaled dominance genotype dosages ($${x}_{{D}_{ij}})$$ per cell such that $${x}_{{D}_{ij}}={x}_{ij}(\upphi -{x}_{ij})({\frac{2}{\upphi })}^{2}$$. For each individual *i* in the vector **f**, $${f}_{i}$$ is $$1- \frac{\mathbf{W}1}{N}$$, where *N* is the total number of markers.

The second model was best linear unbiased prediction of GCA (RRBLUP_GCA) as described by Eq. 3 of Technow et al. (2012). The model is $${\varvec{y}}=\mathbf{X}\upbeta + {\mathbf{Z}}_{{\varvec{M}}}{\mathbf{a}}_{{\varvec{M}}}+{\mathbf{Z}}_{{\varvec{P}}}{\mathbf{a}}_{{\varvec{P}}}+\mathbf{e}$$**.** The terms $${\varvec{y}}$$**,** $$\mathbf{X}\upbeta$$, and $$\mathbf{e}$$ are as in RRBLUP_D; ***y*** contains *F*_1_ hybrid phenotypes. $${\mathbf{Z}}_{{\varvec{M}}}{\mathbf{a}}_{{\varvec{M}}}$$ and $${\mathbf{Z}}_{{\varvec{P}}}{\mathbf{a}}_{{\varvec{P}}}$$ are the additive effects of each of the two heterotic pools, respectively. $${\mathbf{Z}}_{{\varvec{M}}}$$ is a matrix of maternal parents in rows, SNPs in columns, and the respective scaled maternal genotypes ($${x}_{{M}_{{A}_{ij}}})$$ per cell such that $${x}_{{M}_{{A}_{ij}}}=({x}_{{M}_{ij}}- \frac{\upphi }{4})(\frac{4}{\upphi }$$); this accounts for the ½ contribution of the maternal genotype to the hybrid phenotype. $${\mathbf{Z}}_{{\varvec{P}}}$$ is as $${\mathbf{Z}}_{{\varvec{M}}}$$, except it contains the paternal parents in rows and scaled paternal genotypes ($${x}_{{P}_{{A}_{ij}}})$$ in cells.

Phenotypes in the study referred to single phenotypic values per entry with a fixed error variance and an initial broad-sense heritability of 0.5, which represent replicated phenotypes. In subsequent cycles, heritability changed as true genetic variance changed, because we assumed a fixed phenotyping effort over cycles. As such, the phenotypic value (*P*) of an individual is calculated as:

$$P=GV+ \varepsilon$$

where *GV* is the true genetic value of the individual as described above, and $\varepsilon$ is random error drawn from a normal distribution with a mean of zero, and variance ${\sigma }_{\varepsilon }^{2}$ such that $0.5= \frac{{\sigma }_{{G}_{0}}^{2}}{{\sigma }_{{G}_{0}}^{2}+ {\sigma }_{\varepsilon }^{2}}$, where ${\sigma }_{{G}_{0}}^{2}$ is the initial total genetic variance of the population.

### Costing and genotyping assumptions

Genotypic information was obtained from a simulated SNP-chip with 100 markers per chromosome; the number of markers was not varied. To control resources across strategies, we varied program size by decreasing the number of progeny per cross first, then decreasing the number of crosses if necessary (Supplemental Table 1). We assumed that the costs of making crosses, multiplying materials, and growing out non-evaluated plots were negligible. The cost of evaluation plots was assumed to be equal across strategies. For further comparisons, we defined all costs in terms of evaluation plots. We assumed that the cost of generating a doubled haploid line was three times the cost of an evaluation plot. We assumed that the cost of phenotyping an individual was equal to the cost of genotyping an individual. With use of outbred intra-pool parents, genotyping both intra-pool parents and their inter-pool segregating progeny was necessary to predict GCA. In the doubled haploid scenarios, we assumed that both intra- and inter-pool genotypes were genotyped, even though the inter-pool progeny genotypes could be inferred from their doubled haploid parents under the assumed cycle lengths. Scenarios which used true values were identical in size to scenarios with genomic estimated values; cost is not a realistic consideration to obtain true values, and the true value scenarios were used to consider a situation with perfect accuracy.

### Strategy cycle lengths

Strategy cycle length was assumed to depend on the estimation method (Table 1, Supplemental Table 2). Strategies which used true values or genomic estimates were assumed to have a cycle length of two seasons, which was considered a realistic rapid-cycling length. Some rapid-cycling GS programs may achieve a one-season cycle length, but this is uncommon due to practical constraints (Gaynor et al. 2017). Phenotypic strategies were considered to have different cycle lengths depending on whether fast or slow multiplication was possible. Scenarios with slow multiplication were also assumed to have slow flowering, as occurs in white yam (*Dioscorea rotundata*; A. Amele, pers. comm.). Fast multiplication indicated that adequate material for the next crossing block was available in the season following crossing, but adequate material for phenotypic evaluation was available in the second season following crossing. Slow multiplication implied adequate material for crossing and evaluation was available after two seasons following crossing. Doubled haploid production was assumed to require one season. We assumed that a single cohort and breeding stage occurred per season, although typical programs may run multiple cohorts at different stages in parallel per season (Covarrubias-Pazaran et al. 2021). As such, to modify the cycle length, the cycle numbers for a given strategy, estimation method, and intensity were multiplied by the appropriate value. For example, the PS scenarios with fast and slow multiplication were obtained from the same simulations, and fast and slow multiplication cycle lengths were imposed by multiplying the cycle number by the strategy cycle length. We assumed that both phenotypic and genotypic information became available post-flowering. All cycle lengths under all assumed constraints are reported in Table 1.

### Reported responses

Responses were reported for all scenarios after 15 and 50 years of breeding, at which timepoints genetic variance was nonzero for all scenarios. Responses were also reported for PS at the same cycle numbers (8 and 25) as GS scenarios to demonstrate the effect of using GS as an estimation method, without using it to reduce cycle length, on the relative performance of PS and GS. The reported responses follow.

- For one-pool scenarios, genetic gain ($${\Delta }_{G}$$) was the mean genetic value at a given timepoint in the intra-pool genotypes ($${G}_{t})$$ minus the mean genetic value of the founder population ($${G}_{0})$$ and divided by the starting population genetic standard deviation ($${\sigma }_{{G}_{0}})$$, or $${\Delta }_{G} =$$ ($${G}_{t}- {G}_{0}) / {\sigma }_{{G}_{0}}$$. For the two-pool scenarios, the method was the same except the inter-pool genotypes were used. This allowed comparison of genetic gain in the product pools of both scenarios. Genetic gain was also reported for the intra-pool genotypes in the Two-Pool GCA, Two-Pool Doubled Haploid GCA, Two-Pool Breeding Value + GCA, and Two-Pool Doubled Haploid Breeding Value + GCA scenarios.
- Mean additive value and mean dominance value were reported at a given cycle in the respective product pools for one-pool and two-pool scenarios and divided by the starting population genetic standard deviation.
- Population inbreeding depression (${P}_{ID}$) was reported for the product pools of the scenarios as the difference in mean value between the population at HWE (${P}_{HWE}$) and the population if fully inbred to homozygosity (${P}_{I}$), then divided by the starting population standard deviation ${\sigma }_{{G}_{0}}$, or ${{P}_{ID}=(P}_{I}- {P}_{HWE}) / {\sigma }_{{G}_{0}}$ (Falconer and Mackay 1996).
- The genomic inbreeding coefficient *f* was reported for the product pools relative to their initial populations based on a genomic (**G**) additive relationship matrix with allele frequencies from the initial population (VanRaden 2008; Method 1). For diploids, the mean diagonal of **G** equals 1 + *f* (Powell et al. 2010; Endelman and Jannink 2012). The more general relationship for ploidy *ϕ* is that the mean diagonal of **G** equals 1 + (*ϕ* − 1)*f* (Gallais 2003). The inbreeding coefficient was used only to compare inbreeding for identical strategies at high vs. low selection intensity.
- Panmictic heterosis $PH$ was reported for the two-pool strategies as the difference in the inter-pool hybrid value (${P}_{{F}_{1}}$) to the mean of the intra-pool genotypes at HWE (${P}_{{A}_{HWE}}$, ${P}_{{B}_{HWE}}$) and divided by ${\sigma }_{{G}_{0}}$, or ${PH=(P}_{{F}_{1}}- \frac{1}{2}({P}_{{A}_{HWE}}+ {P}_{{B}_{HWE}})) / {\sigma }_{{G}_{0}}$ (Lamkey and Edwards 1999).

Additional responses are available in the raw data at https://github.com/gaynorr/ClonalHybridStrategies. We wish to highlight that the methods used do not permit meaningful comparisons of absolute or scaled response values across ploidies. For example, observing that a breeding program for autohexaploids has greater mean genetic value than a diploid at a given cycle does not necessarily imply that more gain is possible in autohexaploids.

### Regression analysis of responses

For clarity, results were grouped by the question of interest. The core strategies to explore the optimal breeding strategy across *H*_0_ were One-Pool Breeding Value, One-Pool Cross Performance, Two-Pool Breeding Value, and Two-Pool GCA. The core strategies were also used to explore the optimal estimation method—i.e., genomic estimated or phenotypic—under the experimental assumptions. The non-core strategies, Two-Pool GCA, Two-Pool Breeding Value + GCA, Two-Pool Doubled Haploid GCA, and Two-Pool Doubled Haploid Breeding Value + GCA, were used to assess whether combined selection on intra-pool breeding value and inter-pool GCA increased gain with or without fully inbred intra-pool parents. The non-core strategies were also used to assess whether use of fully inbred diploid intra-pool parents increased the rate of genetic gain.

To analyze and plot the results, each response at the timepoint of interest (15 years, 50 years, or 25 cycles) for the questions of interest (core or non-core strategies) was linearly modeled in base R as follows:

$${Y}_{ijk}= \mu +{S}_{i}+{H}_{j}+{SH}_{ij}+ {\varepsilon }_{ijk}$$

where ${Y}_{ijk}$ was the response value for the fixed *i*th scenario *S*, the fixed *j*th *H*_0_ value *H*, their fixed *ij*th interaction *SH*, and the random *ijk*th error $\varepsilon$ of the simulation replicate. The scenario of a response was the combination of strategy, estimation method, selection intensity, and assumed cycle length. All effects were assumed to be normally and independently distributed. The coefficient of determination (*R*^2^) value, slope, slope standard error, intercept, and intercept standard error was recorded for each regression (Supplemental File 2). The regressions, the 95% confidence interval of their predicted means, and, at times, raw data points were plotted using the R package ggplot2 (Wickham 2011). The intersections of the regressions which occurred within the surveyed *H*_0_ values and, when possible, their standard errors were also estimated (Supplemental File 3). The standard errors of the intersections were estimated by maximum likelihood with the R package nlme and used to calculate the 95% confidence interval of the intersection; the standard errors were typically inestimable when the regressions had similar values over a large range (whuber 2020; Pinheiro et al. 2017). In accordance with recent guidelines of the statistical community, significance testing was not conducted and confidence intervals were interpreted (Wasserstein and Lazar 2016; Alexander and Davis 2022).

## Results

### Genetic gain in the core strategies

The relative performance of the core strategies depended on *H*_0_, the time horizon, the selection intensity in the program, the relative cycle lengths among strategies, the estimation method, ploidy level, and their interactions. Typically, the comparative advantage of Two-Pool GCA increased with increased *H*_0_, time horizon, and selection intensity, as well as with use of GS, but it decreased with increased ploidy level or increased cycle length.

**Fig. 3:**
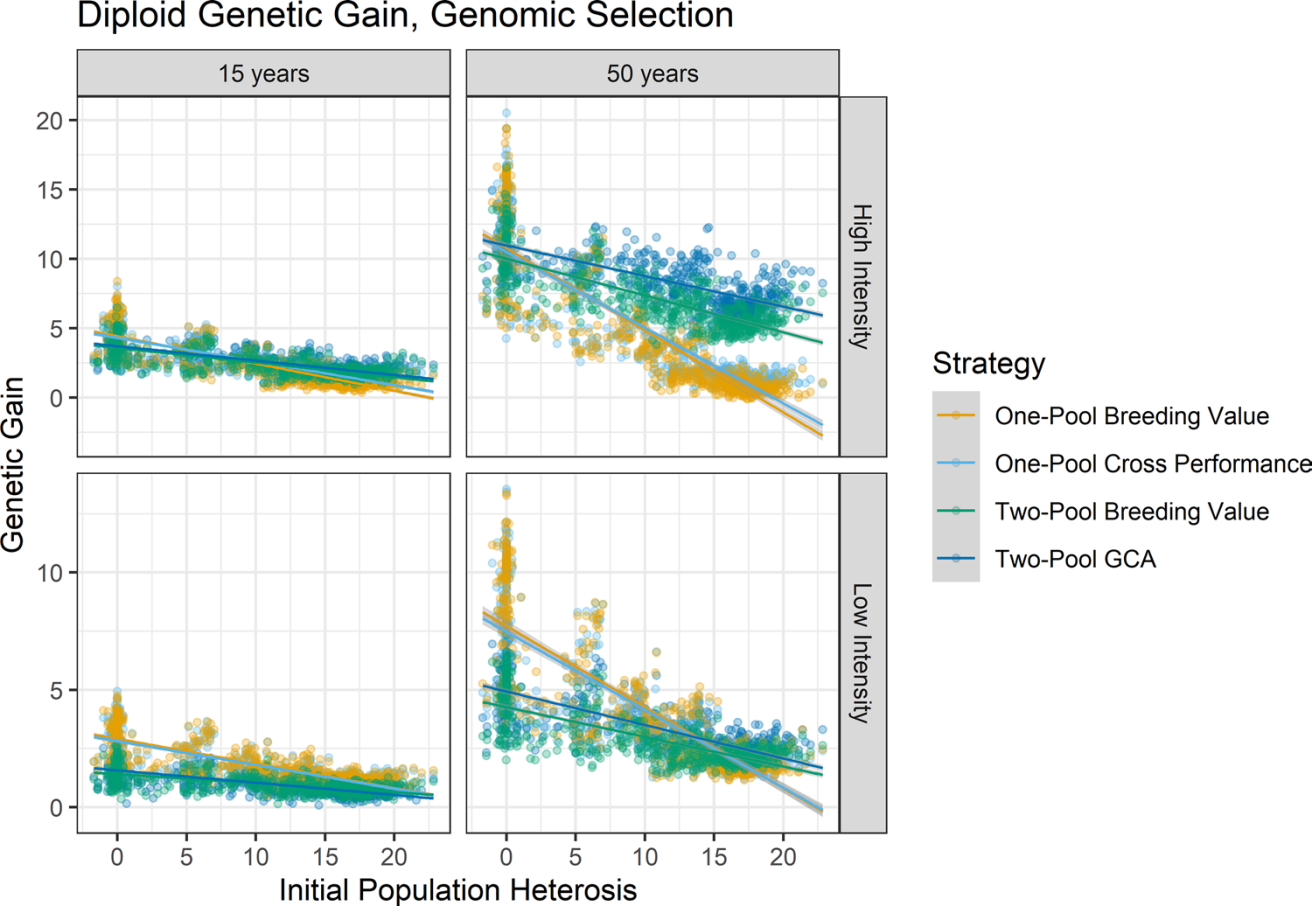
Genetic gain in diploids after 15 and 50 years with use of GS regressed on breeding scenario, initial population heterosis, *H*_0_, and their interaction. Colored lines indicate regressions by breeding strategy with GS and cycle length 2, and grey bands indicate the standard error of the predicted means. Dots indicate raw data points and dot color indicates strategy as in the lines

With use of GS in the clonal diploids, at high-intensity Two-Pool GCA was the best strategy after 15 years if *H*_0_ was greater than 9.0, and One-Pool Breeding Value or One-Pool Cross Performance was the best strategy if *H*_0_ was lower (Fig. 3; Supplemental File 3). After 50 years, Two-Pool GCA was the best predicted strategy at all positive *H*_0_ values, and its relative advantage increased as *H*_0_ increased (Fig. 3). In contrast, at low intensity, one-pool strategies were always better than Two-Pool GCA after 15 years (Fig. 3). After 50 years, at low intensity Two-Pool GCA only outperformed One-Pool Breeding Value if *H*_0_ was greater than 13.7, a substantially greater amount of *H*_0_ than at high intensity (Fig. 3; Supplemental File 3). High-intensity programs had greater genetic gain than low-intensity programs on average, but low-intensity one-pool strategies outperformed high-intensity one-pool strategies if *H*_0_ was relatively high (Fig. 3). (Of course, high-intensity two-pool strategies still outperformed the best low-intensity one-pool strategy over the range at which low-intensity one-pool strategies outperformed high-intensity one-pool strategies.)

With use of PS and fast multiplication in clonal diploids, Two-Pool GCA was not the best strategy after 15 years at any *H*_0_ value (Supplemental Fig. 2). After 50 years, it required *H*_0_ greater than 13.9 to outperform other strategies, and the amount of overperformance was relatively less than with GS (Supplemental Fig. 3; Supplemental File 3). With PS and slow multiplication, Two-Pool GCA was never the best strategy over the time horizons surveyed, and One-Pool Breeding Value or Two-Pool Breeding Value were higher performing (Supplemental Figs. 2, 3).

**Fig. 4:**
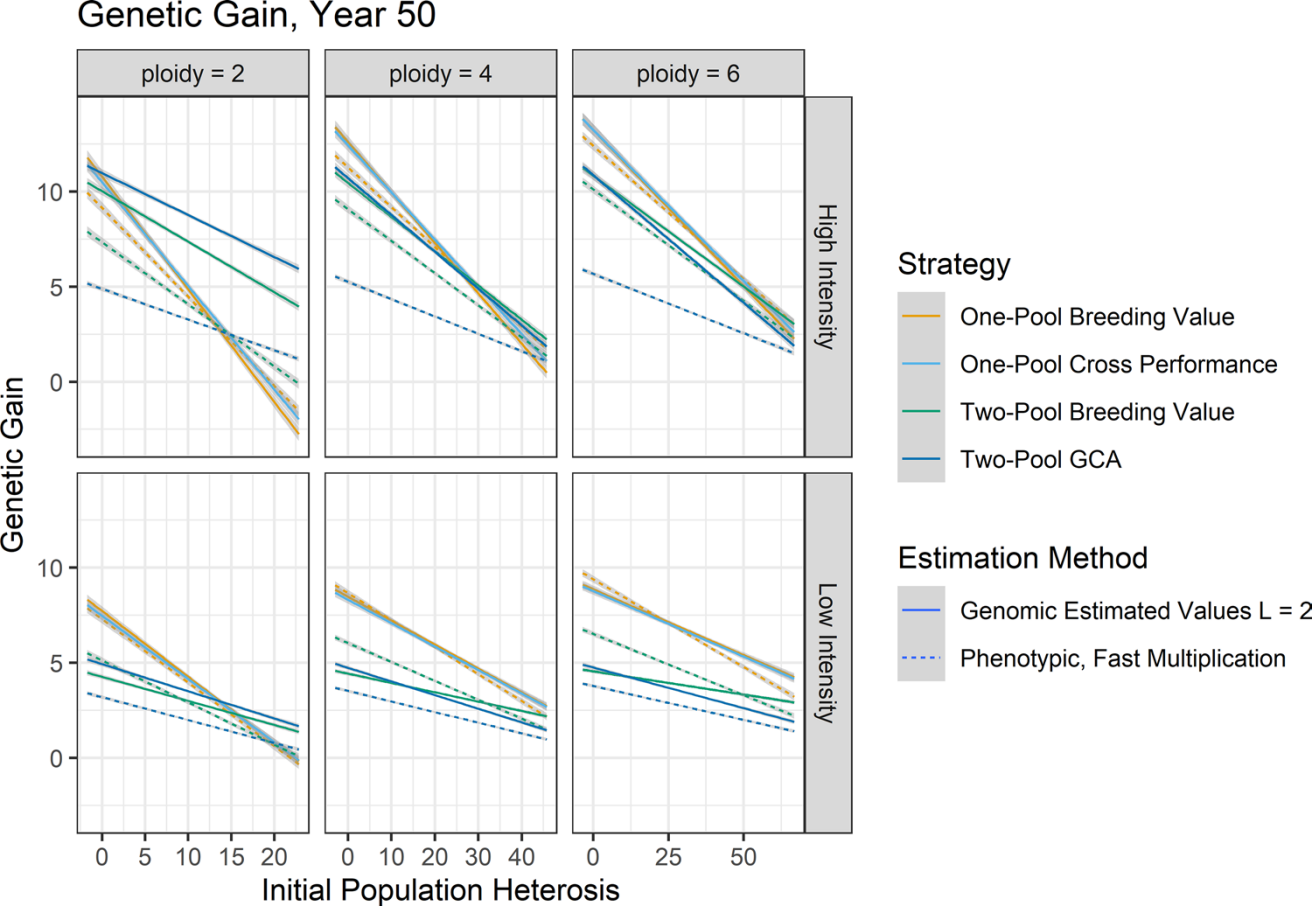
Genetic gain for each ploidy level after 50 years of breeding with use of genomic and phenotypic selection and various strategies as a function of *H*_0_, breeding scenario, and their interaction. Line color indicates strategy, and grey bands indicated the standard error of the predicted mean. Line type indicates estimation method with the accompanying set of cycle lengths (*L*)

With use of GS in the clonal autopolyploids, Two-Pool GCA showed fewer advantages than in diploids, and One-Pool Breeding Value or One-Pool Cross Performance were typically better strategies (Fig. 4). At high intensity after 15 years, One-Pool Breeding Value or One-Pool Cross Performance were the best strategies for both autotetraploids and autohexaploids (Supplemental Figs. 4, 5). One-Pool Cross Performance was the better strategy at high *H*_0_, and One-Pool Breeding Value was the better strategy at low *H*_0_. After 50 years at high intensity in the autotetraploids, One-Pool Breeding Value or One-Pool Cross Performance provided the most gain if *H*_0_ was less than or equal to 31.7 ± 2.3; if *H*_0_ was greater, Two-Pool GCA or Two-Pool Breeding Value provided the most gain, but the advantages were small (Fig. 4; Supplemental File 3). In the autohexaploids, the same strategy pattern was apparent but the intersection occurred at *H*_0_ of 56.8 ± 4.0 (Fig. 4; Supplemental File 3). At low selection intensity, One-Pool Breeding Value or One-Pool Cross Performance provided the most gain at both timepoints for both autotetraploids and autohexaploids (Fig. 4).

**Fig. 5:**
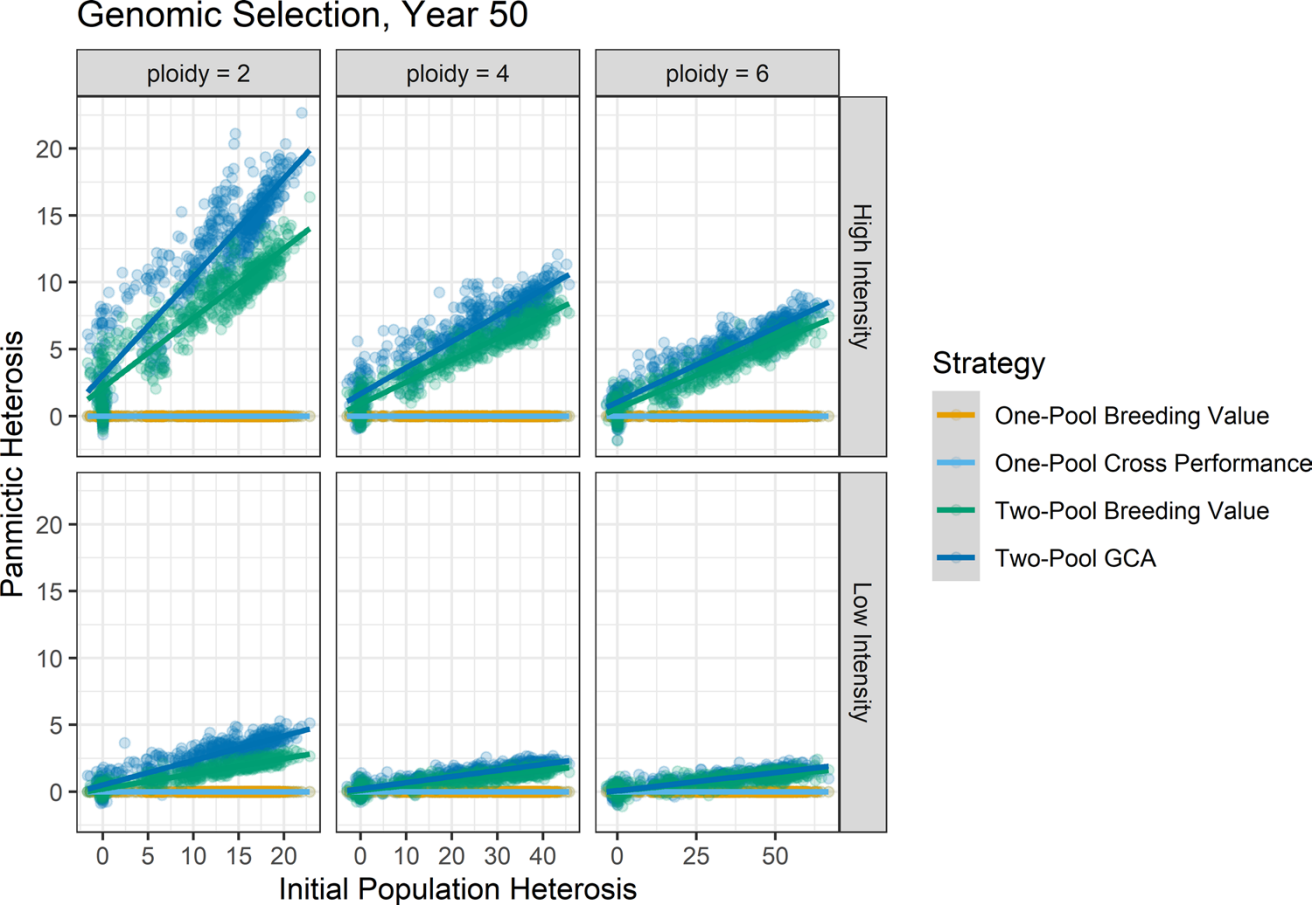
Panmictic heterosis for each ploidy as a function of initial population heterosis, *H*_0_, after 50 years of breeding with each strategy and use of genomic selection with cycle length of 2. Colored lines indicate strategy and grey bands indicate the standard error of their predicted means. Colored dots indicate the corresponding strategy raw data points. (colour figure online)

For the clonal diploids, use of the best GS strategy increased genetic gain compared to the best PS strategy with fast multiplication after 50 years (Fig. 4). If GS was not used to reduce cycle length, and all strategies were compared at 25 cycles, then at small values of *H*_0_, the best PS strategy produced more gain and the best GS strategy produced more gain with greater *H*_0_ (Supplemental Fig. 6). This shows that the relative performance of GS and PS depends on their relative cycle length as well as *H*_0_. For the clonal autopolyploids, at high intensity the best GS strategy was better than or equal to the best PS strategy (Fig. 4). The advantage of GS decreased as *H*_0_ decreased. At low intensity in autotetraploids, the best GS strategy was indistinguishable from the best PS strategy. At low intensity in autohexaploids, PS outperformed GS if *H*_0_ was low, and vice versa if *H*_0_ was high.

With use of true values, trends in strategy rankings were generally similar as with use of genomic estimated values (Supplemental Figs. 2–5). The notable exception was that the relative performance of Two-Pool Breeding Value scenarios tended to decrease with use of true vs. genomic estimated values.

Less absolute genetic gain was observed as *H*_0_ increased (Figs. 3, 4; Supplemental Figs. 2–5). Based on the slopes of the regression lines, one-pool strategies were more sensitive to *H*_0_ than two-pool strategies (Supplemental File 2; Figs. 3, 4). As genetic gain increased due either to a longer time horizon or higher intensity, the sensitivity of genetic gain to *H*_0_ also increased.

### Additive and dominance value in the core strategies

Regardless of ploidy level, strategy, selection intensity, or timepoint, the regression of additive value on *H*_0_ produced a negative slope, while the regression of dominance value on *H*_0_ produced a positive slope (Supplementary File 2; Supplemental Figs. 7–10). If no dominance was simulated, then both dominance value and *H*_0_ were always zero. In general, one-pool strategies produced more additive value than two-pool strategies regardless of ploidy, timepoint, or intensity (Supplemental Figs. 7, 9). In diploids, Two-Pool GCA produced more dominance value than other strategies at high but not low intensity and as timepoint increased, particularly with use of GS (Supplemental Fig. 8). In autopolyploids, there was typically little difference in dominance value among strategies (Supplemental Fig. 10).

### Inbreeding coefficient with the core strategies

Within a given ploidy level, estimation method, and timepoint, the regression of inbreeding coefficient on *H*_0_ for each strategy sometimes differed depending on the selection intensity (Supplemental File 2; Supplemental Figs. 11–16). Regardless of strategy and ploidy, strategies typically had higher inbreeding coefficients with high selection intensity and lower inbreeding coefficients with low selection intensity across *H*_0_ values or very small differences in inbreeding coefficient, with some exceptions where inbreeding was measured in the inter-pool hybrids (Supplemental Figs. 11–16).

### Inbreeding depression with the core selection strategies

Subsequent to the simulation of an initial amount of population inbreeding depression (heterosis), the amount of inbreeding depression in the population potentially could change as allele frequencies changed due to selection and drift. However, for the studied time horizons, small changes in population inbreeding depression were observed in response to selection, even as genetic gain was positive (Supplemental Figs. 17–20). In general, with comparisons at the same number of cycles, the amount of inbreeding depression for a given ploidy level, estimation method, intensity, and timepoint did not dramatically differ by strategy although some differences were detected (Supplemental Figs. 17–20). Greater reduction of population inbreeding depression was not necessarily associated with greater genetic gain.

### Panmictic heterosis with the core selection strategies

Panmictic heterosis was zero for the one-pool strategies by definition. For the two-pool strategies, the regression of panmictic heterosis on *H*_0_ produced positive slopes, indicating that the amount of panmictic heterosis strategies built increased with the amount of *H*_0_ regardless of ploidy (Supplemental File 2; Fig. 5). Two-Pool GCA tended to build more panmictic heterosis than Two-Pool Breeding Value, and their relative difference decreased as *H*_0_ decreased. In general, Two-Pool GCA built increasingly more panmictic heterosis than Two-Pool Breeding Value as selection intensity and timepoint increased. However, the difference in panmictic heterosis between Two-Pool GCA and Two-Pool Breeding Value decreased as ploidy level increased.

### Breeding value + GCA strategies

Strategies in which intra-pool evaluation was used to advance genotypes to inter-pool crossing, Two-Pool Breeding Value + GCA and Two-Pool Doubled Haploid Breeding Value + GCA, showed increased genetic gain with PS and unchanged genetic gain with GS compared to strategies without intra-pool evaluation, Two-Pool GCA and Two-Pool Doubled Haploid GCA (Supplemental Fig. 21). The same pattern was observed across ploidies for Two-Pool Breeding Value + GCA and Two-Pool GCA. More interestingly, with selection on GCA, intra-pool genetic value tended to decrease over cycles (compared to the initial intra-pool genotypes) regardless of whether intra-pool evaluation was used at high *H*_0_ (Supplemental Fig. 22). However, intra-pool genetic value tended to increase over cycles at low *H*_0_. Intra-pool evaluation increased intra-pool genetic values compared to its absence with PS and fast multiplication without use of doubled haploids, but intra-pool evaluation had no effect on intra-pool genetic values with GS or with PS and use of doubled haploids (Supplemental Fig. 22).

### Doubled haploid GCA strategies

The use of intra-pool fully inbred lines generally led to unchanged genetic gain after 50 years with GS, but in some cases increased genetic gain with PS. (Supplemental Fig. 21). With PS, Two-Pool Doubled Haploid GCA increased gain compared to Two-Pool GCA but had similar performance to Two-Pool Breeding Value + GCA and Two-Pool Doubled Haploid Breeding Value + GCA (Supplemental Fig. 21). Intra-pool fully inbred lines typically had lower mean genetic values than intra-pool outbred clones in both the short term and long term (Supplemental Fig. 22). The difference in doubled haploid and outbred intra-pool genotypes was greater as *H*_0_ increased as they suffered additional inbreeding depression (Supplemental Fig. 22). Population inbreeding depression typically did not differ between Two-Pool Doubled Haploid GCA and Two-Pool GCA, nor between Two-Pool Doubled Haploid Breeding Value + GCA and Two-Pool Breeding Value + GCA (Supplemental Fig. 23).

## Discussion

Although Two-Pool GCA sometimes provided substantially greater rates of genetic gain per unit cost than other strategies in clonal diploids, its relative performance depended on heterosis and inbreeding depression due to dominance in the trait population, the time horizon, the selection intensity in the program, the relative achievable cycle lengths among strategies, the estimation method, ploidy level, and their interactions (Figs. 3, 4). The use of GS rather than PS drastically increased the competitiveness of Two-Pool GCA, indicating that GS unlocks novel opportunities to utilize heterosis (Fig. 3). Increased selection intensity increased the relative performance of Two-Pool GCA to other strategies, perhaps indicating that Two-Pool GCA is more competitive at higher inbreeding rates (Figs. 3, 4). In typical diploid programs with high selection intensities, if Two-Pool GCA could achieve equal cycle lengths as other strategies, then Two-Pool GCA tended to increase the rate of genetic gain per unit cost at lower amounts of *H*_0_ than if Two-Pool GCA required a longer cycle length (Fig. 4). However, in autopolyploids, Two-Pool GCA usually did not increase the rate of genetic gain compared to One-Pool Breeding Value or One-Pool Cross Performance (Fig. 4). Autopolyploid Two-Pool GCA tended to provide an advantage in genetic gain at higher values of *H*_0_ than in diploids, if at all, and the amount of relative increase was less than in diploids (Fig. 4). As in other studies, the use of GS tended to increase gain compared to PS likely due to increased accuracy, faster inbreeding, and decreased cycle length across *H*_0_; use of GS to reduce of the cycle length was a determining factor in whether it outperformed PS at the heritabilities used (Fig. 4; Supplemental Figs. 4, 6; Powell et al. 2020; Gaynor et al. 2017; Heslot et al. 2015; Heffner et al. 2010; Longin et al. 2015).

### Clonal diploids

In clonal diploids, Two-Pool GCA appeared to outperform other strategies in some conditions because of its exceptional ability to increase the dominance value of *F*_1_ hybrid populations, as well as the additive value (Figs. 3, 4, 5). Fundamentally, this is because use of Two-Pool GCA can increase not only the frequency of favorable alleles but also the frequency of heterozygote genotypes relative to HWE in *F*_1_ hybrids of two pools, leading to panmictic heterosis (Lamkey and Edwards 1999). The latter is achieved by selection on GCA, which differs from breeding values in a single pool because dominance value (*d*) is weighted by allele frequencies in the opposite pool (Schnell 1965; Rembe et al. 2019). Selection on GCA drives apart allele frequencies between pools, which results in a sustained increase in heterozygosity over cycles and therefore increased dominance value in the *F*_1_ hybrids. Although both additive and dominance value are transmissible with selection on breeding value and random mating in a single pool, the frequency of heterozygotes is limited by HWE, which is overcome by non-random mating in two pools and selection on GCA (Hardy 1908; Weinberg 1908). It is perhaps worth noting that for a fully additive trait, intra-pool breeding value and GCA are equal. This may explain why RRS can perform similarly to one-pool strategies on average even in the absence of population heterosis: as long as their cycle lengths are equal, the primary drawback of RRS is its additional cost. Generally, the advantages of Two-Pool GCA in clonal diploids increase as:

- The amount of *H*_0_ due to dominance increases, because ability to increase dominance value becomes relatively more important (Fig. 3)
- The time horizon increases, because selection over breeding cycles increases the divergence of allele frequencies between heterotic pools (Fig. 3)
- Its relative cycle length to the other strategies decreases, because cycle length directly impacts the rate of genetic gain, and Two-Pool GCA has a necessarily longer cycle length than the other strategies with PS but not GS (Fig. 4)
- The selection intensity increases, perhaps because higher selection intensities lead to more inbreeding which lead to greater reductions in heterozygosity due to selection and drift which are better alleviated by GCA compared to other strategies, or because higher selection intensities more rapidly drove apart allele frequencies between pools (Figs. 3, 4)
- Its relative cost to the other strategies decreases; however, we did not investigate different levels of relative cost among strategies because this was demonstrated by Longin et al. (2014) and its particulars are highly program-specific.

The amount of trait population heterosis can be estimated experimentally in breeding populations, but it is typically unknown (Pocrnic et al. 2022). Better methods and increased effort to estimate heterosis in breeding programs would be useful to inform decision-making. However, for clonal diploids which can utilize rapid-cycling GS, the benefit of Two-Pool GCA was robust to *H*_0_ under the study assumptions (Figs. 3, 4). Two-Pool GCA provided the most gain over most *H*_0_ values and timepoints surveyed, and if *H*_0_ was relatively low Two-Pool GCA only modestly decreased gain in the short term (Figs. 3, 4). Programs for which Two-Pool GCA is relatively more expensive than assumed here may require more *H*_0_ to reap its benefit (Longin et al. 2014). In contrast to GS, moving to Two-Pool GCA without adequate population heterosis or time presented a risk of decreased genetic gain for phenotypic programs (Fig. 4). Clonal crops using PS with a low multiplication ratio never benefited from Two-Pool GCA over the time horizons in the study, highlighting this consideration for clonal species and the usefulness of efforts to increase the multiplication ratio (Supplemental Figs. 2, 3; Aighewi et al. 2015). It would be useful to confirm the optimal GS strategies for programs with low multiplication ratios, particularly with multiple cohorts running in parallel per season. Please see Supplemental File 4 for discussion of Two-Pool Breeding Value and One-Pool Cross Performance in diploids, which may be useful for programs which cannot transition to Two-Pool GCA.

Reducing population heterosis (inbreeding depression) was neither required nor a strategic advantage to make genetic gain, and exhaustion of genetic variance due to drift and selection typically occurred well before substantial changes in population heterosis or inbreeding depression were observed. It is a common misconception that RRS, particularly with use of inbred parents, more effectively purges inbreeding depression that other strategies; we show that it does not (Supplemental Figs. 17–20; Ceballos et al. 2020). Rather, RRS effectively masks deleterious recessive alleles.

### Intra-pool evaluation in diploid RRS

In applied diploid inbred-hybrid RRS programs of seed crops, intra-pool genotypes are often first selected as parents of hybrids on their per-se value (Lee and Tracy 2009). In clonal crops with relatively lower multiplication ratios, increased performance of intra-pool parents may not drastically increase hybrid propagule or seed production, so it was unclear whether resource allocation to intra-pool evaluation is efficient. For the costs and proportions of individuals advanced assumed in the study, we observed that a round of intra-pool advancement on breeding value before intra-pool recycling on GCA typically increased genetic gain with PS or did not change the rate of genetic gain with GS in the inter-pool hybrids (Supplemental Fig. 21). As such, breeders likely have some flexibility in whether to conduct intra-pool evaluation. For the GS scenarios here, it was likely suboptimal to predict intra-pool breeding values from a training set of inter-pool individuals, and predicting intra-pool breeding values from intra-pool individuals may increase genetic gain (Wei and Van der Werf 1994; Moghaddar et al. 2014; Hidalgo et al. 2016). For the PS scenarios, increased genetic gain was likely due to increased mean value of the advanced intra-pool individuals and testers, but intra-pool evaluation may have also increased the inbreeding rate.

Interestingly, the effect of recycling on GCA on intra-pool (non-inbred) mean value over cycles depended on *H*_0_: it tended to decrease intra-pool value as *H*_0_ increased but increase intra-pool value as *H*_0_ decreased (Supplemental Fig. 22). In the absence of dominance, intra-pool breeding value is equal to GCA, so intra-pool genotypes selected for GCA are nearly the same as those which would be selected on breeding value at low *H*_0_ (Rembe et al. 2019). This likely led to increases in intra-pool genetic value. As dominance increases, and as allele frequencies differ between pools, the values of intra-pool breeding value and GCA diverge. At high *H*_0_, selection on GCA led the parental pools to suffer inbreeding depression as they were driven to homozygous states, thus decreasing their value over breeding cycles. Conducting intra-pool advancement on breeding value sometimes slightly increased intra-pool parents’ value compared to forgoing intra-pool evaluation (Supplemental Fig. 22). However, at the proportion of individuals advanced (75%), intra-pool selection did not prevent decrease in intra-pool value when population heterosis was high. In practice, if population heterosis is high and it is necessary to maintain or increase intra-pool value with Two-Pool GCA, it may be necessary to select intra-pool parents more stringently on their breeding values or even to recycle intra-pool parents on an index of intra-pool breeding value and GCA (Longin et al. 2007). This is in contrast to trends in inbred-hybrid RRS programs, where intra-pool fully inbred line values typically increase due to increases in additive value and the intrinsically zero dominance value is not lost (Troyer and Wellin 2009).

### Fully inbred parents in diploid RRS

A concern in clonal diploids is whether RRS programs benefit from using fully inbred parents, as is done in other species. With all else equal, it is expected that inbreeding depression (loss of baseline heterosis) suffered in the intra-pool parents is fully reversed in the inter-pool hybrids, as well as the addition of the panmictic heterosis value, so intra-pool inbreeding is unnecessary to harness heterosis. We did not observe differences in genetic gain with use of inbred parents and GS in RRS (Supplemental Fig. 21). With PS, use of inbred parents increased genetic gain only if intra-pool evaluation was not used, and use of intra-pool evaluation generated comparable gains as using inbred parents; this appeared to be due to decreased accuracy in phenotypic estimation of Two-Pool GCA, perhaps due to Mendelian sampling of the outbred tester in the progeny (Supplemental Figs. 21, 24–26).

We also demonstrate that RRS with or without inbred parents does not substantially reduce population inbreeding depression (Supplemental Figs. 17–20). There have been substantial efforts to inbreed species such as cassava in order to reduce genetic load specifically, and it is clear that these are misguided (Ceballos et al. 2020). Clonal breeders can make effective genetic gain in the presence of inbreeding depression by masking deleterious alleles rather than purging them. However, our results also suggest that populations with lower *H*_0_ have higher genetic gain overall. As such, if a choice exists between populations with similar means and variances but differing inbreeding depression, the population with less inbreeding depression would be expected to have a higher rate of genetic gain (Figs. 3, 4).

It has been proposed that use of inbred parents could enable seed systems in clonal crops and reduce the cost of propagation, the time and cost required to transport clones across national borders, and the spread of disease (McKey et al. 2010; Ceballos et al. 2015). These are worthy considerations that are considered externalities in the current study, but they are completely independent of the use of RRS and could equally be availed in one-pool strategies. Programs considering line development should thoroughly assess their germplasm’s tolerance of full inbreeding as well as the trade-offs in time and resources needed for line development. The cost and time to generate inbred lines are likely higher than assumed in our study, given that doubled haploid technologies do not exist for most clonal species. Furthermore, the simulated inbred line values may correspond to total non-viability in some species or populations, especially those with high population inbreeding depression.

### Clonal autopolyploids

In contrast to clonal diploids, Two-Pool GCA typically did not outperform other strategies in clonal autopolyploids (Fig. 4). Instead, One-Pool Breeding Value or One-Pool Cross Performance was the safest option depending on *H*_0_ (Fig. 4). A larger range of *H*_0_ values were considered in autopolyploids than diploids; RRS did not benefit autopolyploids at the same and some greater amounts of *H*_0_ which benefited diploids (Fig. 4; Supplemental Fig. 1). This is likely because autopolyploids inherit multiple chromosome copies per gamete, and therefore, autopolyploids sustain greater heterozygosity across all gametes, genotypes, and matings at segregating loci even in response to selection on One-Pool Breeding Value or cross performance (Supplemental Fig. 27; Bartlett and Haldane 1934; Bever and Felber 1992). The relative advantage of Two-Pool GCA in diploids is due to its ability to increase heterozygosity of inter-pool populations at loci with dominance. Because the frequency of heterozygotes compared to homozygotes at segregating loci in autopolyploid populations is already relatively high compared to diploids, there is not only less value to be gained by increasing heterozygote frequency with Two-Pool GCA but also less value lost to the smaller increase in deleterious recessive homozygote frequency under selection on One-Pool Breeding Value (Supplemental Fig. 27, Supplemental Table 3). Although this study considered clonal species, these conclusions should be applicable to non-clonal autopolyploids. It may be worth noting that the lack of advantage to selection on Two-Pool GCA only applies to autopolyploids, not to allopolyploids for which chromosome copies are not independently assorted.

Consistent with this hypothesis, the relative overperformance of one-pool strategies compared to Two-Pool GCA was greater in autohexaploids than autotetraploids: autohexaploids inherit more chromosome copies (3) per gamete than autotetraploids (2), leading to greater heterozygosity at segregating loci (Supplemental Fig. 27). We expect that the relative genetic gain per unit cost of Two-Pool GCA to One-Pool Breeding Value would be further reduced at higher autoploidies. Another line of support for this hypothesis was that the relative performance of Two-Pool GCA to other strategies increased with GS at high intensity (Fig. 4). High-intensity GS often had a higher inbreeding coefficient and probably more genetic drift compared to low-intensity GS or high-intensity PS, so the ability of Two-Pool GCA to relieve homozygosity became more important (Supplemental Figs. 13–16). However, One-Pool Cross Performance was similarly capable of relieving inbreeding in this situation and is less logistically demanding (Fig. 4). Finally, Two-Pool GCA built more panmictic heterosis than Two-Pool Breeding Value, but the difference was less in autopolyploids than diploids (Fig. 5). This indicates breeding for heterosis with GCA was less effective in autopolyploids, since it more narrowly outperformed incurrence of heterosis due to drift.

It is tempting to assume that an observation of heterosis implies that RRS is the optimal breeding scheme, but in light of ploidy, this does not seem to be the case. Autopolyploids can exhibit panmictic heterosis even though they do not appear to benefit from RRS (Fig. 5). Selection on Two-Pool GCA or Two-Pool Breeding Value led to clear panmictic heterosis in the autopolyploids simulated in the study. Empirical evidence of panmictic heterosis in autohexaploid sweetpotato, for example, is readily available for fresh root yield (Diaz et al. 2021). The existence of panmictic heterosis in autohexaploids does not imply that Two-Pool GCA or Two-Pool Breeding Value is the optimal breeding strategy for autohexaploids. The observed heterosis in sweetpotato could also be availed by intermating the two pools and subsequently selecting on One-Pool Breeding Value, although further comparisons of strategy efficiencies with pre-existing diverged pools would be informative for all ploidies. In the case of sweetpotato, two pools exhibiting panmictic heterosis emerged when a single breeding population was split into two locations (M. Andrade, pers. comm.). Over approximately twenty years, the pools were selected separately by truncation (W. Gruneberg, pers. comm.), and therefore, allele frequencies likely came to diverge between pools due to selection and drift. Reunion of the pools then led to population-level panmictic heterosis in the *F*_1_ hybrids (Diaz et al. 2021).

The relatively decreased homozygosity of autopolyploids compared to diploids with selection on breeding value does not imply that autopolyploids suffer less inbreeding depression than diploids in the event that they do experience homozygosity of unfavorable alleles. This misconception may arise from failure to differentiate the inbreeding rate and inbreeding depression value. Autopolyploids in fact may experience more inbreeding depression in response to increased homozygosity than diploids, which can be observed in simulated autopolyploids produced by chromosome doubling with digenic dominance (Supplemental Table 4). Although few comparable estimates of inbreeding depression in real data are available, one such dataset is that of Yao et al. (2020), which compared genotypically matched diploid and autotetraploid maize. In a selfing series of each, Yao et al. (2020) observed similar inbreeding depression in the diploids and autotetraploids at the same selfing generation. Since autotetraploids are less inbred than diploids at a given selfing generation, their similar inbreeding depression suggest that autotetraploid inbreeding depression was more severe per unit increase in homozygosity. Of course, it cannot be concluded that the maize autotetraploids used experienced only inbreeding depression due to digenic dominance, and the inbreeding depression observed could be due to loss of higher-order dominance interactions as well.

### Assumptions, limitations, and future research directions

The conclusions of this study depend on the assumptions made and parameters used. Additional discussion of the study assumptions are available in Supplemental File 4. Further exploration of these factors is welcomed, and we encourage breeding programs to simulate and optimize their specific situation when information is readily available. Exploration of ranges of values is helpful to explore factors which affect the relative performance of breeding strategies, but once identified, the number of real-world constraints on breeding programs is much smaller than all possible constraints on breeding programs.

The breeding schemes used are not optimal but are rather a baseline for comparison of population improvement methods. We believe that the most important limitation is that each scenario was not optimized to a given time horizon. A scenario is optimal at a given time horizon if genetic gain is maximized at the time horizon, or the rate of genetic gain is maximized over the time horizon; presumably, these both imply exhaustion of genetic variance at the time horizon (Moeinizade et al. 2019). Closer time horizons typically have higher optimal inbreeding rates and selection intensities than more distant time horizons. Our study clearly demonstrates that strategy relative performance is sensitive to the inbreeding rate/selection intensity (Fig. 4; Supplemental Figs. 11–16). Therefore, for a given time horizon, if a different inbreeding rate/selection intensity is optimal than those assumed in the study, strategy relative performance could differ at the optimal inbreeding rate/selection intensity across genetic architectures. It is possible that further increasing the inbreeding rate in autopolyploids (e.g., by using truncation selection without inbreeding control) could increase the relative performance of Two-Pool GCA to other strategies, but this would not necessarily increase genetic gain overall. This requires further study.

On a similar note, we arbitrarily assumed an equal total number of parents (ETP) per strategy rather than equal number of parents per pool (EPPP) per strategy. Neither of these assumptions ensure that the strategy inbreeding rate is optimal. However, we checked whether using EPPP by decreasing the parents per pool in the one-pool strategies to 20 and 100 at high and low intensity while increasing progeny per cross to maintain equal program size, respectively, led to changes in strategy relative performance (Supplemental Figs. 28, 29). For the GS core scenarios, Two-Pool GCA was still the optimal strategy in diploids after 50 years, whereas one-pool strategies were optimal in autopolyploids. However, using a higher intensity in the one-pool strategies did increase the competitiveness of One-Pool Cross Performance with the two-pool strategies, as One-Pool Cross Performance could more effectively increase additive value while still maintaining dominance value at higher intensity (Supplemental Fig. 28). Additionally, the crossover between one-pool and two-pool strategies in autopolyploids occurred at lower *H*_0_ values with high-intensity EPPP, but the overall increase in genetic gain with the best EPPP versus ETP strategy at a given *H*_0_ was marginal (Supplemental Fig. 28). This indicates the original point that the optimal inbreeding rate/intensity, which our scenarios did not necessarily achieve, differs by time horizon and strategy.

We also did not optimize accuracy within the breeding strategies and estimation methods, which may require different designs for optimal accuracy, but we addressed this by simulating all scenarios with true values to control accuracy. In the true value scenarios, we did not observe radically different trends in strategy relative performance (Supplemental Figs. 2–5). The scenarios with true values have controlled accuracy but less genetic drift than GS scenarios, because true values are like using phenotypes with broad-sense heritabilities of one (Daetwyler et al. 2007; Sonesson et al. 2012). Please find further discussion of the lack of optimization of accuracy in Supplemental File 4.

We assumed biallelic loci. We do not expect that multiallelic loci in autopolyploids would likely lead to increased advantages of Two-Pool GCA, because with linkage disequilibrium haplotypes of biallelic loci effectively behave as a single multiallelic locus. We did not vary the probability of autopolyploid multivalents.

We note that heterosis in autopolyploids is not maximized with single crosses among two diverged pools, i.e., heterosis is progressive (Groose et al. 1989; Washburn and Birchler 2014; Washburn et al. 2019; Labroo et al. 2021). Autopolyploid heterosis due to dominance is progressive because autopolyploids have fewer parents than inherited gametes. If allele frequencies diverge randomly across loci with dominance among parents, additional heterosis occurs by making multi-parental crosses because additional heterozygosity can be stacked into the progeny genome. We do not expect that utilization of progressive heterosis in autopolyploids would change the relative performance of the strategies because the additional heterosis is likely relatively small compared to the potential additional time needed to make additional crosses as well as the resources needed to maintain additional pools. However, testing this hypothesis is warranted. We note that progressive heterosis due to digenic dominance can be observed by the simulation methods of the study (https://github.com/gaynorr/AlphaSimR_Examples/blob/master/misc/ProgressiveHeterosis.R).

As mentioned repeatedly, comparisons of gain across ploidies from simulation should not be made because they are not guaranteed to reflect biological reality. Real data, which are likely population-specific, would be needed. For example, we assume that the minimum homozygote and maximum heterozygote value are the same in diploids and polyploids, but there is evidence that this is unrealistic in some populations because polyploid populations produced by colchicine doubling sometimes have higher mean values than their diploid progenitors (Sattler et al. 2016). In the case of potato, our findings strongly suggest that Two-Pool GCA is not likely to be the optimal breeding strategy for autotetraploid potato, whereas Two-Pool GCA is likely to be the optimal breeding strategy for diploid potato if GS is used or *H*_0_ is adequate. However, we cannot determine from simulation alone whether overall genetic gain per unit cost is likely to be higher in autotetraploid or diploid potato.

## Conclusions

In conclusion, clonal diploid breeding programs for which use of GS is attainable to reduce cycle length, multiplication is fast, costs are similar as assumed, inbreeding rates are similar as assumed at high intensity with maximum avoidance of inbreeding, and time is adequate appear to benefit from using RRS regardless of *H*_0_ over the range surveyed. At some levels of *H*_0_, RRS may drive intra-pool value to the point of non-viability with or without inbred lines, and this should be monitored. Clonal diploid programs which use PS and fast multiplication should use One-Pool Breeding Value rather than RRS unless *H*_0_ is adequate. Clonal diploids using PS and slow multiplication should use One-Pool Breeding Value. RRS programs which utilize intra-pool evaluation typically have increased rates of genetic gain. Under the costing and viability assumptions used, use of inbred intra-pool parents in RRS is optional from the perspective of genetic gain. Clonal autopolyploids should use either One-Pool Breeding Value or One-Pool Cross Performance and do not benefit from two-pool strategies. These conclusions are sensitive to the program inbreeding rate, and therefore targeted time horizon.

## Supplementary Information

Below is the link to the electronic supplementary material.

Supplementary file1 (PDF 87894 kb)

Supplementary file2 (PDF 168 kb)

Supplementary file3 (XLSX 423 kb)

Supplementary file4 (XLSX 6235 kb)

Supplementary file5 (PDF 804 kb)

Supplementary file6 (PDF 1935 kb)

## Data Availability

All code and raw data generated for the study are available at https://github.com/gaynorr/ClonalHybridStrategies. The initial simulated populations used are available upon request.
