# Supplementary material for: Clonal diploid and autopolyploid breeding strategies to harness heterosis: insights from stochastic simulation
Source: Theor Appl Genet. 2023 Jun 8;136(7):147. doi: 10.1007/s00122-023-04377-z (PMC10250475; doi:10.1007/s00122-023-04377-z)
Supplement: Supplementary file 1 — Supplementary file1 (PDF 87894 kb) [file 122_2023_4377_MOESM1_ESM.pdf]

Clonal diploid and autopolyploid breeding strategies to harness heterosis: insights from stochastic simulation. Theoretical and Applied Genetics. Marlee R. Labroo, Jeffrey B. Endelman, Dorcus C. Gemenet, Christian R. Werner, R. Chris Gaynor, Giovanny Eduardo Covarrubias-Pazaran (Excellence in Breeding Platform, Consultative Group of International Agricultural Research; covaruberpaz@gmail.com)

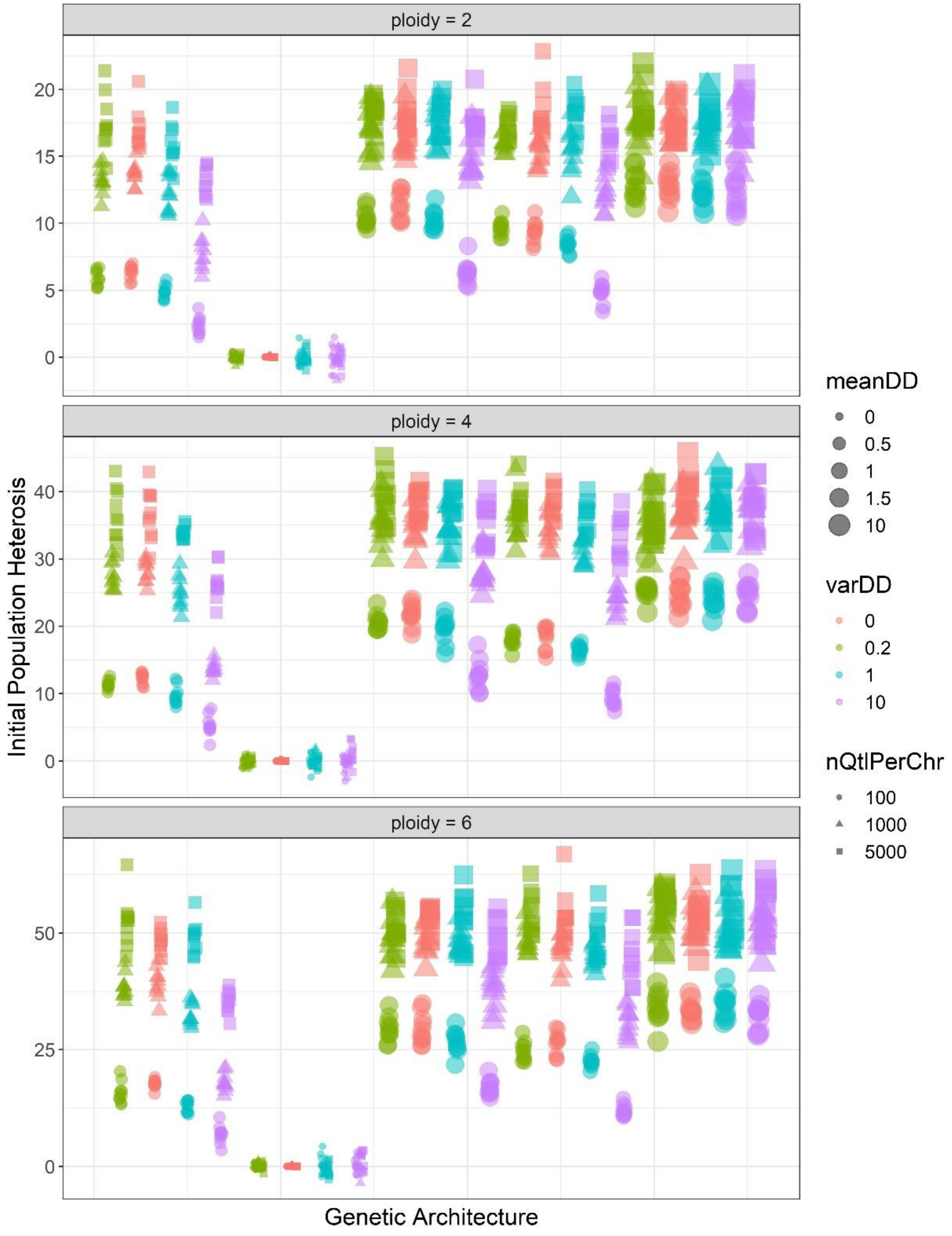

Supplemental Figure 1. Scaled initial population heterosis (H_0) resulting from each combination of ploidy, number of QTL per chromosome (point shape), mean dominance degree (point size), and variance of the dominance degrees (point color). Ten chromosomes were assumed. A larger range of H_0 values were simulated as ploidy increased.

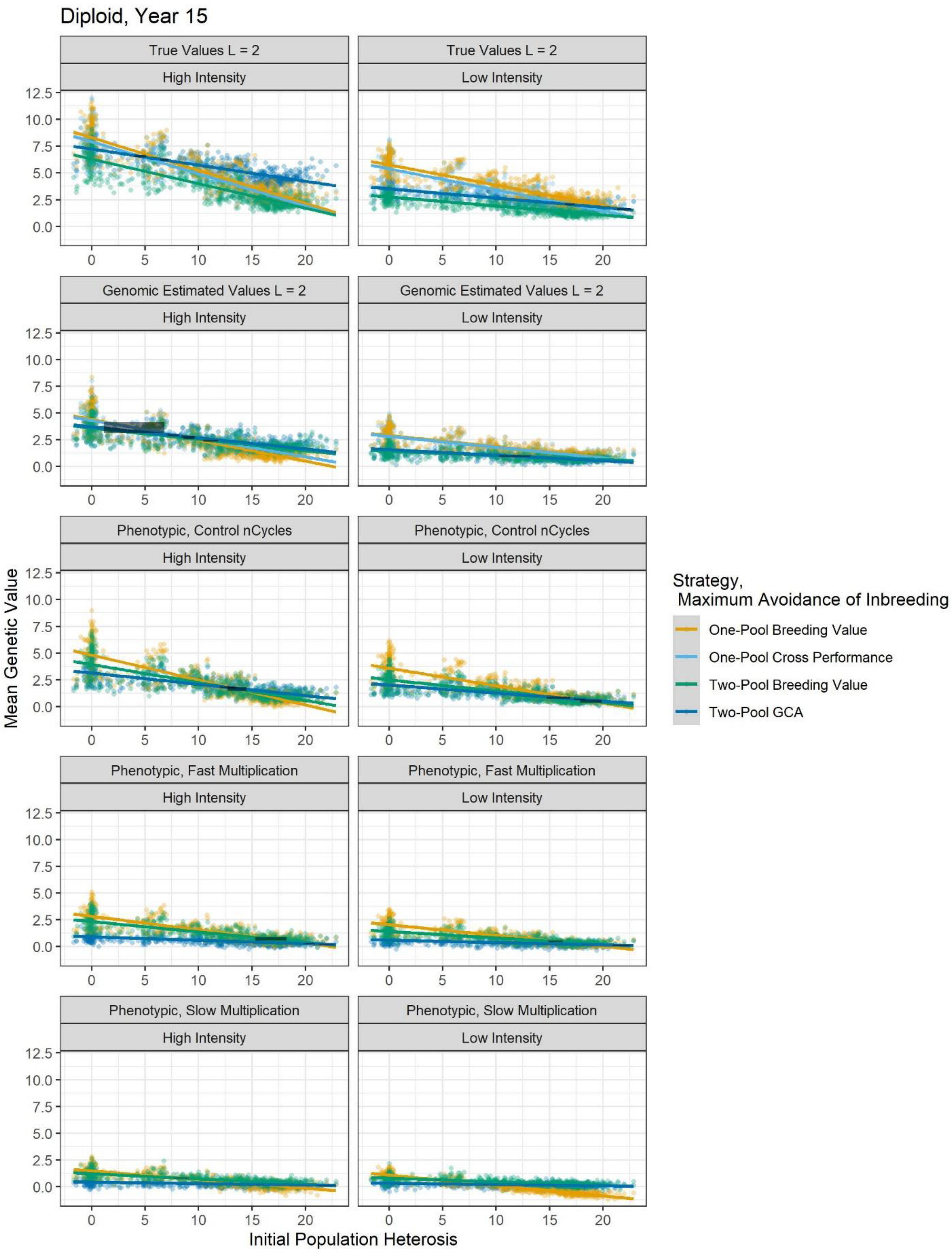

Supplemental Figure 2. Diploid genetic gain in product pool at year 15. Symbols are as described in Fig. 2. Shaded boxes indicate the standard error of the intersections of the strategy regressions, if estimable.

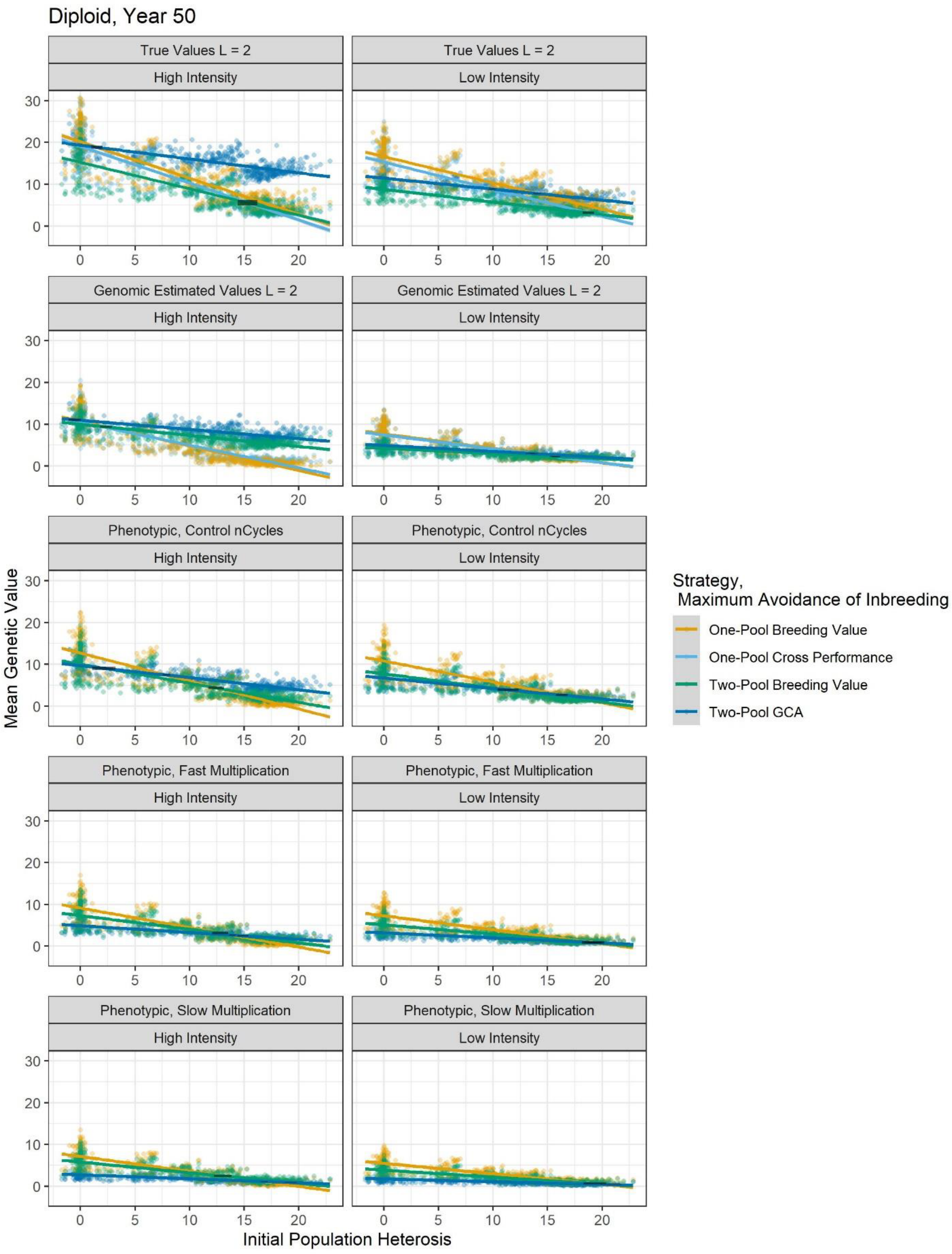

Supplemental Figure 3. Diploid genetic gain at year 50. Symbols are as described in Fig. 2. Shaded boxes indicate the standard error of the intersections of the strategy regressions, if estimable.

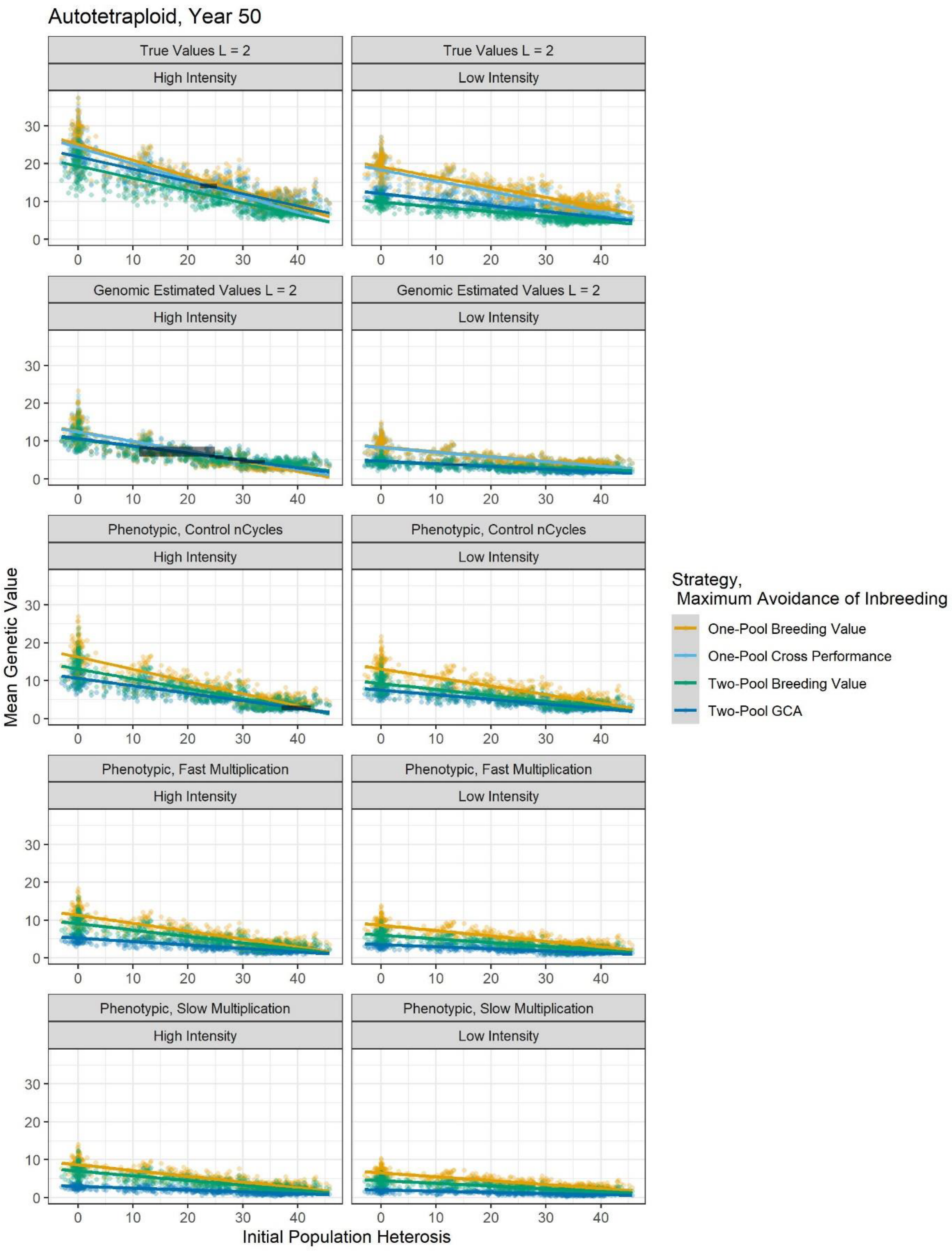

Supplemental Figure 4. Autotetraploid genetic gain at year 50. Symbols are as described in Fig. 2. Shaded boxes indicate the standard error of the intersections of the strategy regressions, if estimable.

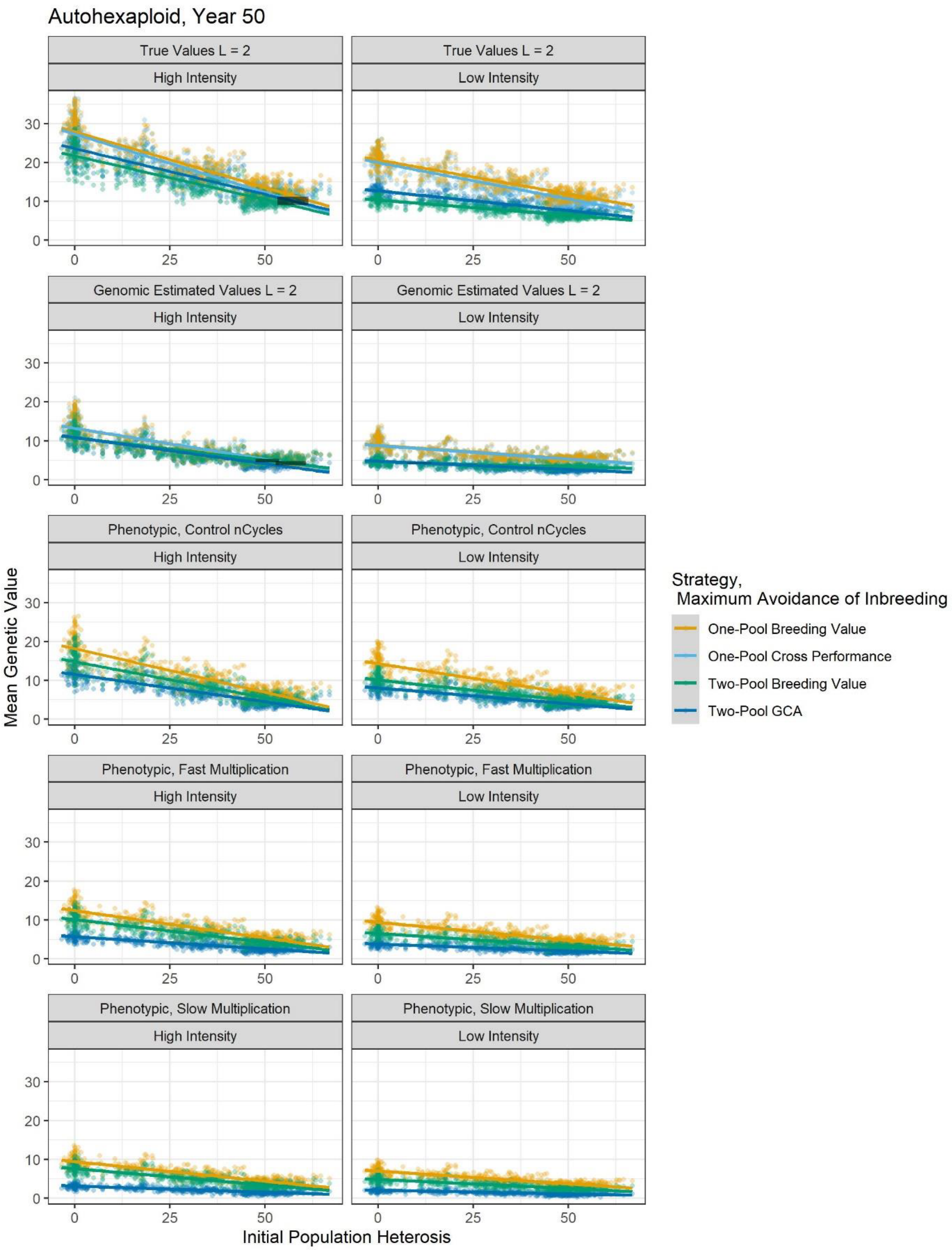

Supplemental Figure 5. Autohexaploid genetic gain at year 50. Symbols are as described in Fig. 2. Shaded boxes indicate the standard error of the intersections of the strategy regressions, if estimable.

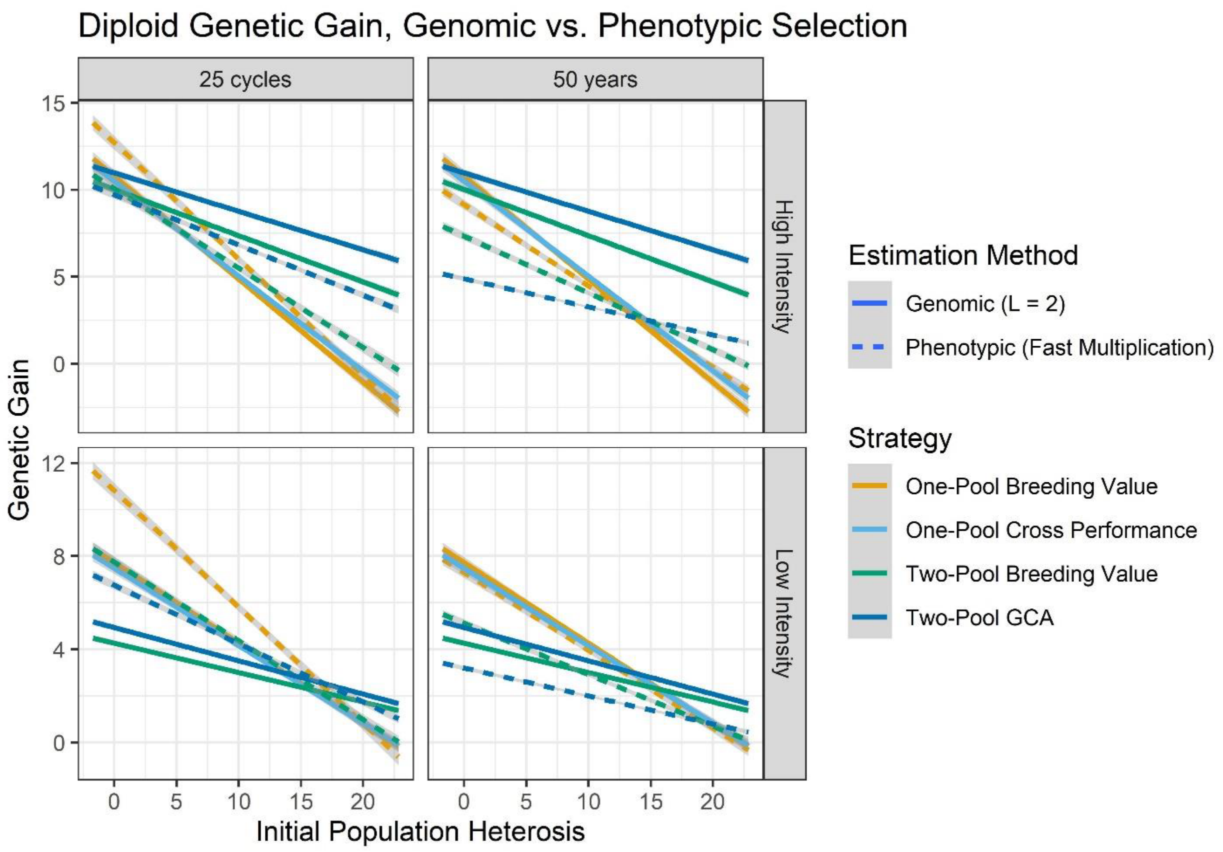

Supplemental Figure 6. Diploid genetic gain with genomic vs. phenotypic selection after a fixed number of cycles (25) and, realistically, after a fixed number of years (50).

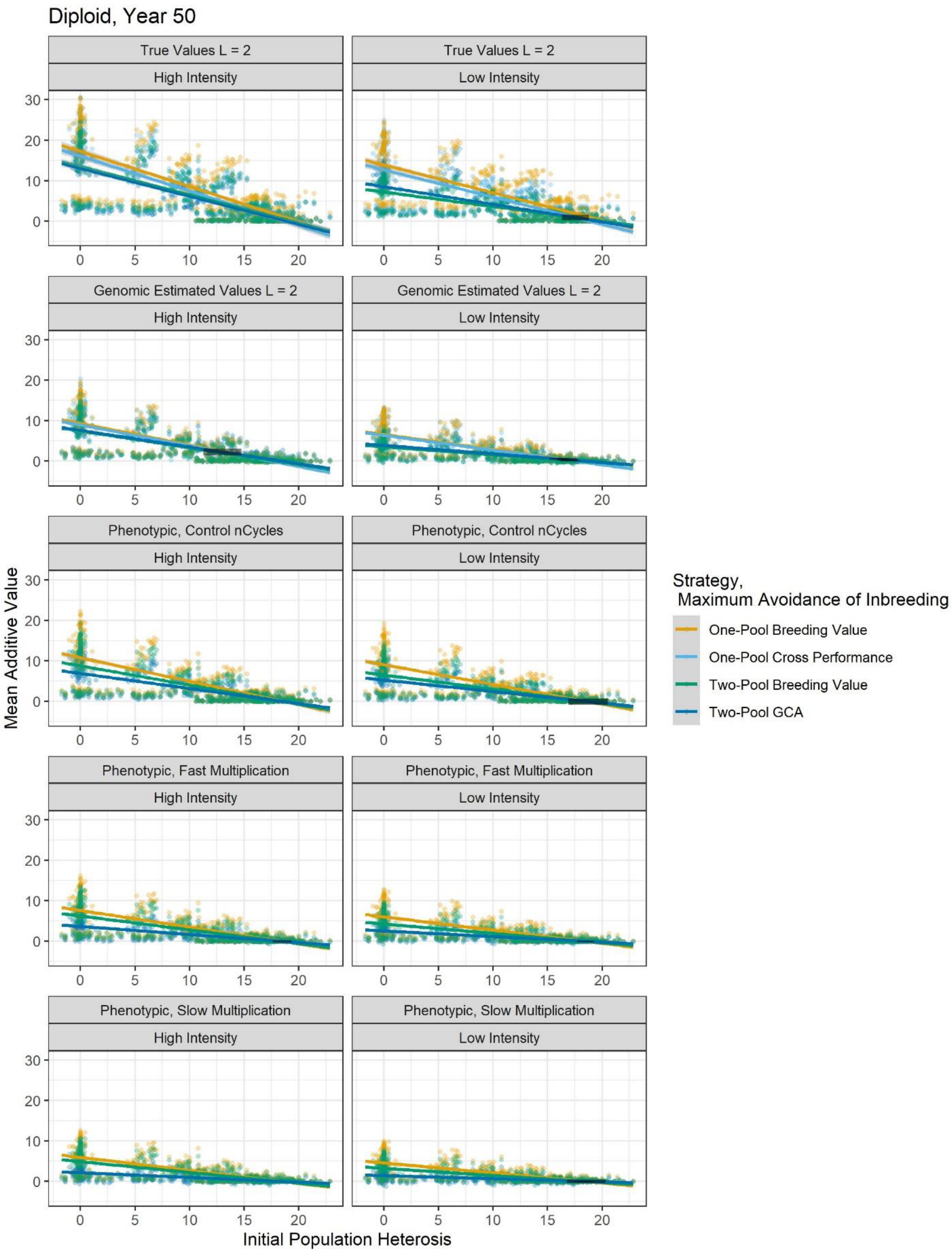

Supplemental Figure 7. Diploid additive value by strategy across H_0 after 50 years of breeding. Shaded boxes indicate the standard error of the intersections of the strategy regressions, if estimable.

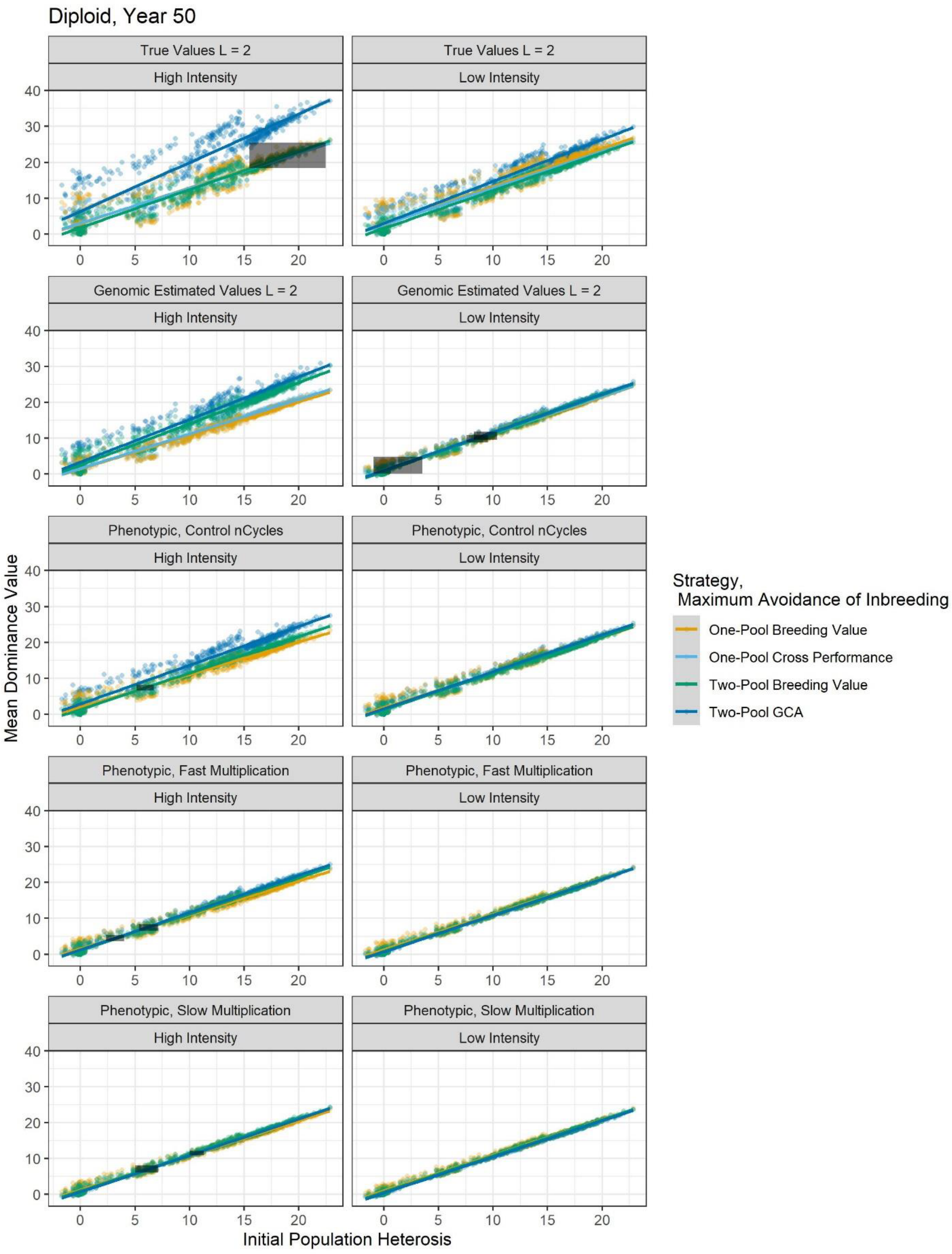

Supplemental Figure 8. Diploid dominance value by strategy across H_0 after 50 years of breeding. Shaded boxes indicate the standard error of the intersections of the strategy regressions, if estimable.

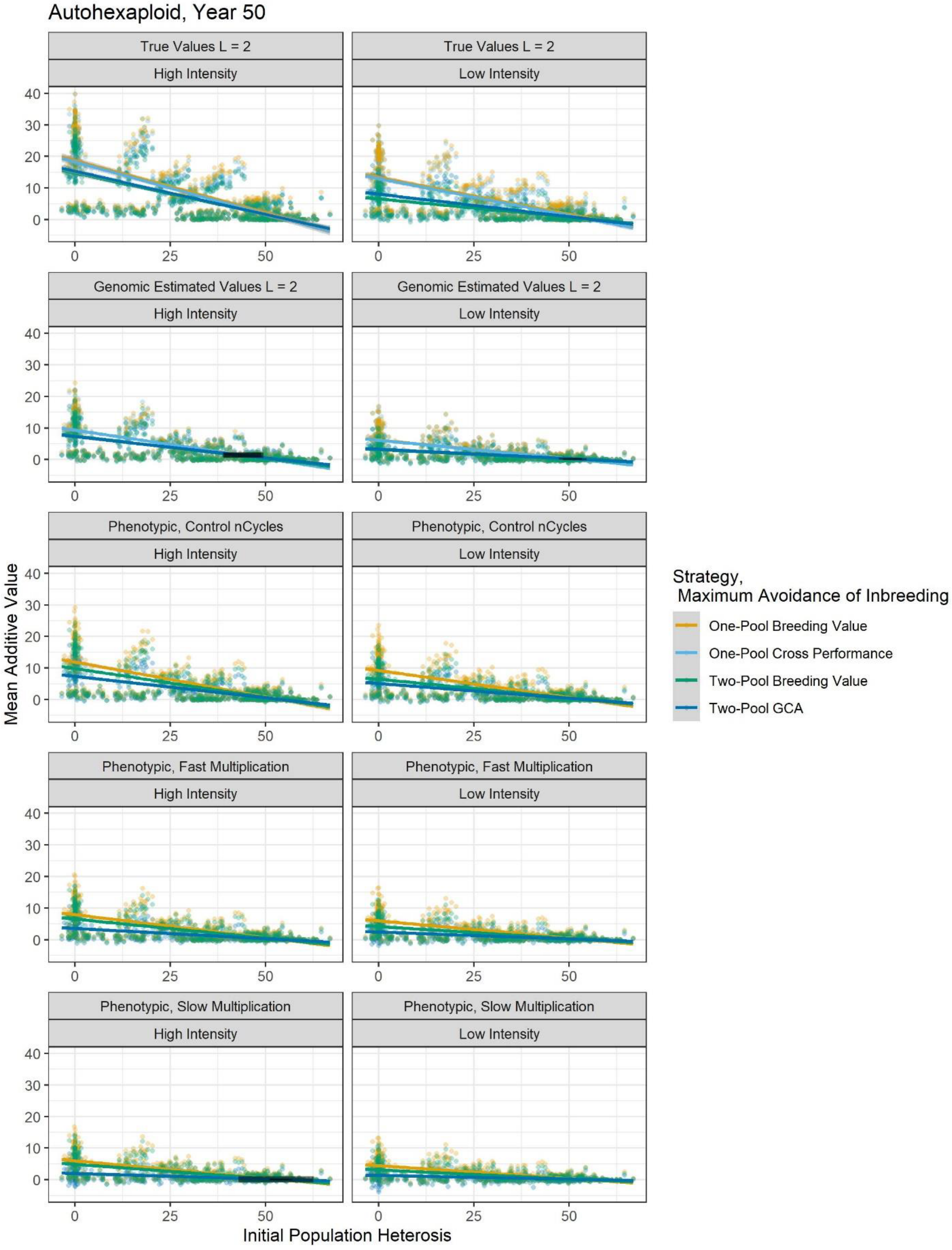

Supplemental Figure 9. Autohexaploid additive value by strategy across H_0 after 50 years of breeding. Shaded boxes indicate the standard error of the intersections of the strategy regressions, if estimable.

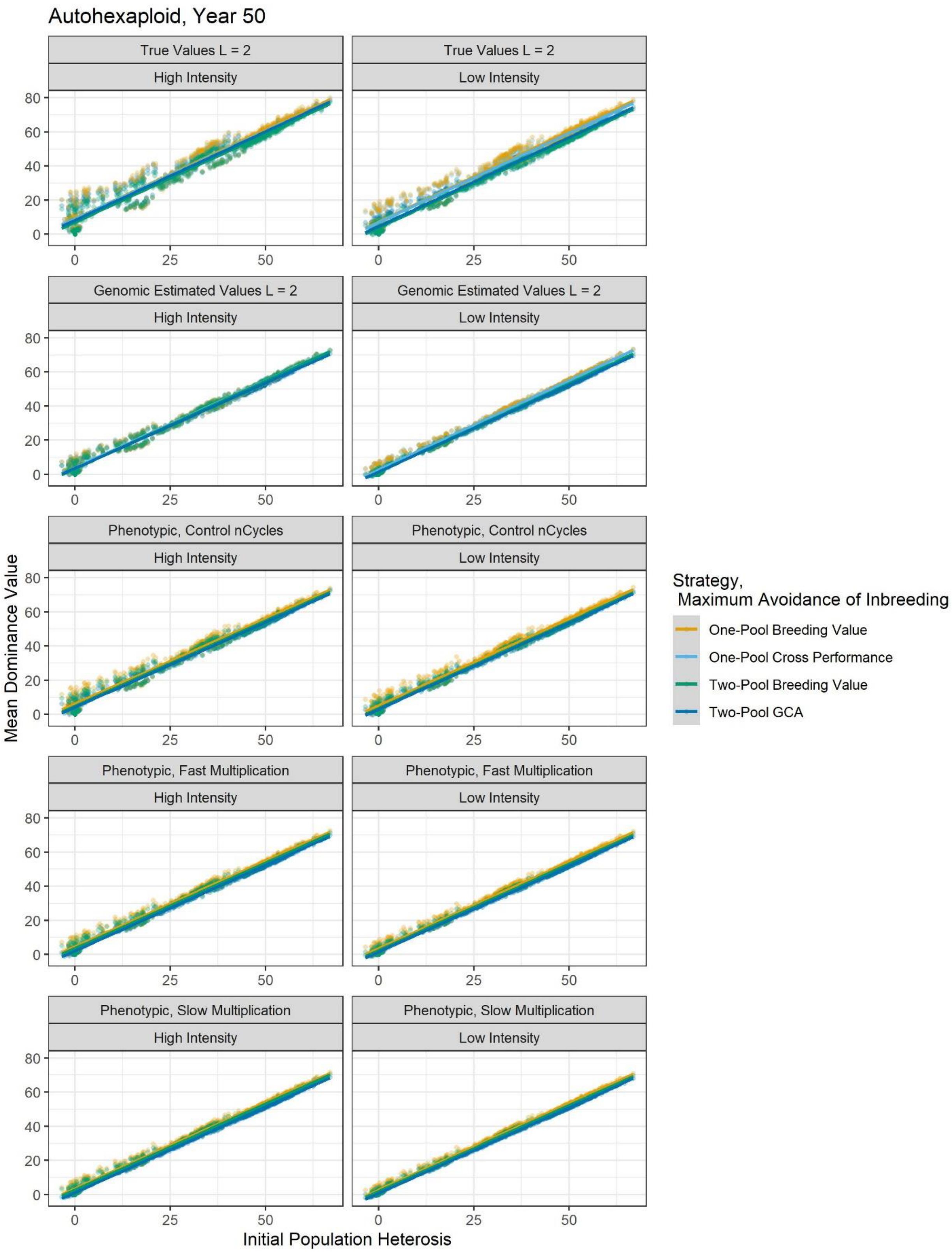

Supplemental Figure 10. Autohexaploid dominance value by strategy across H_0 after 50 years of breeding. Shaded boxes indicate the standard error of the intersections of the strategy regressions, if estimable.

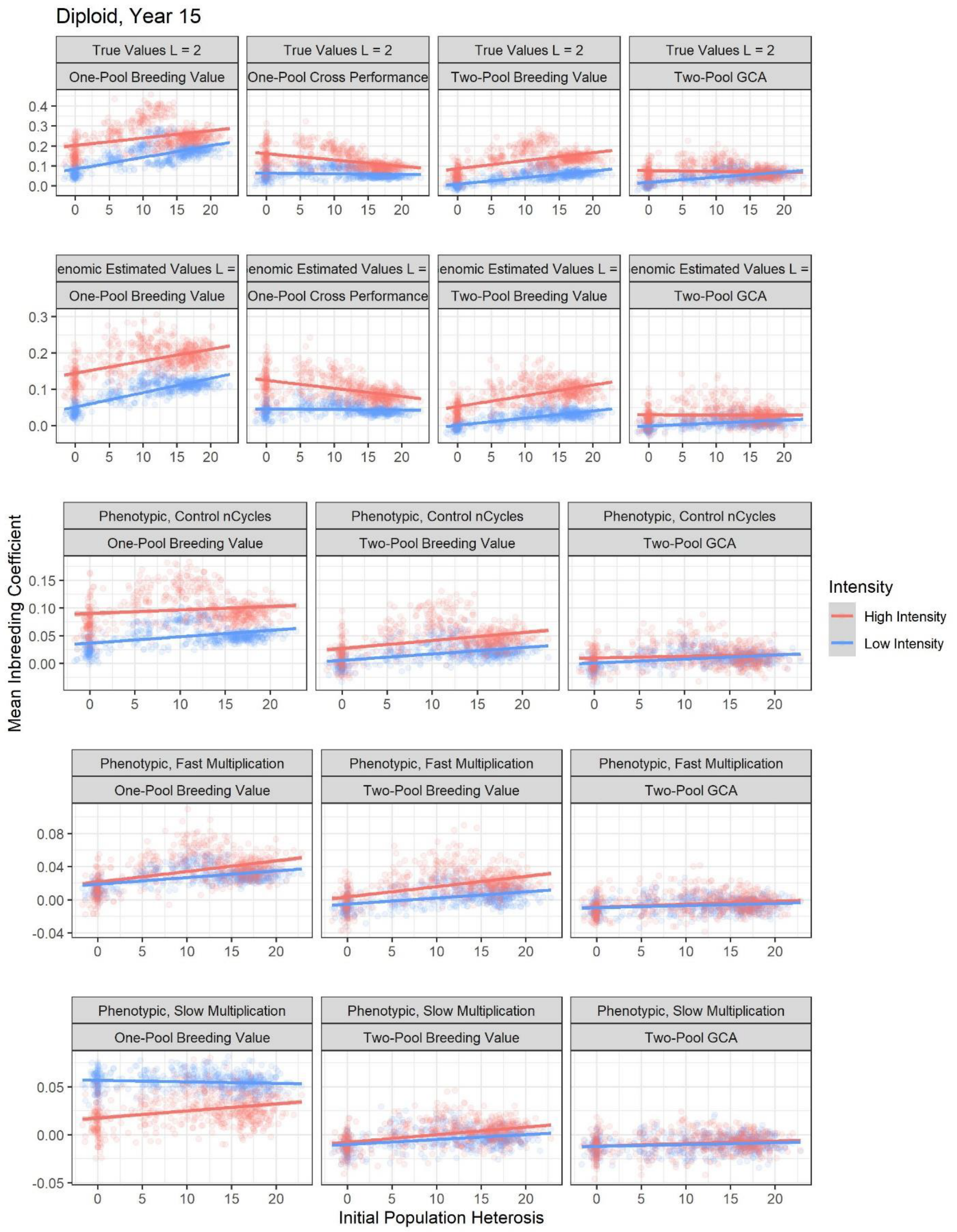

Supplemental Figure 11. Mean inbreeding coefficient in diploids by estimation method, strategy, and intensity as a function of H_0 after 15 years.

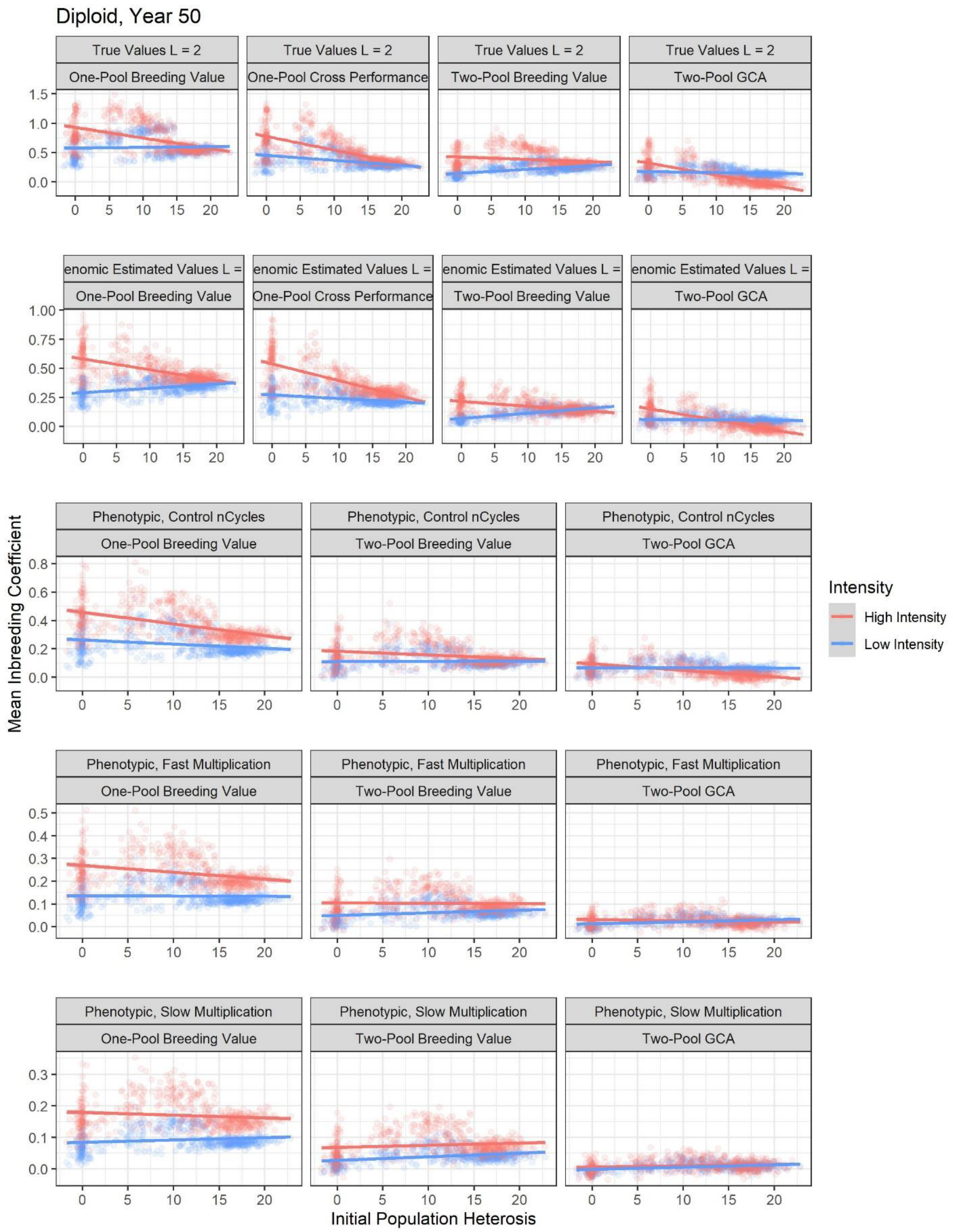

Supplemental Figure 12. Mean inbreeding coefficient in diploids by estimation method, strategy, and intensity as a function of H_0 after 50 years.

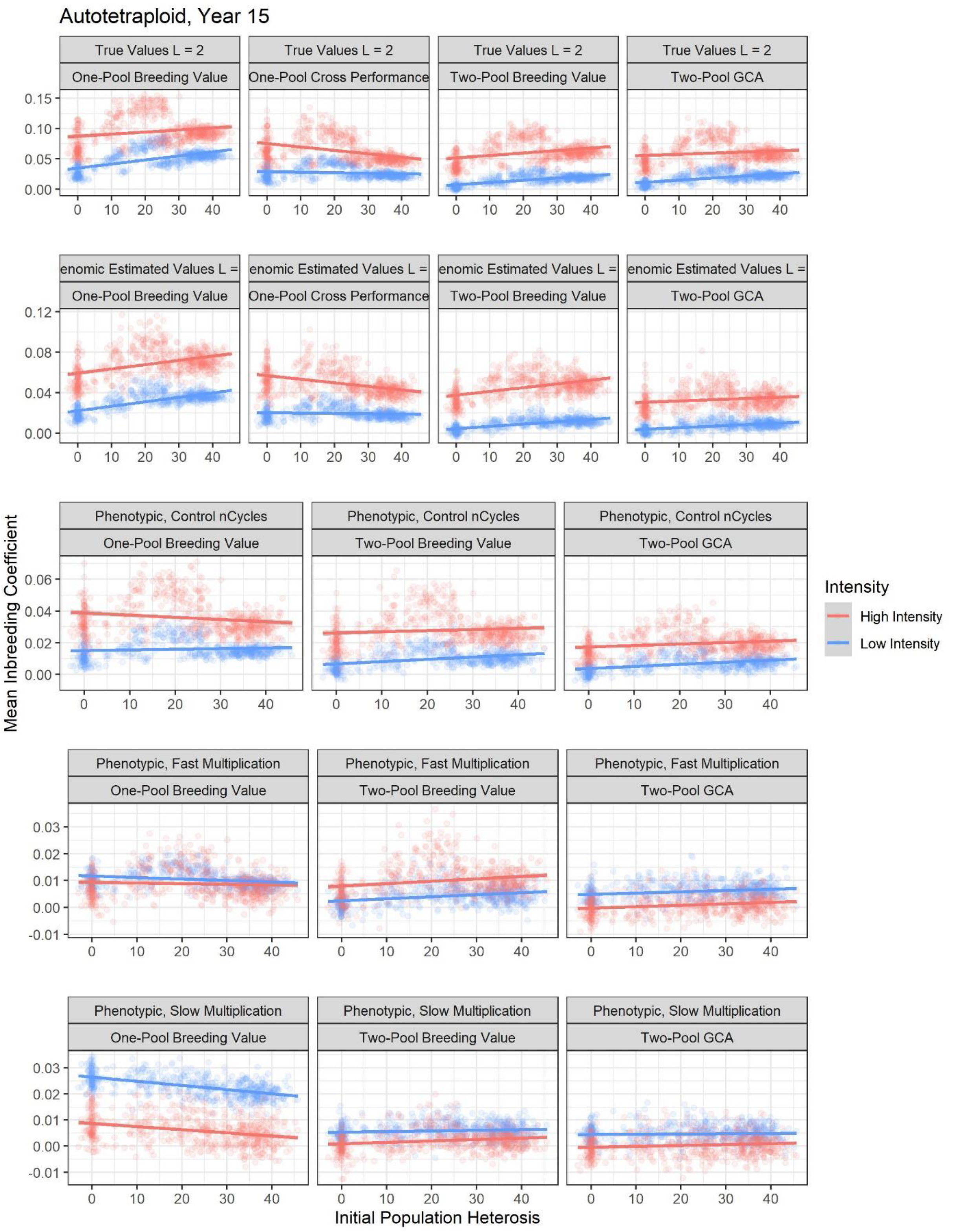

Supplemental Figure 13. Mean inbreeding coefficient in autotetraploids by estimation method, strategy, and intensity as a function of H_0 after 15 years.

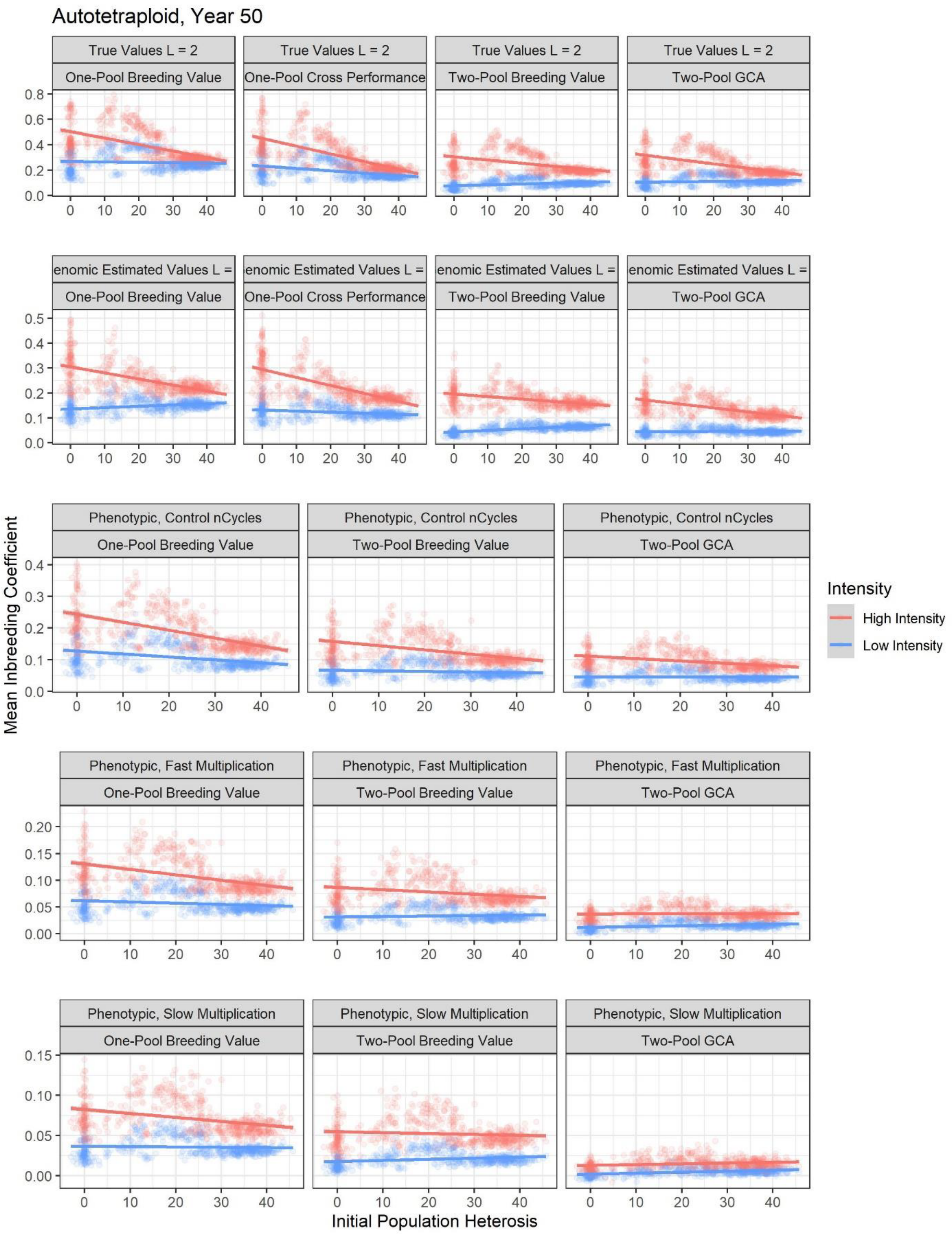

Supplemental Figure 14. Mean inbreeding coefficient in autotetraploids by estimation method, strategy, and intensity as a function of H_0 after 50 years.

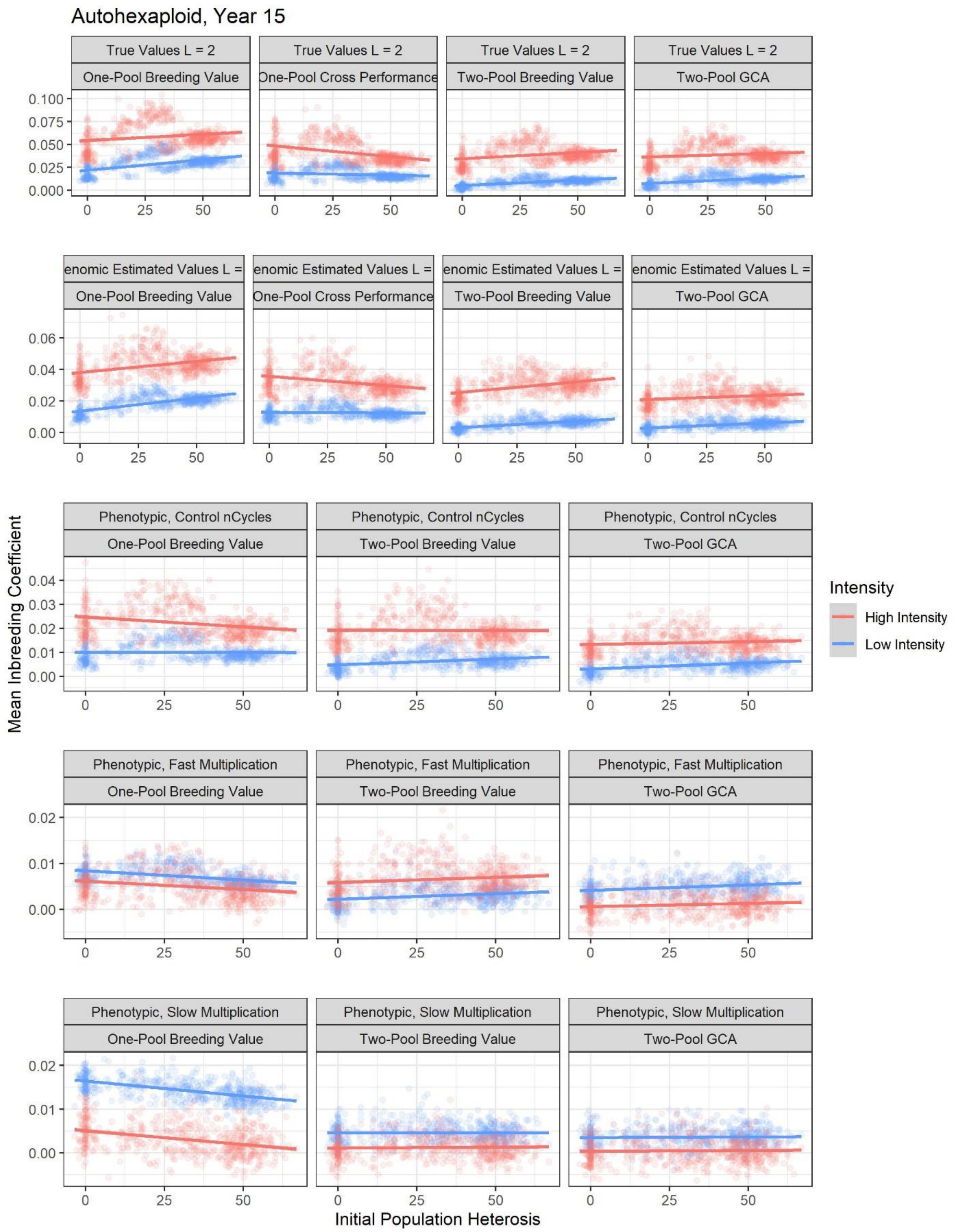

Supplemental Figure 15. Mean inbreeding coefficient in autohexaploids by estimation method, strategy, and intensity as a function of H_0 after 15 years.

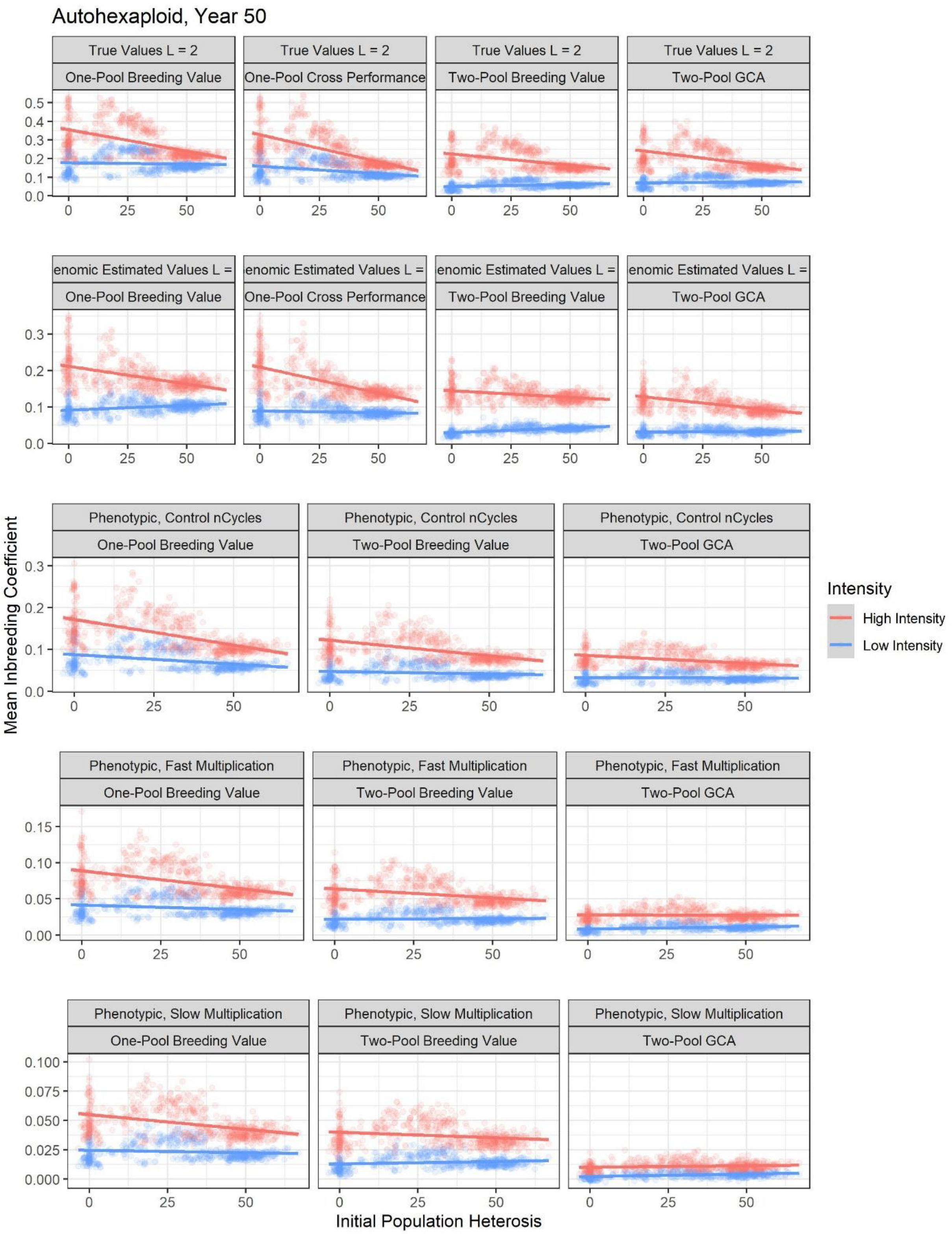

Supplemental Figure 16. Mean inbreeding coefficient in autohexaploids by estimation method, strategy, and intensity as a function of H_0 after 50 years.

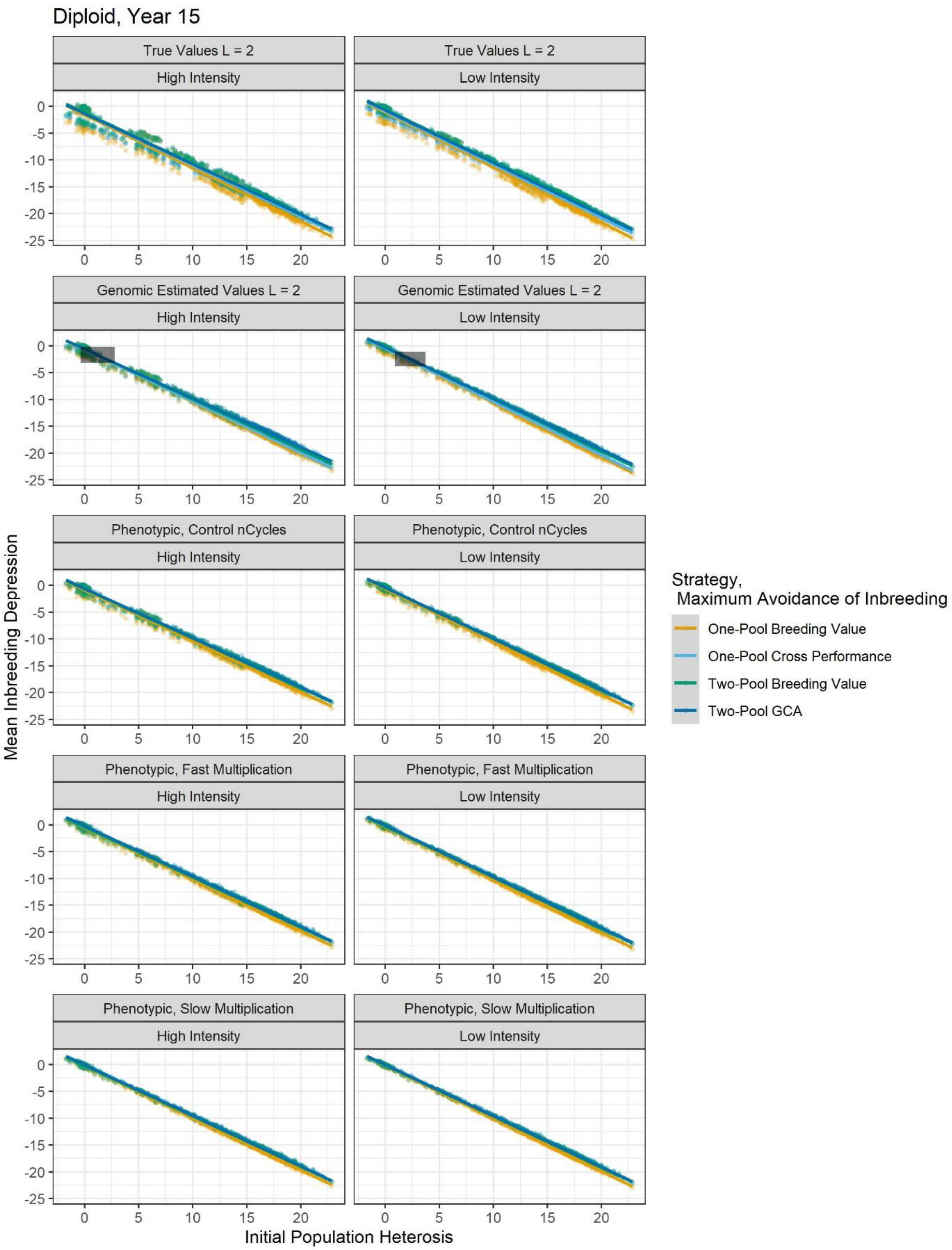

Supplemental Figure 17. Mean diploid population inbreeding depression at year 15 as a function of H_0 by strategy. Shaded boxes indicate the standard error of the intersections of the strategy regressions, if estimable.

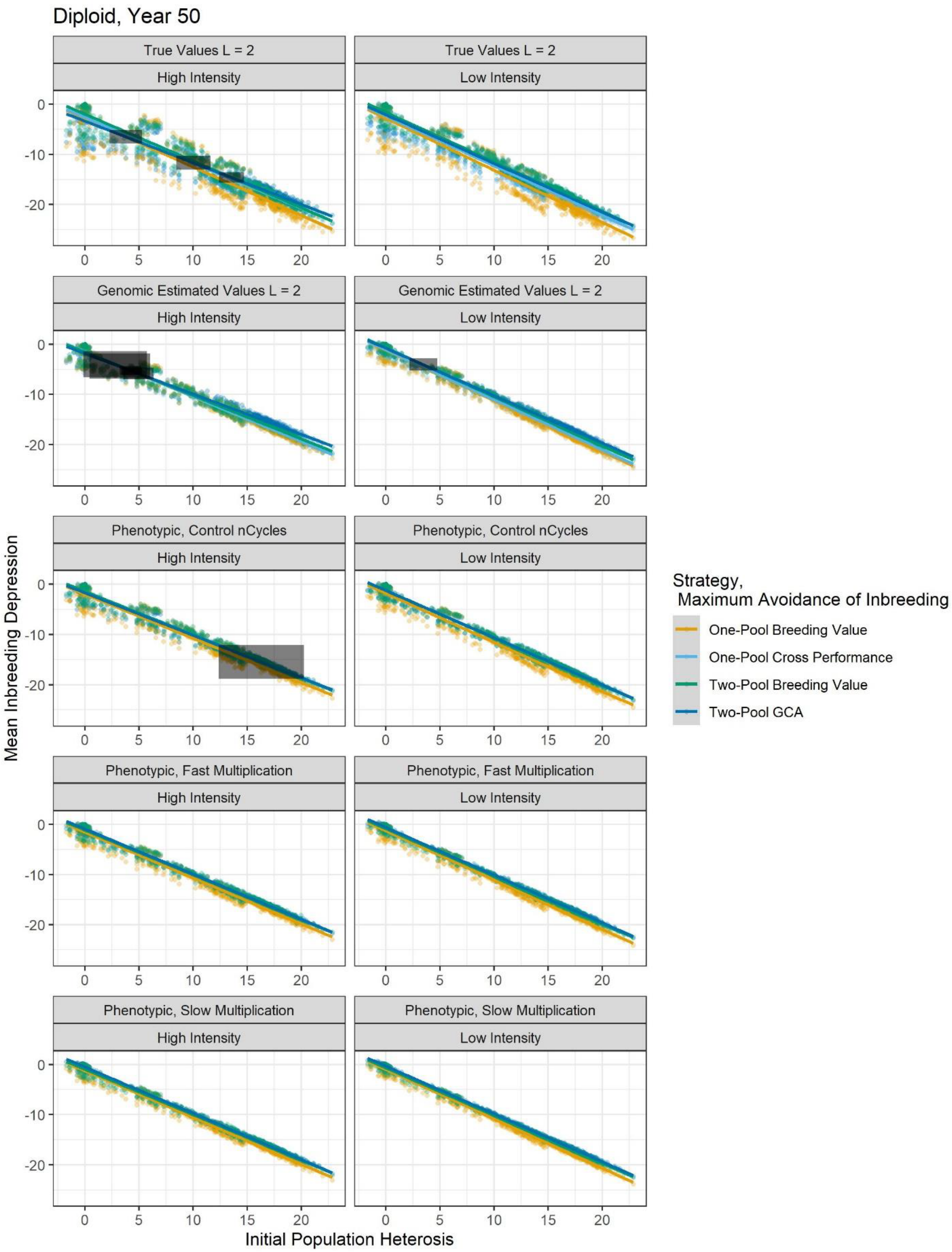

Supplemental Figure 18. Mean diploid population inbreeding depression at year 50 as a function of H_0 by strategy. Shaded boxes indicate the standard error of the intersections of the strategy regressions, if estimable.

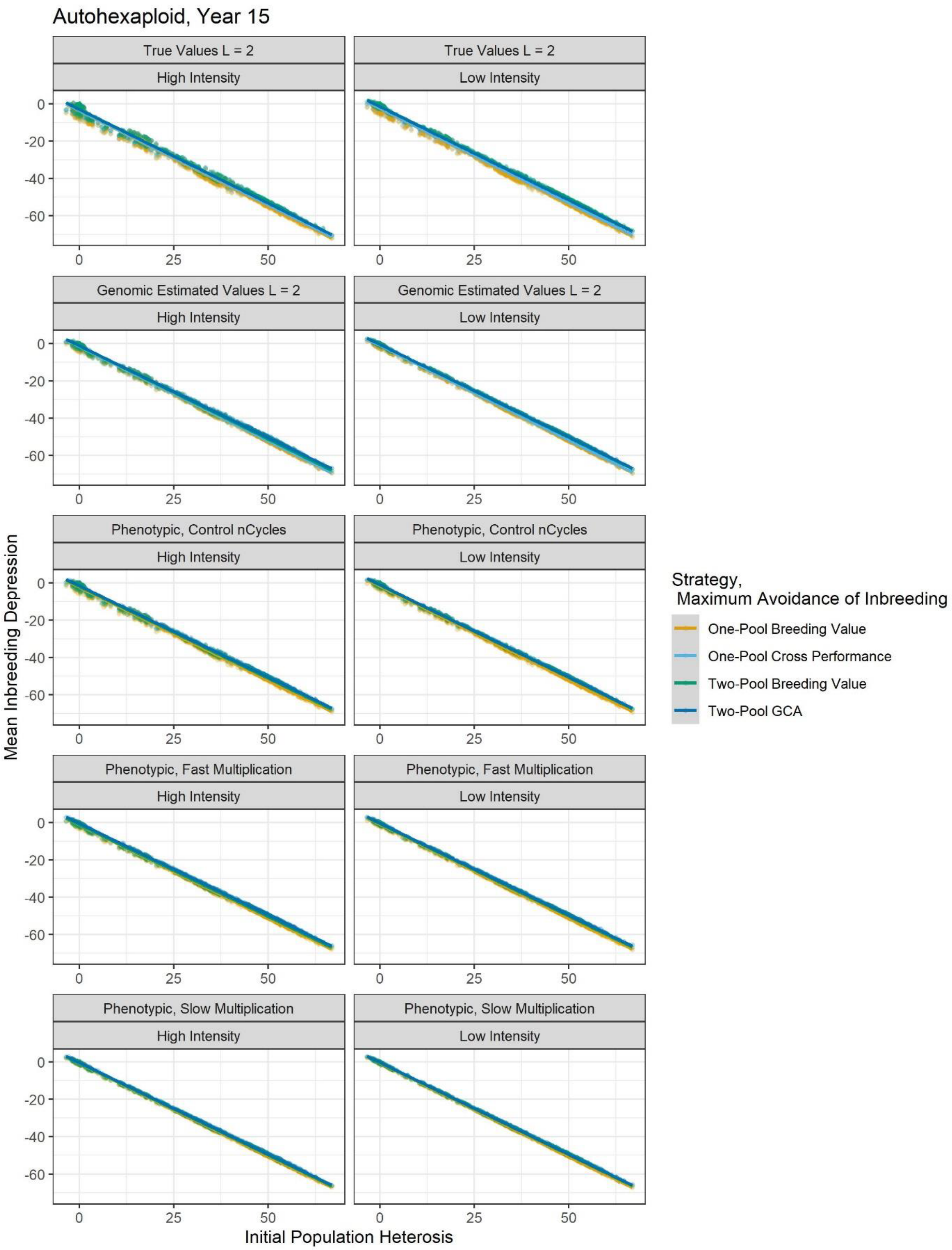

Supplemental Figure 19. Mean autohexaploid population inbreeding depression at year 15 as a function of H_0 by strategy. Shaded boxes indicate the standard error of the intersections of the strategy regressions, if estimable.

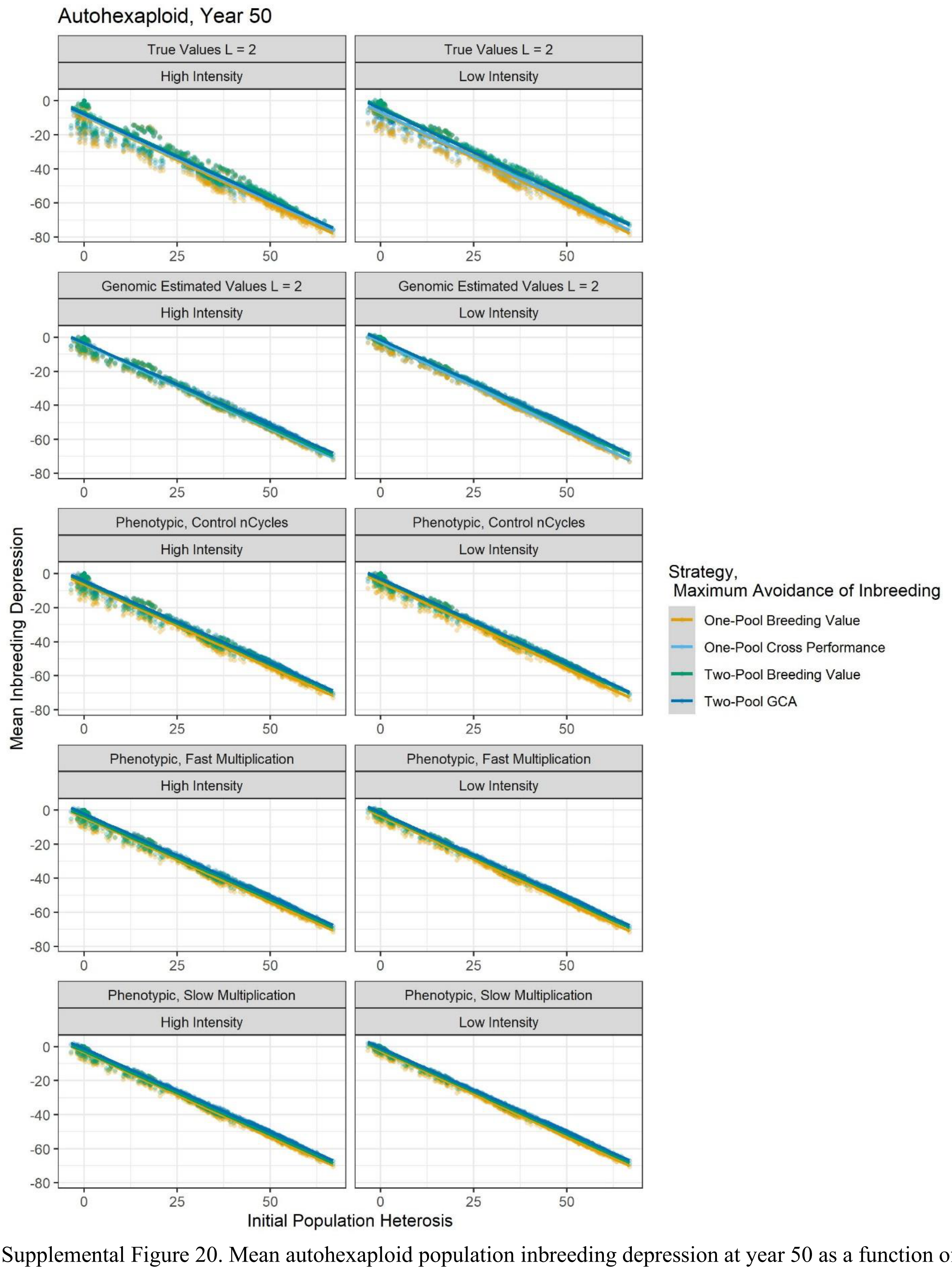

Supplemental Figure 20. Mean autohexaploid population inbreeding depression at year 50 as a function of H_0 by strategy. Shaded boxes indicate the standard error of the intersections of the strategy regressions, if estimable.

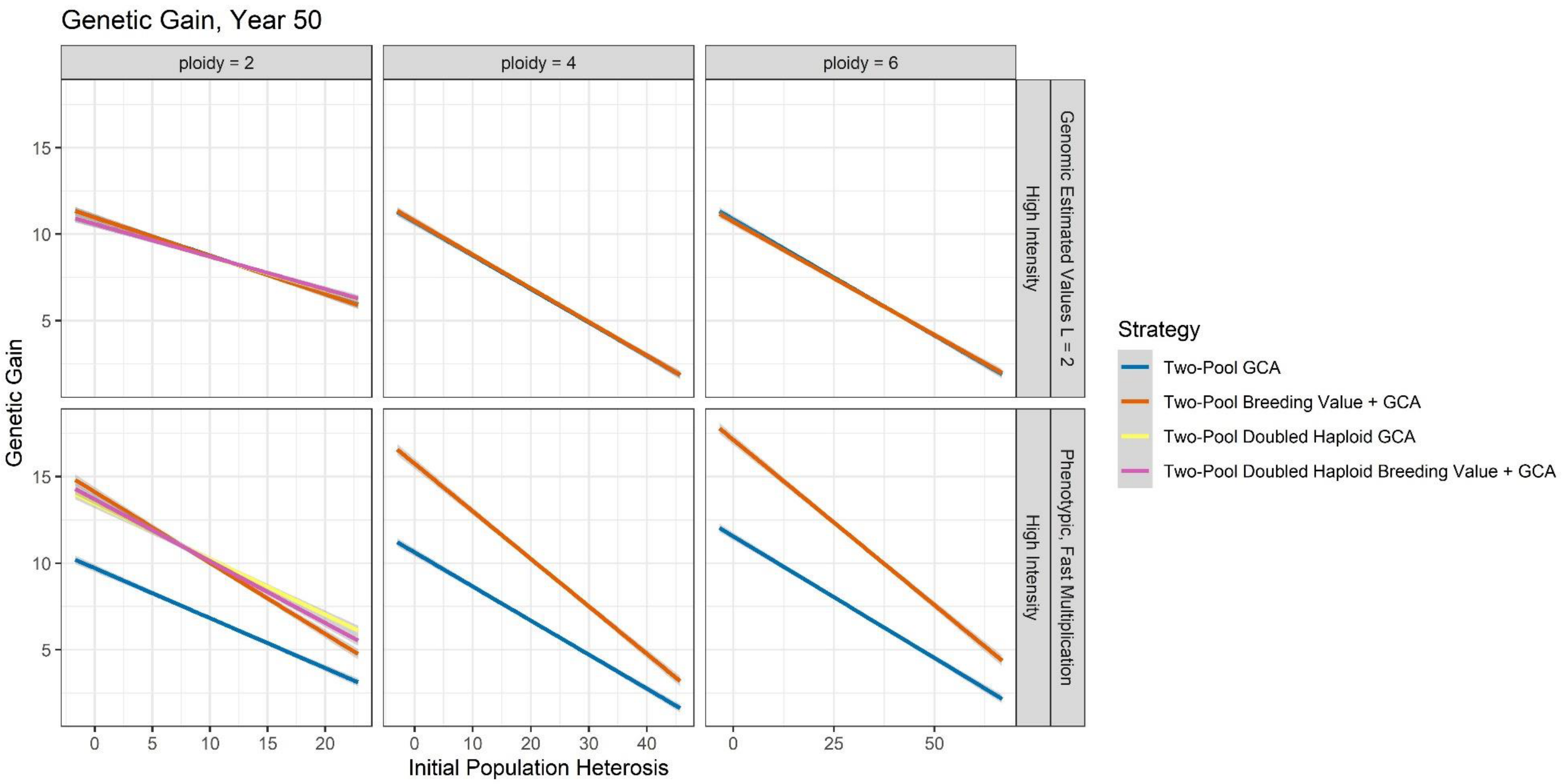

Supplemental Figure 21. Genetic gain in the non-core Breeding Value + GCA strategies as a function of H_0 after 50 years at high intensity with GS and PS. In the top row panels, the red Two-Pool Breeding Value + GCA line covers the dark blue Two-Pool GCA line. In the top left panel, the pink Two-Pool Doubled Haploid Breeding Value + GCA line covers the Two-Pool Doubled Haploid GCA line. Strategies which used doubled haploids were simulated for diploids only.

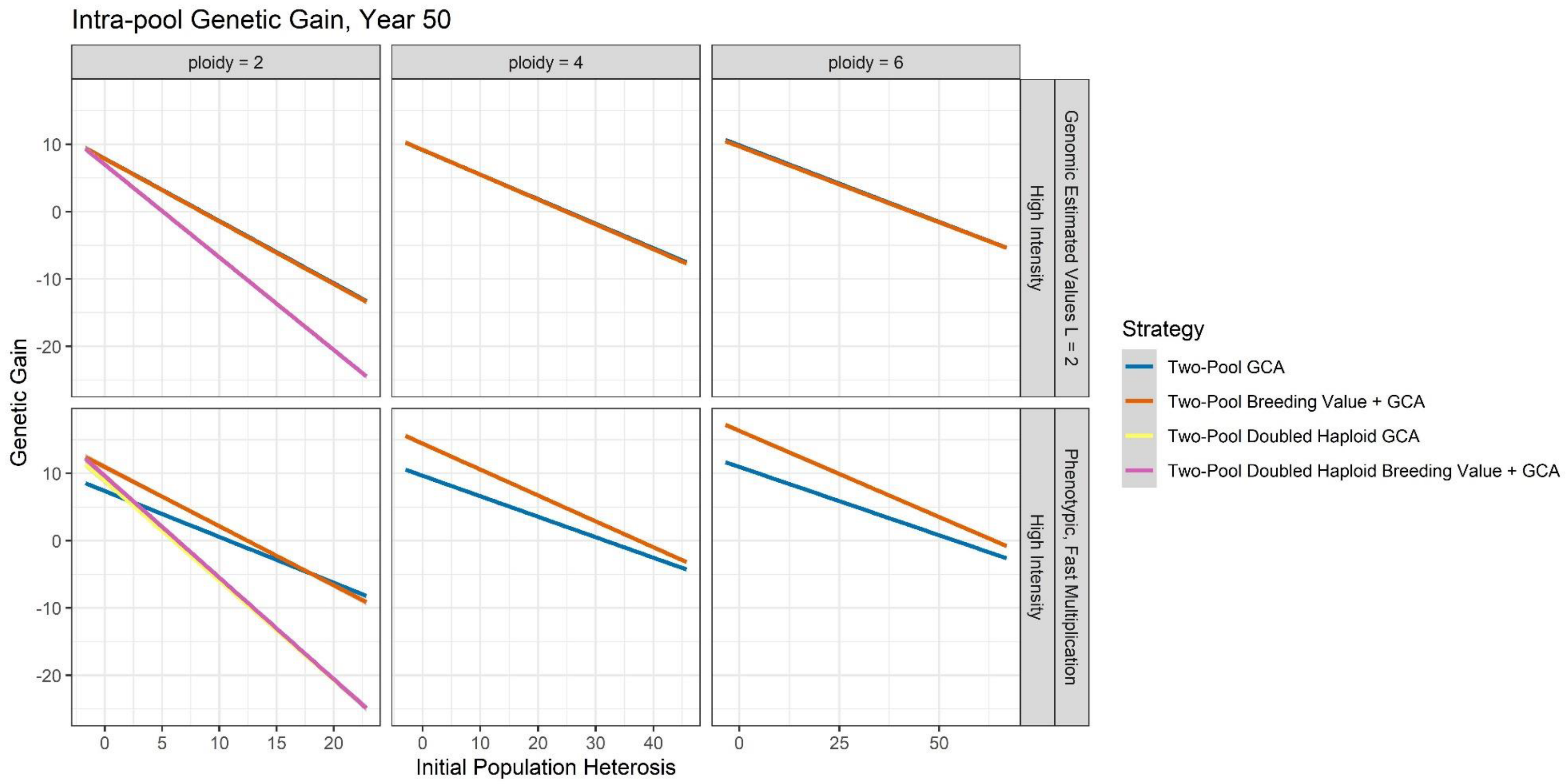

Supplemental Figure 22. Genetic gain in the intra-pool genotypes of Breeding Value + GCA strategies as a function of H_0 after 50 years at high intensity with GS and PS. In the top three panels, the red Two-Pool Breeding Value + GCA line covers the dark blue Two-Pool GCA line. In the bottom left panel, the pink Two-Pool Doubled Haploid Breeding Value + GCA line mostly covers the yellow Two-Pool Doubled Haploid GCA line. Strategies which used doubled haploids were simulated for diploids only.

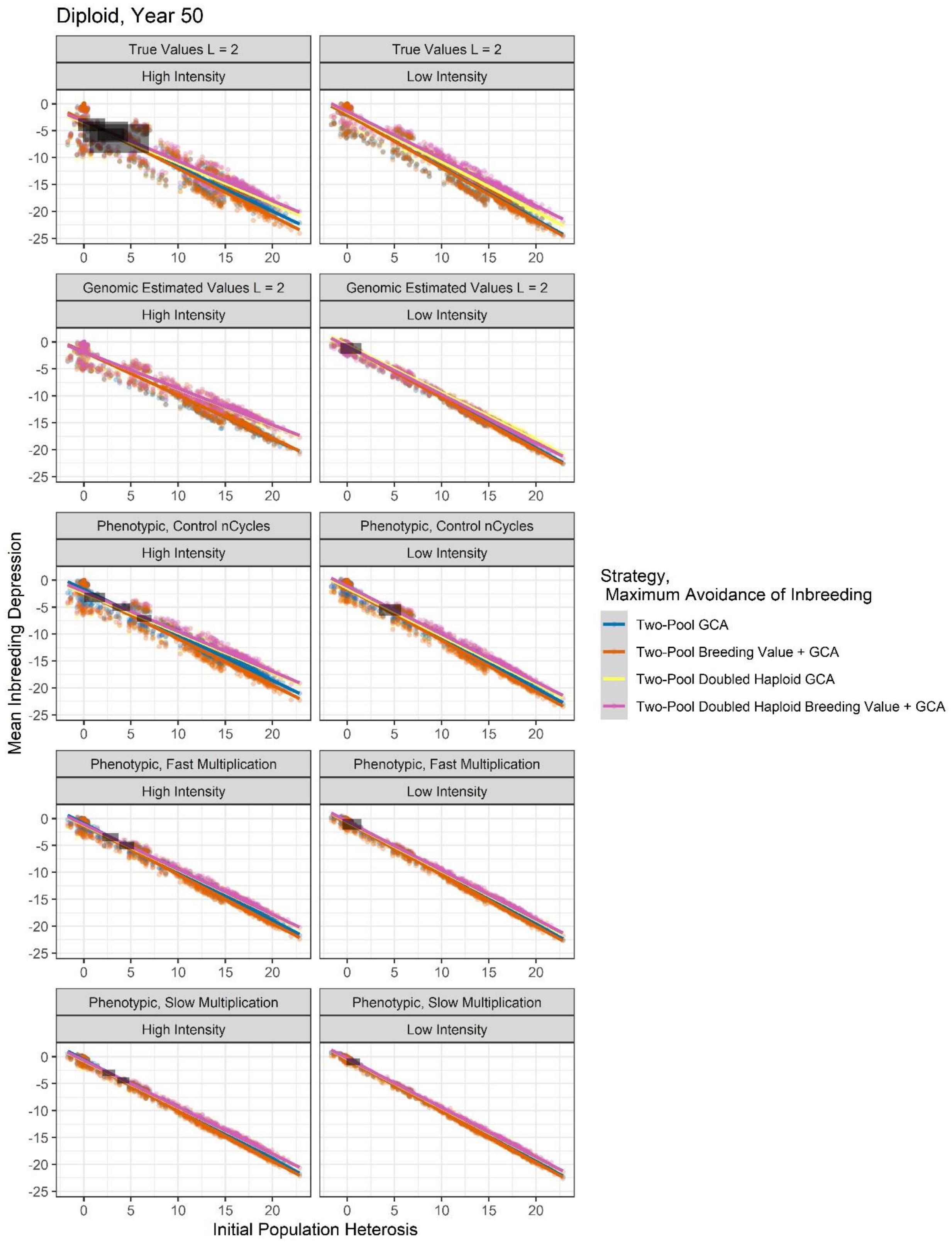

Supplemental Figure 23. Population inbreeding depression after 50 years with the non-core breeding strategies in diploids. Shaded boxes indicate the standard error of the intersections of the strategy regressions, if estimable.

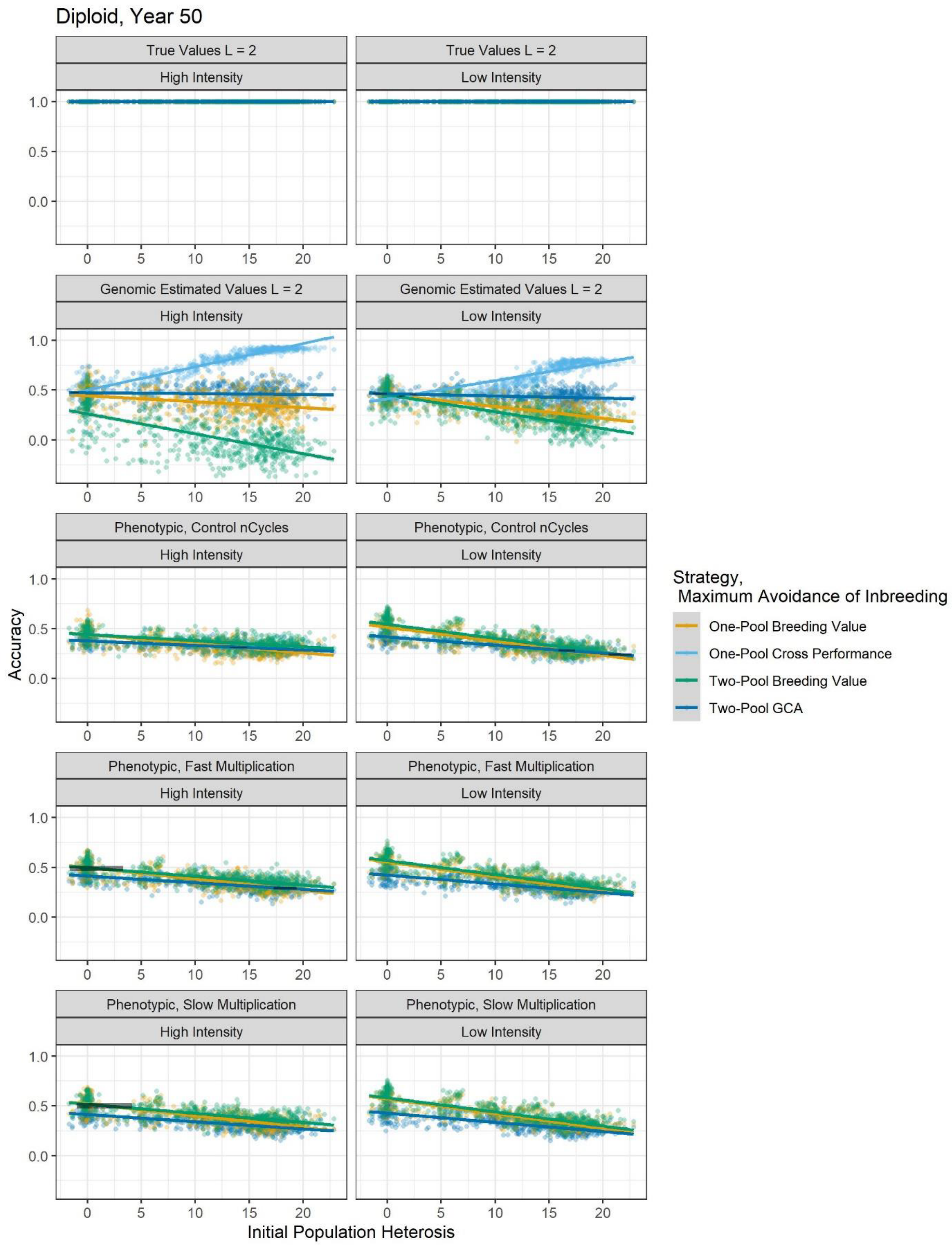

Supplemental Figure 24. Accuracy after 50 years with the core breeding strategies in diploids. Shaded boxes indicate the standard error of the intersections of the strategy regressions, if estimable.

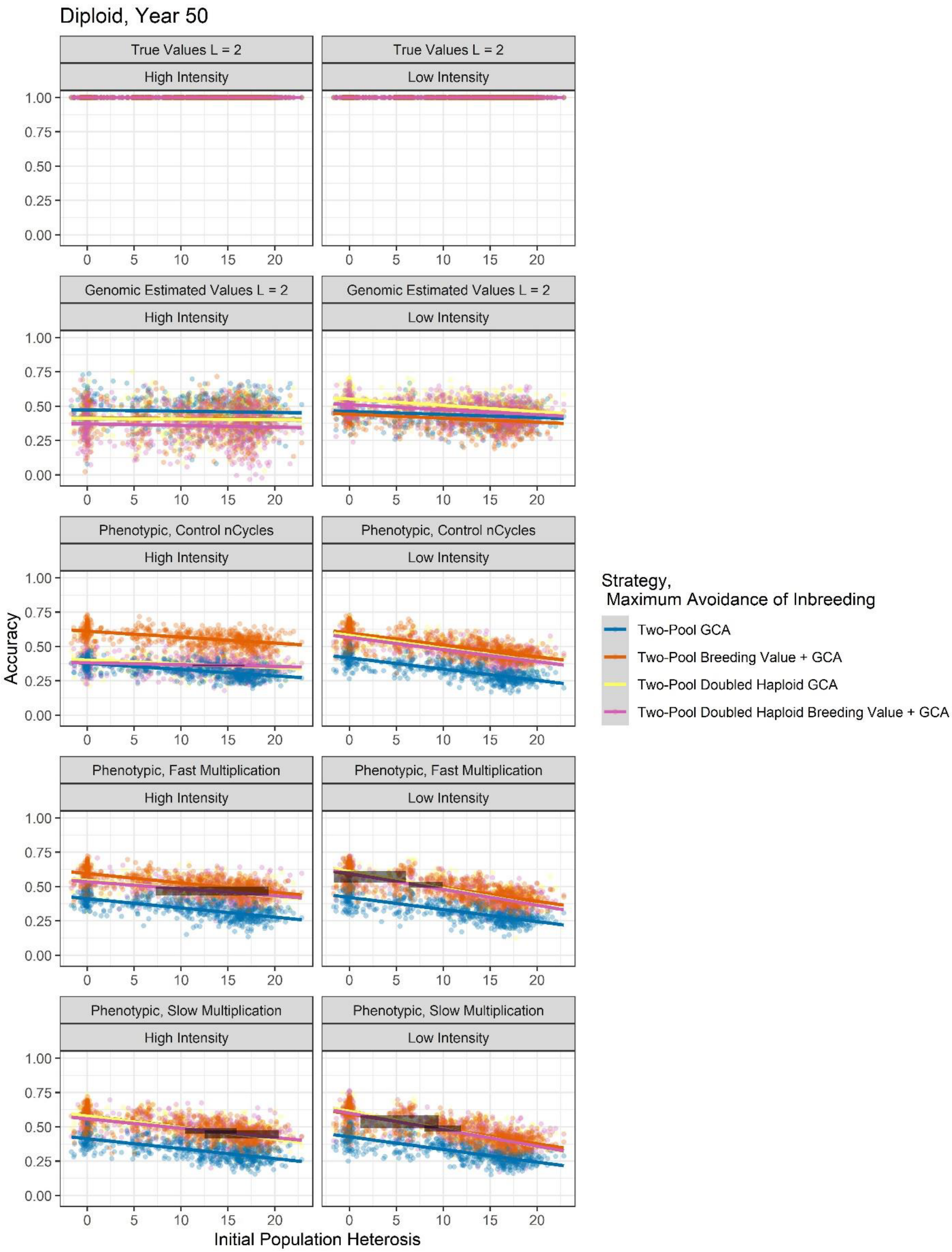

Supplemental Figure 25. Accuracy after 50 years with the non-core strategies in diploids. Shaded boxes indicate the standard error of the intersections of the strategy regressions, if estimable.

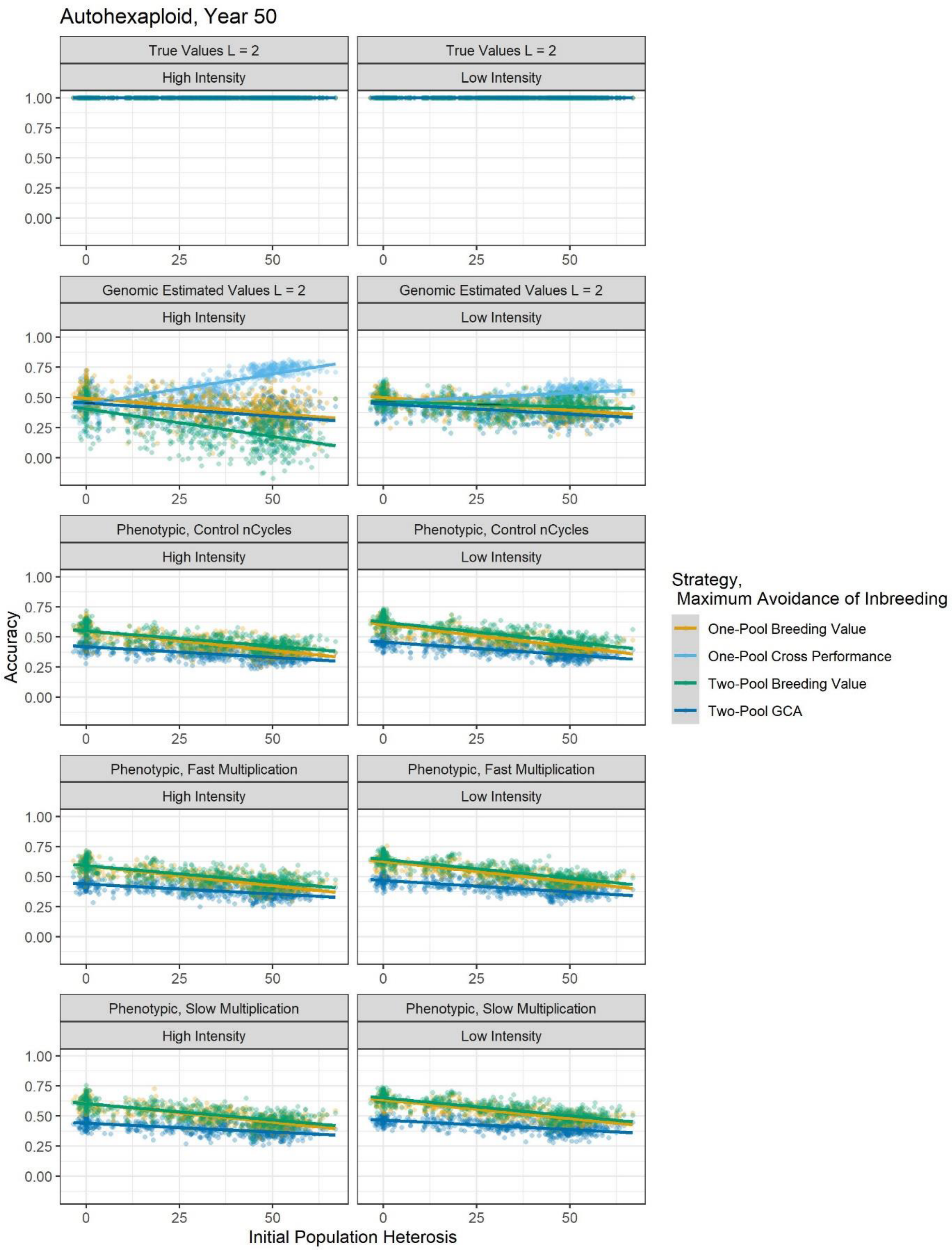

Supplemental Figure 26. Accuracy after 50 years with the core breeding strategies in autohexaploids.

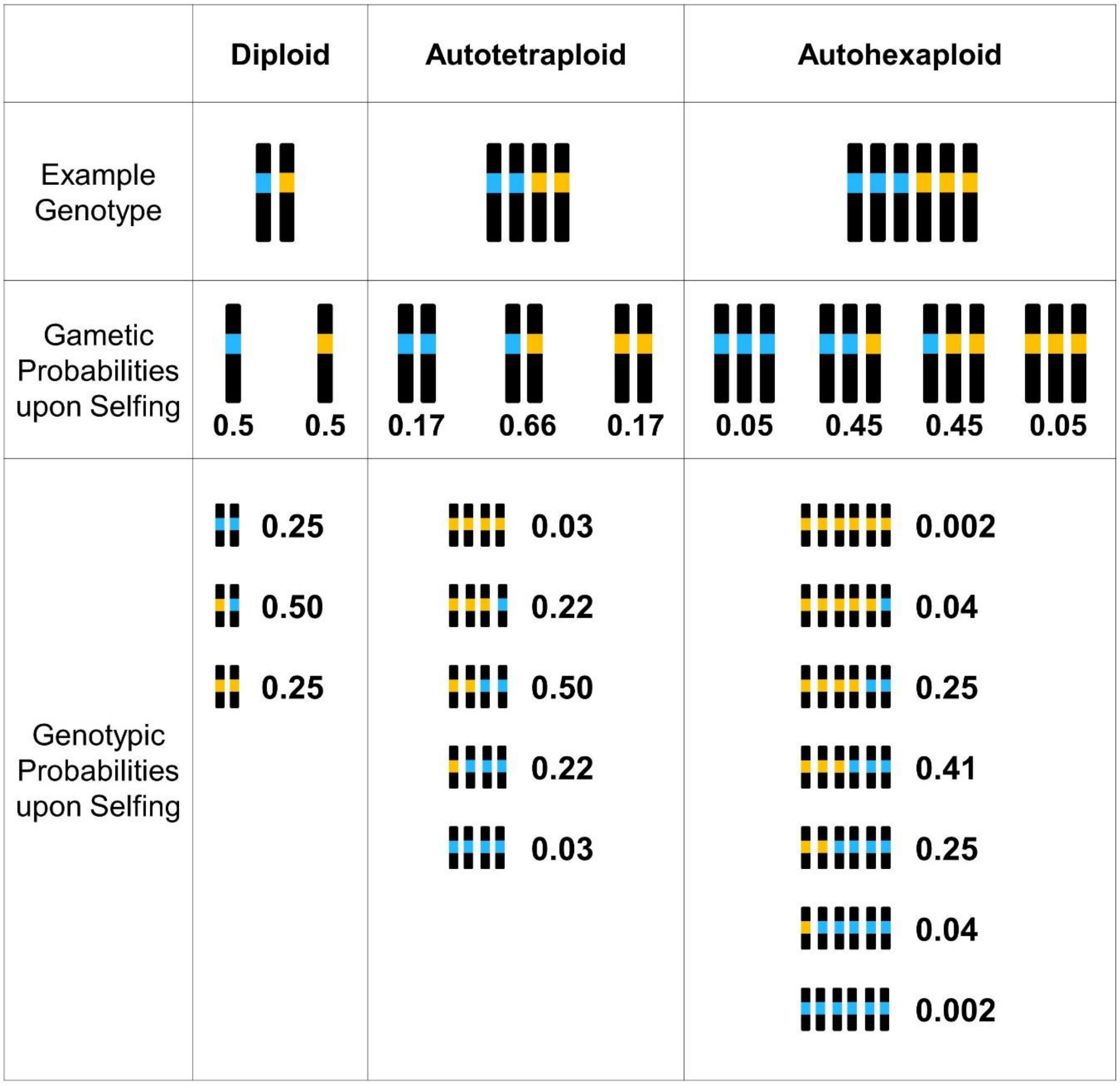

Supplemental Figure 27. Illustration of how autopolyploid meiosis leads to decreased probability of fully homozygous genotypes compared to diploids. Example genotypes are indicated by colored chromosome sets, with blue and orange rectangles indicating reference and alternate alleles. As an example, gametic and genotypic probabilities upon selfing are given in chromosome subtext. This decreased homozygosity at the gametic level leads to decreased homozygosity at the population level, as seen in Supplemental Table 3. Even though this decreased homozygosity implies that autopolyploids have a slower inbreeding rate than diploids, it does not imply that autopolyploids suffer less inbreeding depression than diploids.

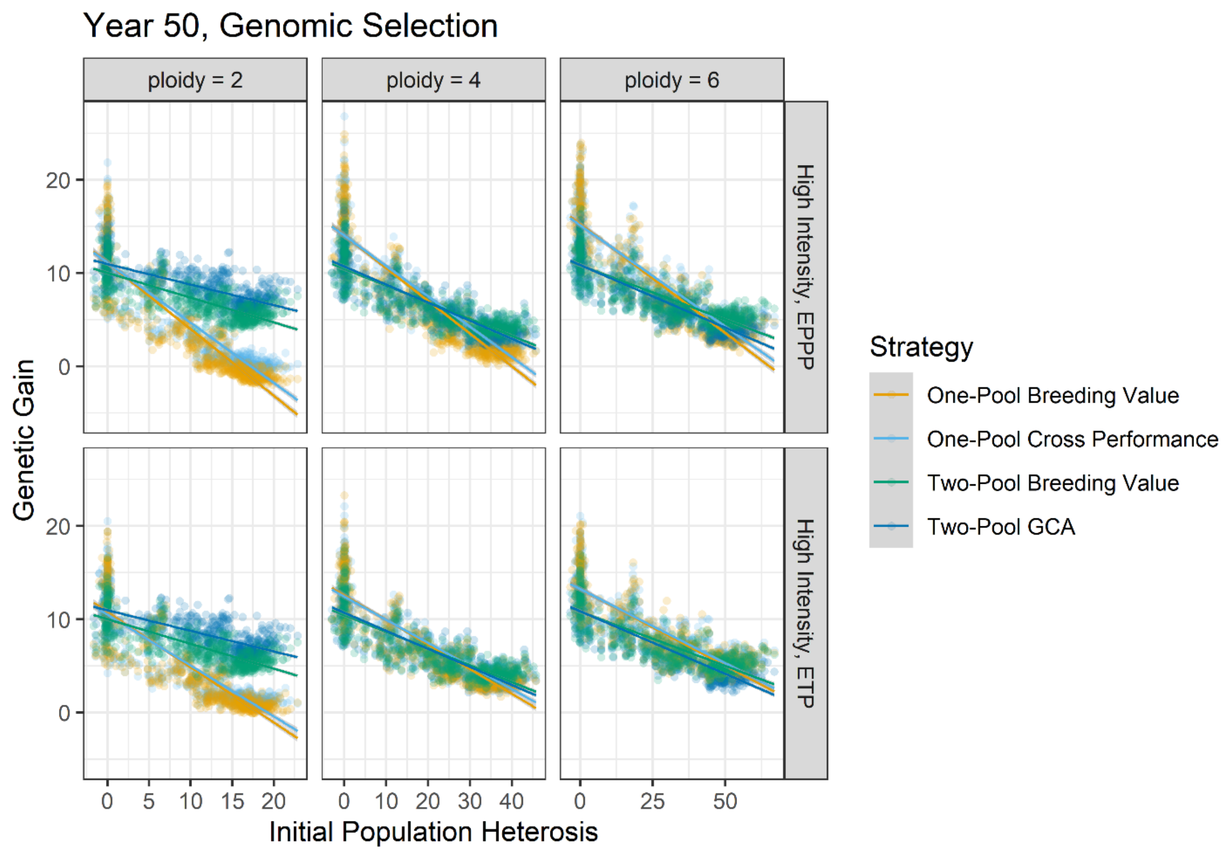

Supplemental Figure 28. Genetic gain after 50 years in the core strategies with use of genomic estimated values at high intensity and equal parents per pool (EPPP) vs. equal total parents (ETP). EPPP was achieved by reducing the number of parents in the one-pool ETP scenarios to match the two-pool scenarios (and increasing the progeny per cross to keep program size the same).

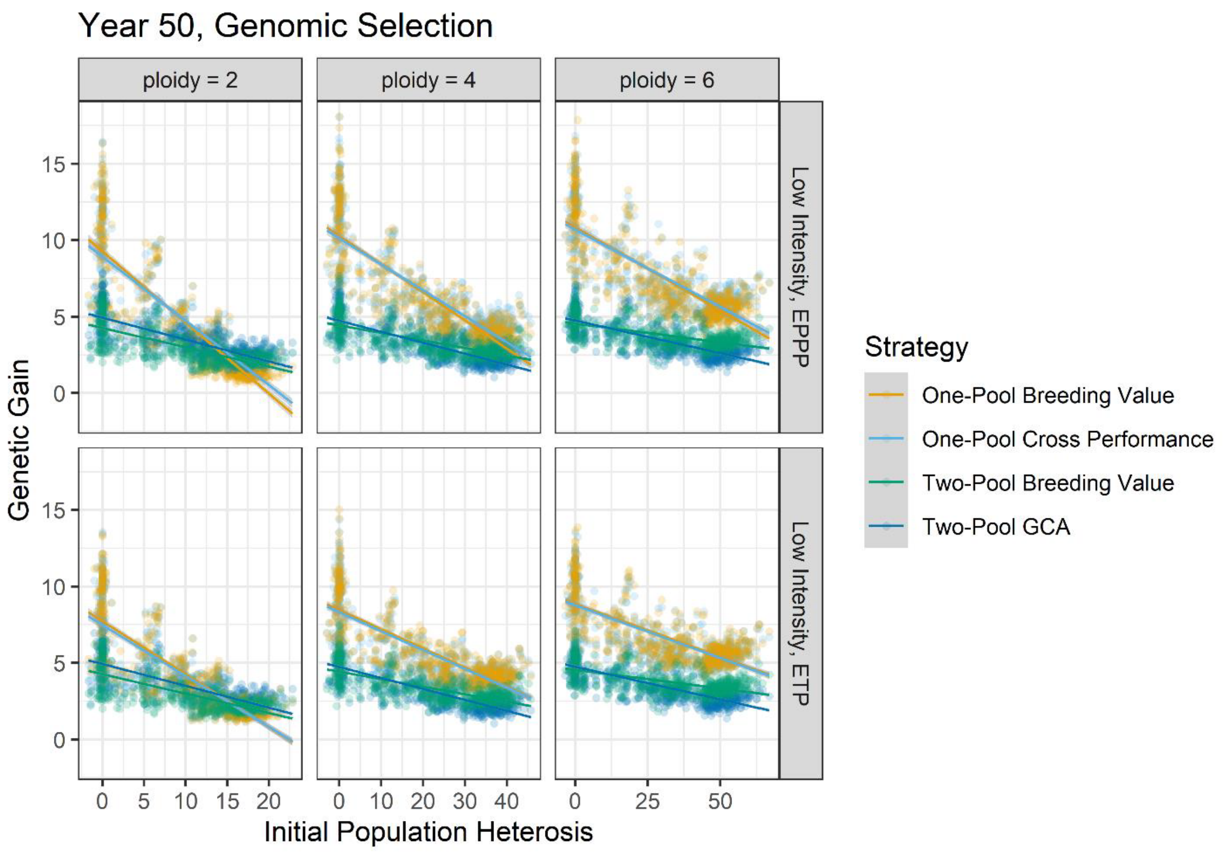

Supplemental Figure 29. Genetic gain after 50 years in the core strategies with use of genomic estimated values at low intensity and equal parents per pool (EPPP) vs. equal total parents (ETP). EPPP was achieved by reducing the number of parents in the one-pool ETP scenarios to match the two-pool scenarios (and increasing the progeny per cross to keep program size the same).

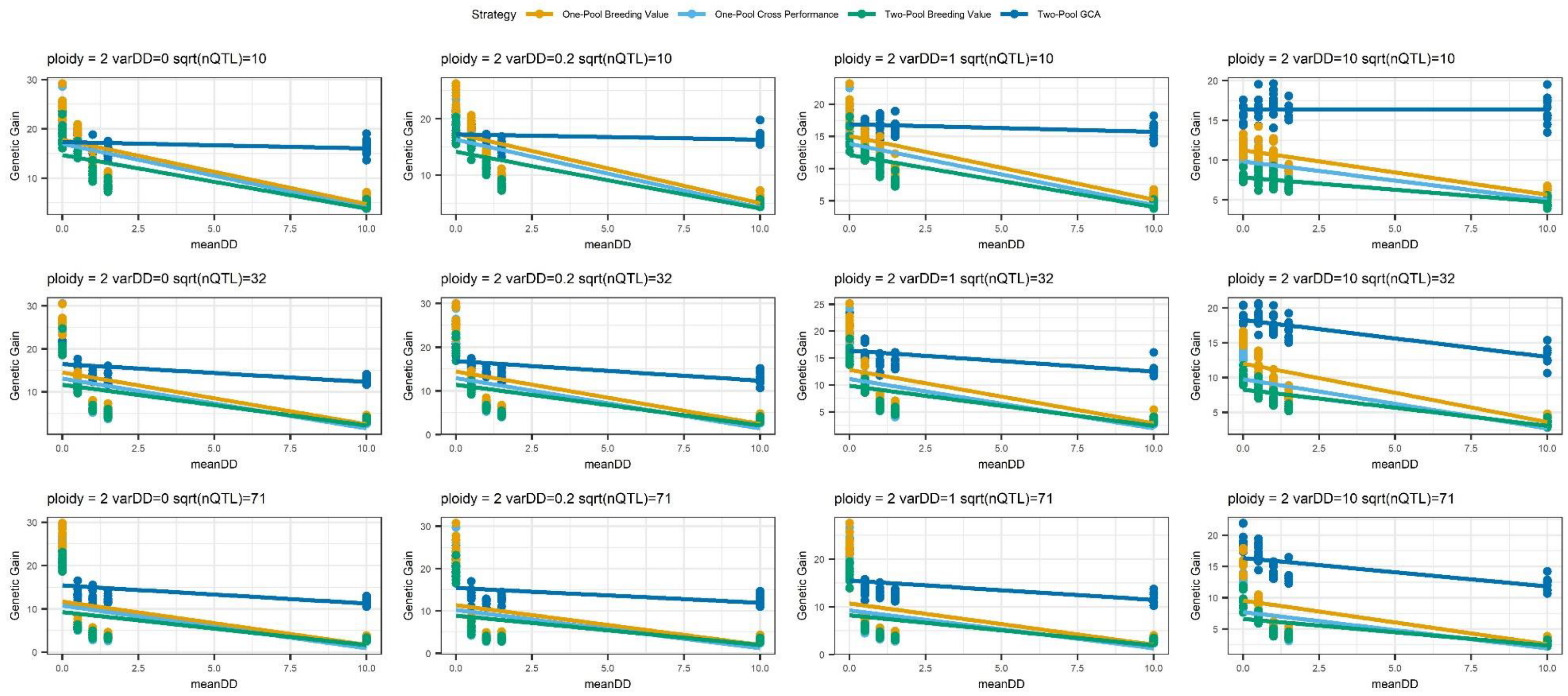

Supplemental Figure 30. For use of true values at high intensity after 50 years in diploids, the relative performance of the core strategies as a function of mean dominance degree (meanDD) instead of H_0, at each level of the variance of dominance degrees (varDD) and the square root of the number of QTL per chromosome (sqrt(nQTL)).

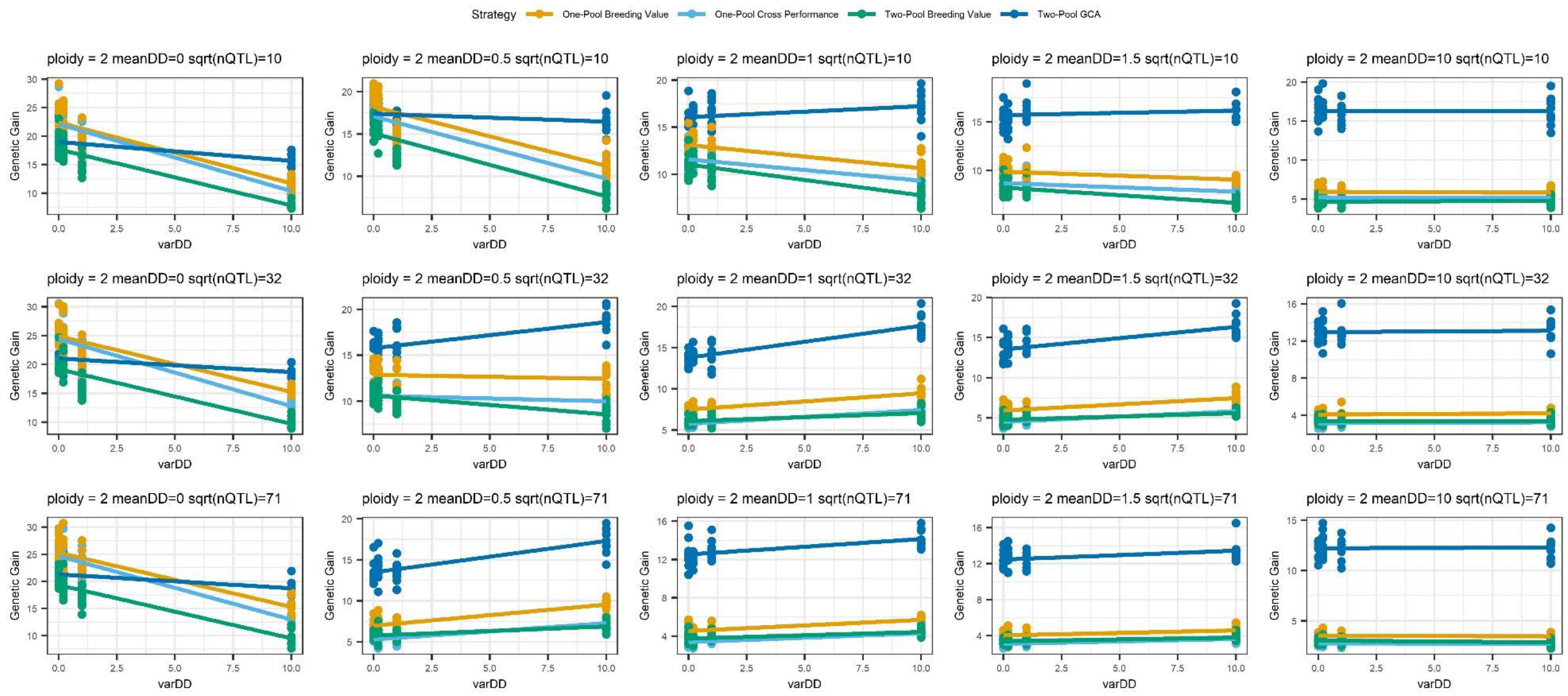

Supplemental Figure 31. For use of true values at high intensity after 50 years in diploids, the relative performance of the core strategies as a function of the variance of dominance degrees (varDD) instead of H_0, at each level of the mean dominance degree (meanDD) and the square root of the number of QTL per chromosome (sqrt(nQTL)).

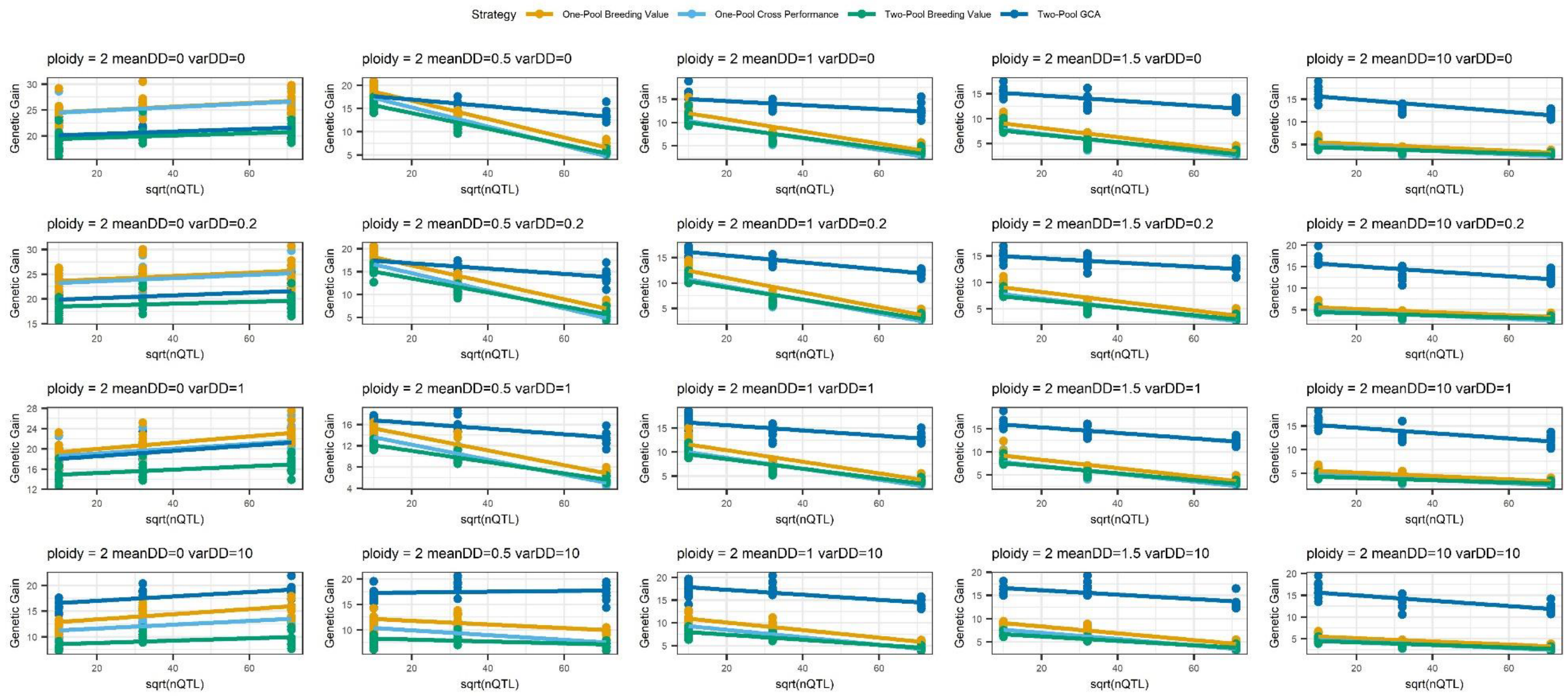

Supplemental Figure 32. For use of true values at high intensity after 50 years in diploids, the relative performance of the core strategies as a function of the square root of the number of QTL per chromosome (sqrt(nQTL) instead of H_0, at each level of the variance of dominance degrees (varDD) and the mean dominance degree (meanDD).

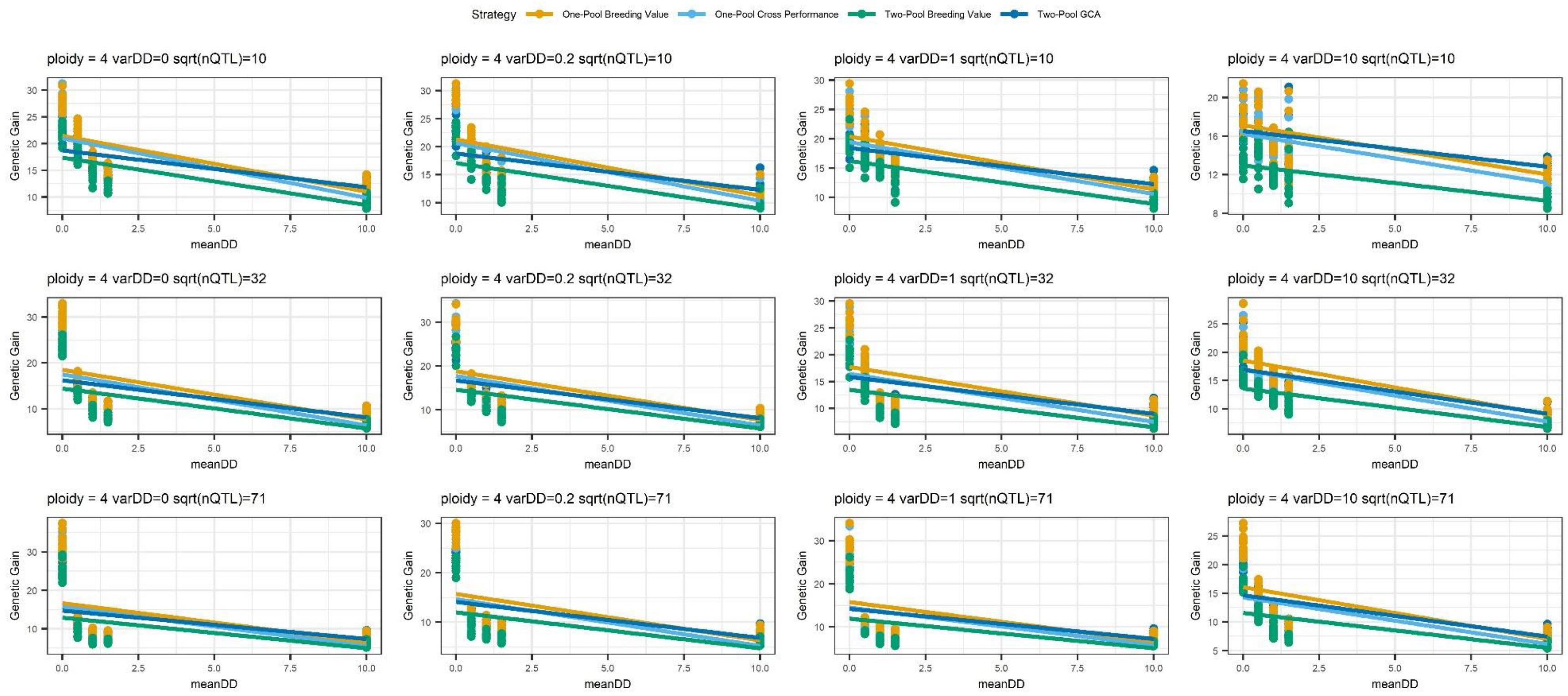

Supplemental Figure 33. For use of true values at high intensity after 50 years in autotetraploids, the relative performance of the core strategies as a function of mean dominance degree (meanDD) instead of H_0, at each level of the variance of dominance degrees (varDD) and the square root of the number of QTL per chromosome (sqrt(nQTL)).

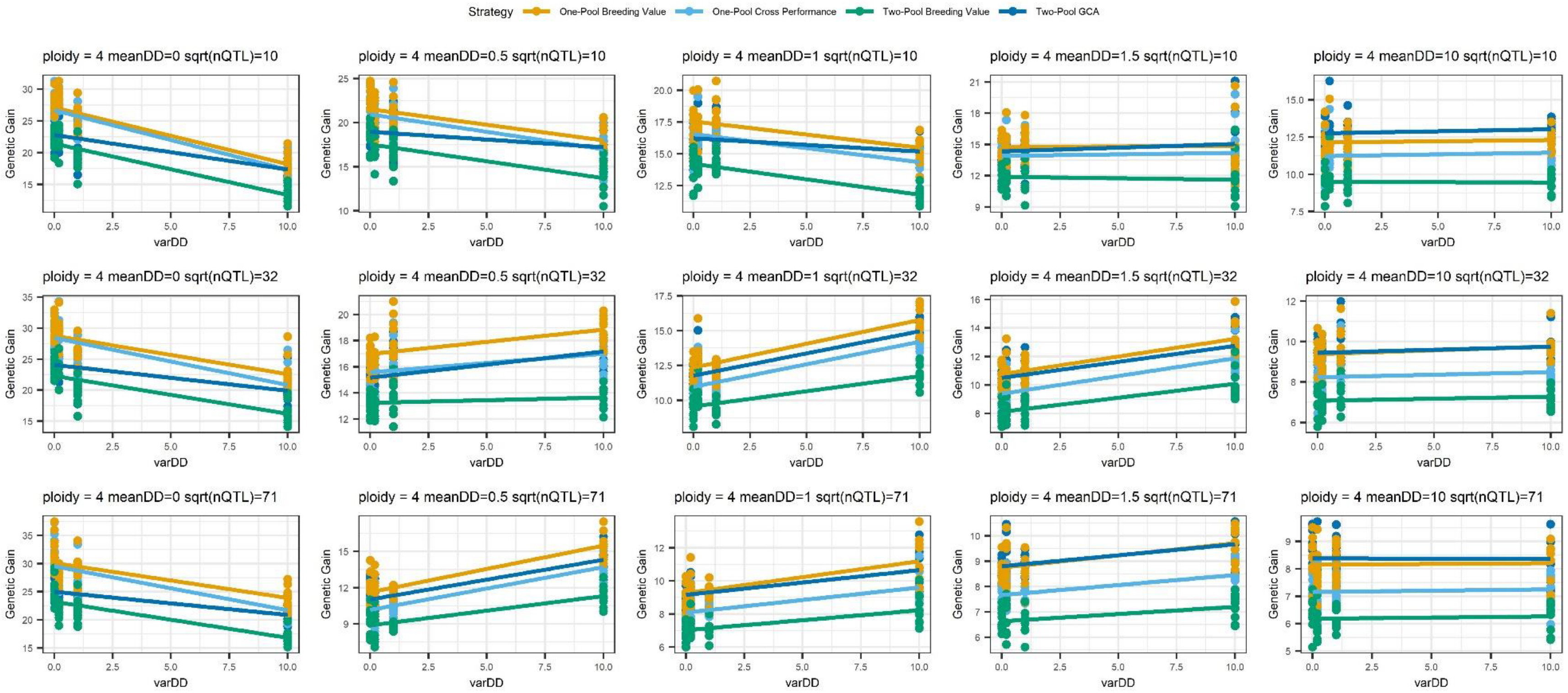

Supplemental Figure 34. For use of true values at high intensity after 50 years in autotetraploids, the relative performance of the core strategies as a function of the variance of dominance degrees (varDD) instead of H_0, at each level of the mean dominance degree (meanDD) and the square root of the number of QTL per chromosome (sqrt(nQTL)).

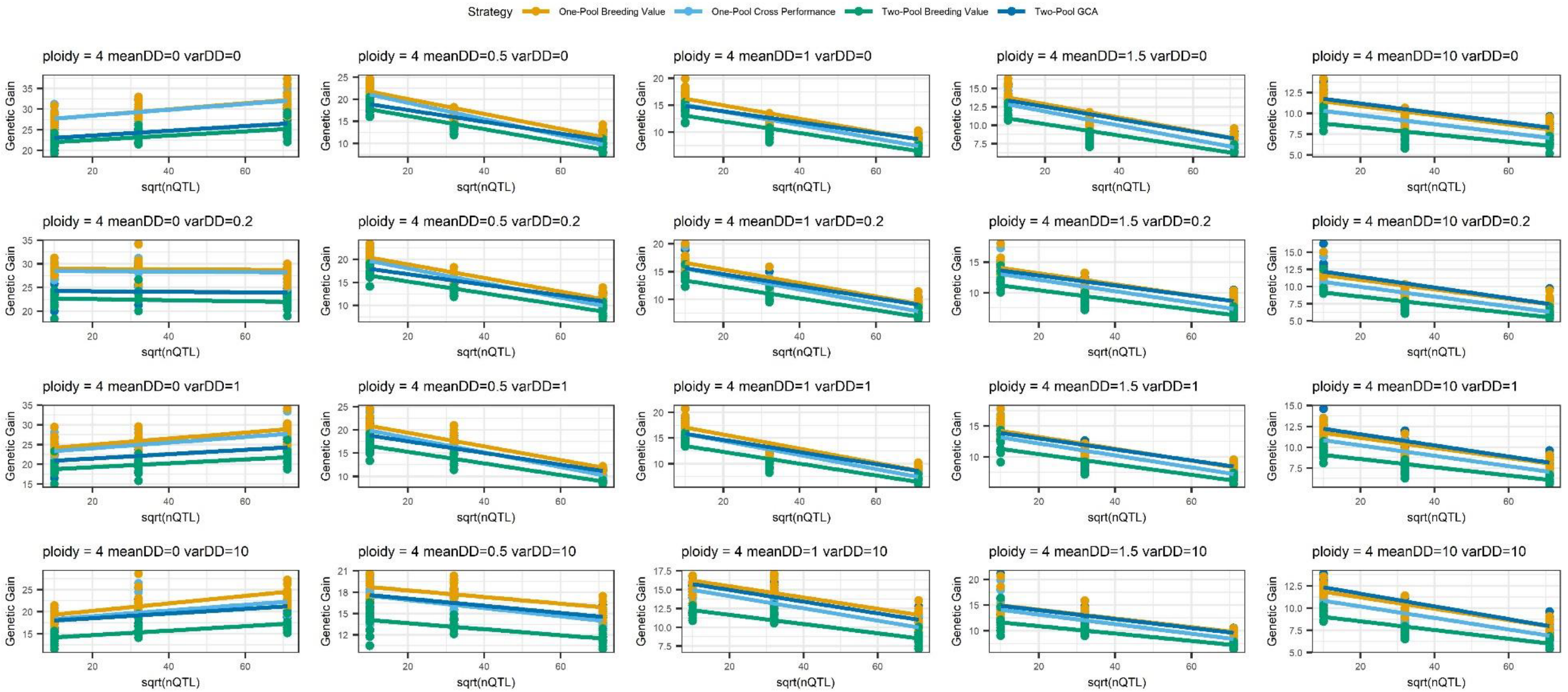

Supplemental Figure 35. For use of true values at high intensity after 50 years in autotetraploids, the relative performance of the core strategies as a function of the square root of the number of QTL per chromosome (sqrt(nQTL) instead of H_0, at each level of the variance of dominance degrees (varDD) and the mean dominance degree (meanDD).

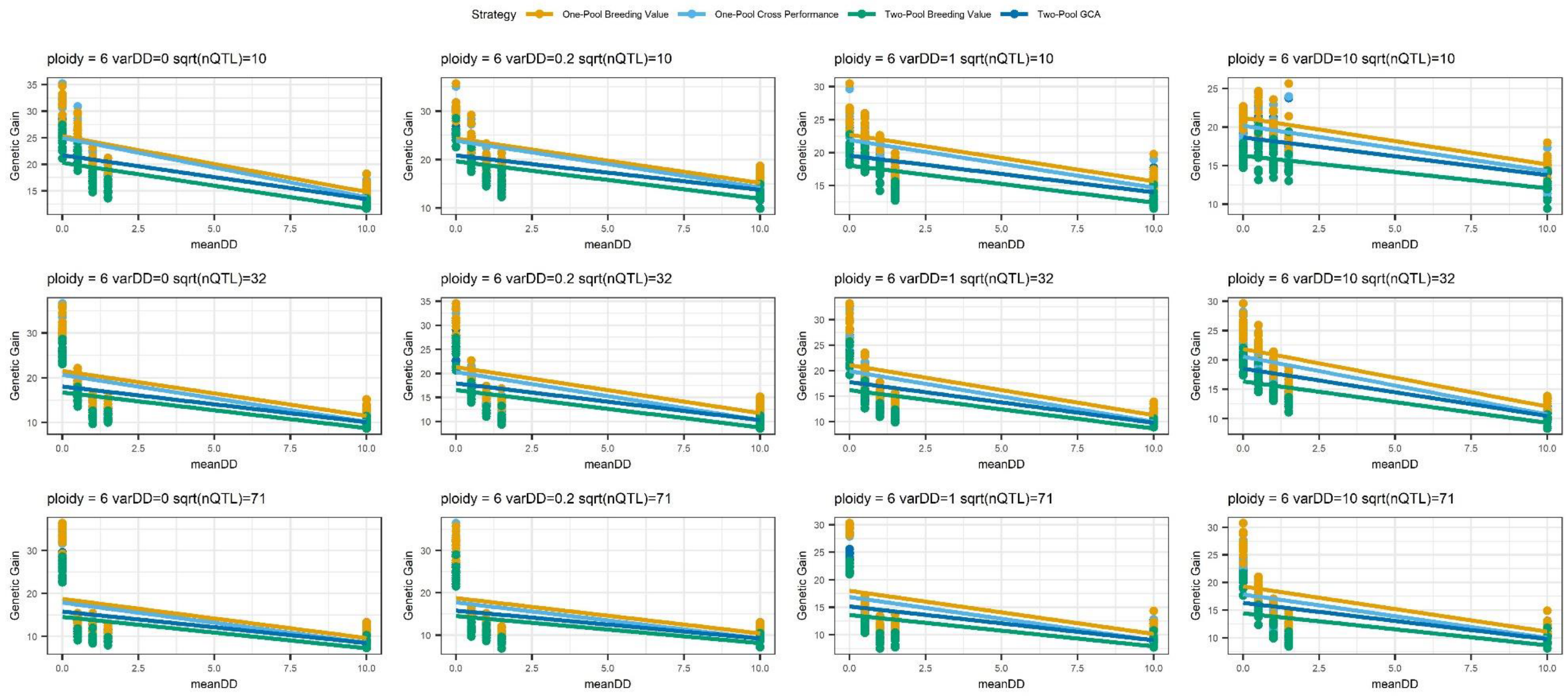

Supplemental Figure 36. For use of true values at high intensity after 50 years in autohexaploids, the relative performance of the core strategies as a function of mean dominance degree (meanDD) instead of H_0, at each level of the variance of dominance degrees (varDD) and the square root of the number of QTL per chromosome (sqrt(nQTL)).

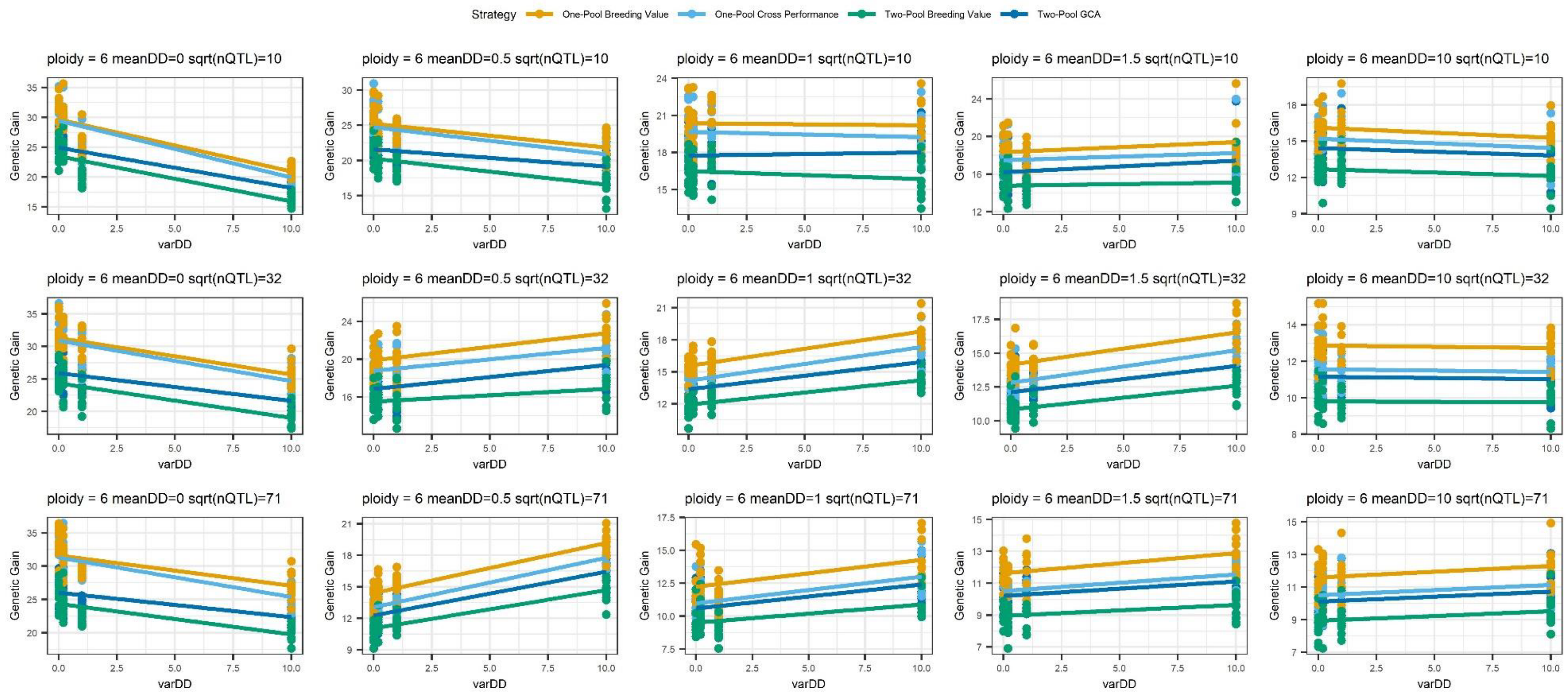

Supplemental Figure 37. For use of true values at high intensity after 50 years in autohexaploids, the relative performance of the core strategies as a function of the variance of dominance degrees (varDD) instead of H_0, at each level of the mean dominance degree (meanDD) and the square root of the number of QTL per chromosome (sqrt(nQTL)).

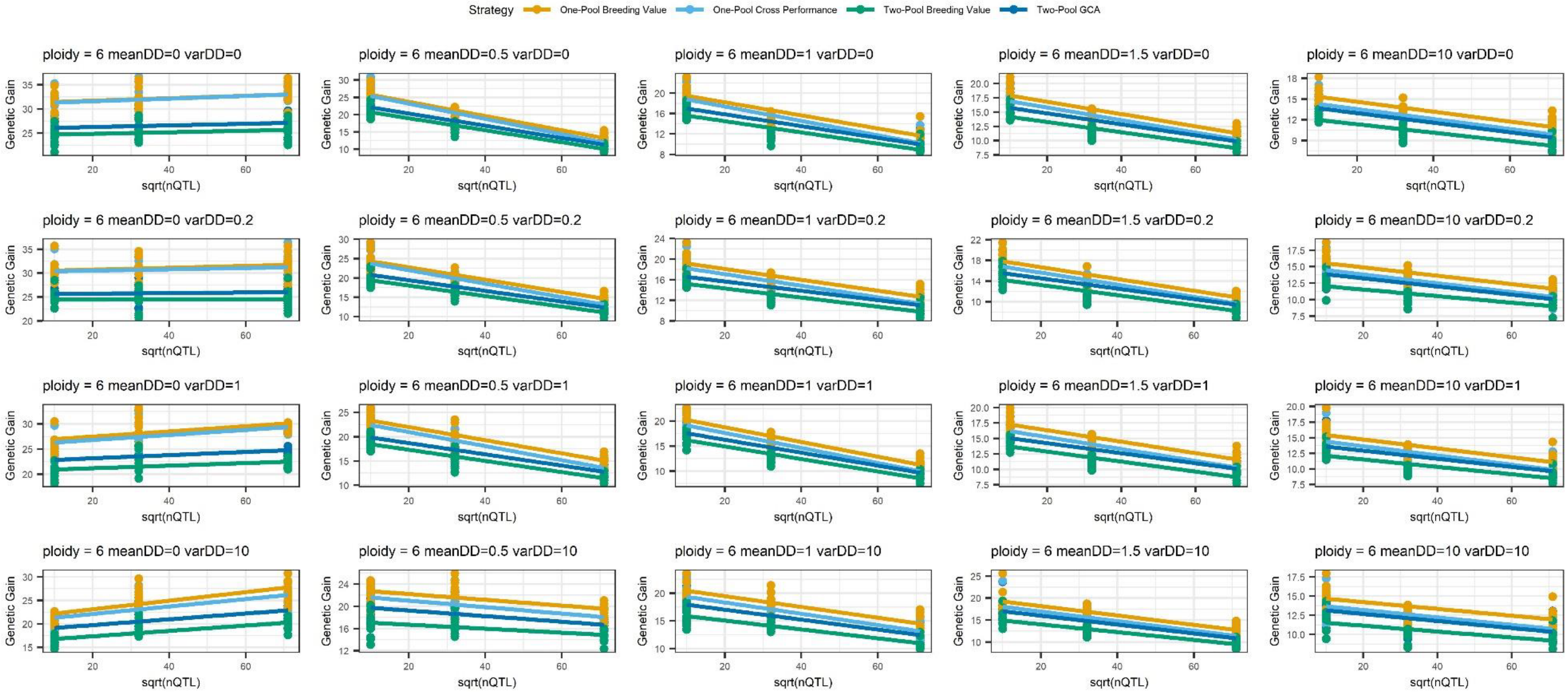

Supplemental Figure 38. For use of true values at high intensity after 50 years in autohexaploids, the relative performance of the core strategies as a function of the square root of the number of QTL per chromosome (sqrt(nQTL) instead of H_0, at each level of the variance of dominance degrees (varDD) and the mean dominance degree (meanDD).
