# Supplementary material for: Clonal diploid and autopolyploid breeding strategies to harness heterosis: insights from stochastic simulation
Source: Theor Appl Genet. 2023 Jun 8;136(7):147. doi: 10.1007/s00122-023-04377-z (PMC10250475; doi:10.1007/s00122-023-04377-z)
Supplement: Supplementary file 2 — Supplementary file2 (PDF 168 kb) [file 122_2023_4377_MOESM2_ESM.pdf]

Clonal diploid and autopolyploid breeding strategies to harness heterosis: insights from stochastic simulation. Theoretical and Applied Genetics. Marlee R. Labroo, Jeffrey B. Endelman, Dorcus C. Gemenet, Christian R. Werner, R. Chris Gaynor, Giovanny Eduardo Covarrubias-Pazaran (Excellence in Breeding Platform, Consultative Group of International Agricultural Research; covaruberpaz@gmail.com)

Simulation of true additive and dominance genetic values

The QTL total additive effects *A(x)* are determined such that

$$A(x) = \sum ax_A$$

where *a* is the additive effect of a given QTL. The additive effects are sampled from a normal distribution and subsequently scaled to achieve the requested total additive variance (here 1) by multiplying the sampled additive effect by a constant equal to the initial additive standard deviation divided by the targeted additive standard deviation. The term x_A is the scaled additive genotype dosage of the individual at the QTL, such that:

$$x_A = (x - \frac{\phi}{2})(\frac{2}{\phi})$$

where ϕ is the individual's ploidy level.

The QTL total dominance effects *D(x)* are determined such that

$$D(x) = \sum dx_D$$

Where *d* is the dominance effect of a given QTL. The dominance effect is the product of the dominance degree and the absolute value of the QTL additive effect, where the dominance degree is sampled from a normal distribution with a mean of the appropriate mean dominance degree (meanDD) and a variance of the appropriate variance of dominance degrees (varDD). The dominance effects were concurrently scaled with additive effects to achieve the targeted additive genetic variance in this study. The term x_D is the scaled dominance genotype dosage of the individual at the QTL, such that:

$$x_D = x(\phi - x)(\frac{2}{\phi})^2$$

Breeding Value

To calculate true breeding value, the following procedure was used. If true breeding value was calculated, the genotypic values G_{jk} of each true QTL were calculated as $G_{jk} = x_{A_k}a_j + x_{D_k}d_j$, where x_{A_k} is the kth element in a vector of each *x* true QTL scaled additive genotype dosages, a_j was the true additive effect of the QTL, x_{D_k} is the kth element in a vector of each *x* true QTL scaled dominance genotype dosages, and d_j was the true dominance effect of the QTL. The locus mean genotype dosage L_j was calculated as $L_j = \sum_k^n x_k\, l_{jk}$, where x_k is the kth element in a vector *x* = {0… ϕ} of true QTL raw genotype dosages and l_{jk} is the population frequency of genotype x_k at locus *j*. The true population mean genetic value at the locus $\bar{G}_j$ was $\bar{G}_j = \sum_k^n G_{jk} * l_{jk}$. Then, the breeding value at the locus was the regression coefficient of total genotypic value on allele dosage (Falconer, 1985), so $\alpha_j = \frac{\sum_k^n l_{jk}*(G_{jk}-\bar{G}_j)*(x_k - L_j)}{\sum_j^k l_{jk}*(x_k - L_j)^2}$. The individual true breeding value was the sum of the breeding values over all *j* true QTL loci.

To estimate breeding value using the RRBLUP_D model, the procedure was generally the same, except the SNP loci and their estimated additive and dominance effects were used rather than the true QTL and true effects. Accordingly, the locus genotypic values G_{jk} were calculated as $G_{jk} = x_{A_k}\hat{a}_j + x_{D_k}\hat{d}_j$, where $\hat{d}_j = \frac{-b}{N} + \hat{d}_j^*$. The terms x_{A_k} and x_{D_k} were respectively the scaled additive and scaled dominance genotype dosages at the ith individual's j^{th} SNP marker. All subsequent steps were the same as in calculation of true breeding value. This method to calculate and estimate breeding value does not require assuming HWE, because the observed population genotype frequencies are used rather than HWE genotype frequencies.

In the Two-Pool Breeding Value scenarios, it should be noted that each pool was considered a separate population, and allele frequencies from the pool an individual belonged to were used to calculate or estimate breeding value. In the Two-Pool Breeding Value + GCA and Two-Pool Doubled Haploid Breeding Value + GCA scenarios, the inter-pool individuals were always used as the training set. Breeding value was predicted with the RRBLUP_D model, and GCA was predicted with the RRBLUP_GCA model.

With selection on phenotypic value, the individual's raw phenotypic value was considered an estimator of breeding value.

Expected mean cross performance

Expected mean cross performance was calculated using true QTL and their true effects if true values were used, or it was estimated using SNP markers and their estimated biological effects resulting from the RRBLUP_D model if genomic estimated values were used. The basic procedure was that the gamete probabilities were calculated for all parent genotypes *i*, then the expected progeny genotype distribution was calculated for all possible biparental crosses at each locus *j* (of either true QTL or SNPs). We assumed that gametes pair independently, and the probability of obtaining a given gamete follows a binomial distribution. In the case of autopolyploids, these assumptions are consistent with strict bivalent pairing of chromosomes in meiosis, which is the assumption used in this study. The expected progeny genotype distribution was weighted by the appropriate additive and dominance effects respectively, then all effects were summed over all *j* loci to obtain the expected mean cross performance. The detailed procedure follows.

First, the probability X_{ijl} of obtaining a given gamete *l* from each individual *i* at locus *j* with genotype x_{ij} and ploidy level ϕ was assumed to follow a binomial distribution such that

$$\mathrm{f}(k, \phi, p) = \Pr\,(X_{ijl} = k_{ij}) = \binom{\phi}{k} p^k (1-p)^{\phi - k}$$

and the binomial coefficient was

$$\binom{\phi}{k} = \frac{\phi!}{k!\,(\phi - k)!}$$

The total number of gametes sampled per meiosis was $k = \frac{\phi}{2}$. The probability *p* of obtaining gamete *l* from individual *i* at locus *j* for genotype x_i is given below.

**Genotype (x_i)**	**Gamete (*l*)**	***p***
ploidy = 2		
0		

		0	1
		1	0
	1		
		0	½
		1	½
	2		
		0	0
		1	1
ploidy = 4			
	0		
		0	1
		1	0
		2	0
	1		
		0	½
		1	½
		2	0
	2		
		0	1/6
		1	1/3
		2	1/6
	3		
		0	0
		1	½
		2	½
	4		
		0	0
		1	0
		2	1
ploidy = 6			
	0		
		0	1
		1	0
		2	0
		3	0
	1		
		0	½
		1	½
		2	0
		3	0
	2		
		0	1/5
		1	3/5
		2	1/5
		3	0
	3		
		0	1/20
		1	9/20
		2	9/20
		3	1/20
	4		

	0	0/20
	1	1/5
	2	3/5
	3	1/5
5		
	0	0
	1	0
	2	½
	3	½
6		
	0	0
	1	0
	2	0
	3	1

For a given individual i, locus j, and genotype x_i the vector $\mathbf{g}_{ij}$ contained the $0\ldots\frac{\phi}{2}$ gamete probabilities X_{ijl}. The genotype probabilities of a given biparental cross of parents i_1 and i_2 at a given locus, $\mathbf{\Gamma}_{i_1 i_2 j}$, were assumed to be the product of all possible gamete probabilities of the parents.

Third, the expected mean cross performance $C_{i_1 i_2}$ of a given biparental cross was the product of the expected genotype frequency and the genotype value at the locus, summed over all j loci. The genotype value was the sum of the scaled additive genotype dosages multiplied by the locus additive effect and the scaled dominance genotype dosages multiplied by the locus dominance effect.

$$C_{i_1 i_2} = \sum_{1}^{j} \mathbf{\Gamma}_{i_1 i_2 j} * \left[x_{A_{i_1 i_2 j}} * a_j + x_{D_{i_1 i_2 j}} * d_j \right]$$

The script used to calculate expected mean cross performance is located at https://github.com/gaynorr/QuantGenResources/blob/main/CalcCrossMeans.cpp.

Two-Pool GCA and SCA

True GCA is the average true performance of an intra-pool individual in all of its possible inter-pool hybrid combinations. To calculate the true GCA, the true expected mean cross performances of all possible inter-pool crosses were calculated using true QTL and true allelic effects as described above. Then, the true GCA ($TGCA_i$) of a given individual i was the average true expected mean cross performance of all inter-pool crosses in which the individual was a parent ($\overline{IPC_i}$) minus the average true expected mean cross performance of all possible inter-pool crosses ($\overline{IPC}_{all}$), so ($TGCA_i = \overline{IPC}_i - \overline{IPC}_{all}$.

The true SCA of a given inter-pool cross ($TSCA_{i_1 i_2}$) was then the true expected mean cross performance of the inter-pool cross ($IPC_{i_1 i_2}$) minus the average true expected mean cross performance of all possible inter-pool crosses ($\overline{IPC}_{all}$) minus the sum of the true GCA of its parents ($\overline{IPC}_{i_1}$ + $\overline{IPC}_{i_2}$), so $TSCA_{i_1 i_2} = IPC_{i_1 i_2} - \overline{IPC}_{all} - (\overline{IPC}_{i_1} + \overline{IPC}_{i_2})$.

Genomic-estimated GCA (GCA_{M_i} or GCA_{P_i}) was the sum across SNPs of the respective maternal or paternal average effects from the RRBLUP_GCA model, so $GCA_{M_i} = \sum_{ij}^{n} x_{M_{ij}} \hat{a}_{M_j}$ and $GCA_{P_i} = \sum_{ij}^{n} x_{P_{ij}} \hat{a}_{P_j}$. No genomic estimates of SCA were performed.

Phenotypic-estimated GCA of a given individual *i* was calculated in the same way as true GCA, except only the phenotypic values of the phenotyped inter-pool crosses were used.
