# Supplementary material for: Clonal diploid and autopolyploid breeding strategies to harness heterosis: insights from stochastic simulation
Source: Theor Appl Genet. 2023 Jun 8;136(7):147. doi: 10.1007/s00122-023-04377-z (PMC10250475; doi:10.1007/s00122-023-04377-z)
Supplement: Supplementary file 5 — Supplementary file5 (PDF 804 kb) [file 122_2023_4377_MOESM5_ESM.pdf]

Clonal diploid and autopolyploid breeding strategies to harness heterosis: insights from stochastic simulation. Theoretical and Applied Genetics. Marlee R. Labroo, Jeffrey B. Endelman, Dorcus C. Gemenet, Christian R. Werner, R. Chris Gaynor, Giovanny Eduardo Covarrubias-Pazaran (Excellence in Breeding Platform, Consultative Group of International Agricultural Research; covaruberpaz@gmail.com)

Supplemental Discussion

Alternatives to Two-Pool GCA for diploids

Transitioning to Two-Pool GCA can represent a significant logistical challenge for breeding programs, and species may have radically different biological constraints or costs among strategies than those assumed in our study. We explored two other methods to exploit dominance, One-Pool Cross Performance and Two-Pool Breeding Value, which may be useful to programs for which Two-Pool GCA is not feasible. With PS at high intensity, Two-Pool Breeding Value tended to outperform Two-Pool GCA at high intensity with fast or slow multiplication, with Two-Pool GCA only providing benefits at highly positive H_0 after 50 years. However, Two-Pool Breeding Value usually underperformed One-Pool Breeding Value. As such, Two-Pool Breeding Value may be a helpful transition for phenotypic programs using One-Pool Breeding Value which intend to move to use of both GS and Two-Pool GCA.

With use of GS at high intensity, Two-Pool Breeding Value provided similar gain as Two-Pool GCA after 15 years and was the second-best strategy after 50 years, with slightly reduced gain compared to Two-Pool GCA. However, there is not an apparent logistical advantage to use of Two-Pool Breeding Value with genomic prediction, because programs can choose to predict either GCA or breeding value from identical breeding programs, and GCA is likely the better or not-worse choice. As simulated, Two-Pool Breeding Value had relatively low accuracy with GS but not PS as H_0 increased, and we note that the low accuracy likely resulted from our choice to predict intra-pool genotypes from a training set of inter-pool genotypes (Moghaddar et al., 2014; Hidalgo et al., 2016). We chose not to explore this further for two reasons. First, this is analogous to prediction of purebred animals from crossbreds, which has been extensively studied (Wei & Van der Werf, 1994; Moghaddar et al., 2014; Hidalgo et al., 2016). Second, although we assumed that intra- and inter-pool phenotyping is achieved with equal thoroughness and accuracy, in practice two-pool plant breeding programs typically would not phenotype intra-pool material for all traits or in as many environments. It is unlikely that increasing intra-pool phenotyping is cost-effective, because the inter-pool genotypes must be thoroughly phenotyped for use as products anyway and this information doubles for use in population improvement. As such, further optimization of Two-Pool Breeding Value with GS was not pursued.

One-Pool Cross Performance is another alternative to Two-Pool GCA. As in previous studies, we observed that at high intensity genomic estimated One-Pool Cross Performance typically outperformed One-Pool Breeding Value with adequate H_0 (Werner et al., 2020). However, One-Pool Cross Performance underperformed two-pool strategies unless H_0 was relatively low. As such, One-Pool Cross Performance is a safer option than One-Pool Breeding Value over most H_0 values and likely benefits programs where creating two pools is infeasible. The underperformance of One-Pool Cross Performance to two-pool strategies was small after 15 years, making it an attractive short-term option, but One-Pool Cross Performance was not a substitute for two-pool strategies after 50 years in populations with heterosis.

Somewhat interestingly, both One-Pool Cross Performance and Two-Pool Breeding Value lost competitiveness with use of true values. Compared to genomic estimates, One-Pool Cross Performance lost and Two-Pool Breeding Value gained comparative advantage in accuracy over other values. Use of genomic estimates induced more inbreeding than true values, so Two-Pool Breeding Value lost advantage even as it gained accuracy, because the overall decrease in genetic drift led the two pools to diverge less. Concordantly, we observed that panmictic heterosis decreased in Two-Pool Breeding Value with use of

true values compared to genomic estimated values. One-Pool Cross Performance likely lost advantage partly due to the relatively increased accuracy of all other strategies, but perhaps also because with true values it had similar dominance gain but decreased additive gain compared to One-Pool Breeding Value. With genomic-estimated values, One-Pool Cross Performance had increased dominance value compared to One-Pool Breeding Value, likely because it more effectively prevented homozygosity due to inbreeding.

Assumptions, limitations, and future directions

We did not optimize accuracy within strategies. For example, testcrossing is necessary with phenotypic Two-Pool GCA but is suboptimal for genomic estimated Two-Pool GCA (Fristche-Neto et al., 2017; Seye et al., 2020). We did not optimize tester choice or number and simply used two random testers. With GS and Two-Pool Breeding Value, prediction of intra-pool genotypes from an inter-pool training set was suboptimal compared to use of intra-pool training genotypes, which has been demonstrated in prediction of purebred animals from crossbreds (Wei & Van der Werf, 1994; Moghaddar et al., 2014; Hidalgo et al., 2016). We assumed a fixed number of individuals in the training set, which implied that training individuals could be from the most recent one, two, or three cycles depending on strategy; this was likely a reasonable approach, but formal optimization of the training set could differentially affect strategy accuracy (Akdemir et al., 2015; Sabadin et al., 2022).

Tangentially, the accuracy of autopolyploid genomic estimates tended to be similar to diploids at low H_0, but increasingly lower than diploids at high H_0, suggesting that allelic effects may be harder to predict in autopolyploids than diploids as dominance increases with a fixed number of genotyped SNPs (Supplemental Figure 24—26). This is sensible because more dominance effects are present in autopolyploids per phenotypic observation. However, it did not seem to be the main cause of the decreased advantage of Two-Pool GCA in the autopolyploids, which also appeared with use of true values.

With use of maximum avoidance of inbreeding at high vs. low intensity, there were necessarily more full siblings per family at high vs. low intensity. Availability of additional full siblings at high intensity may have increased the accuracy of prediction of dominance values (Misztal et al., 1998), which could affect the relative performance of Two-Pool GCA. However, the difference in relative performance between Two-Pool GCA and other strategies at high vs. low intensity was also apparent with use of true values at perfect accuracy, indicating the influence of the inbreeding rate.

The relative costs we assumed may differ among applied programs. Particularly, the cost of two-pool vs. one-pool breeding depends strongly on crop biology. We assumed that the cost of controlled inter-pool crossing and multiplication was negligible, which may not be the case in some crops.

Although we completely disregarded product development strategies or prediction of inter-pool crosses in addition to GCA for reciprocal recurrent genomic selection, we presume that population improvement strategies which produce populations with higher means and similar distributions will lead to extraction of higher-value products with all else, such as product evaluation strategy, equal. Allocation of resources among stages was not explored.

We did not fully explore all possible genetic architectures, particularly those including epistasis or higher-order autopolyploid dominance. We note that positive directional dominance could arise from selection and was not necessarily present in the starting population for situations when Two-Pool GCA to presented advantages over one-pool strategies—e.g., with an initial mean dominance degree of zero and non-zero variance of dominance degrees (Falconer & Mackay, 1996; Varona et al., 2018). We did not

consider environment or genotype x environment effects, which may affect the relative performance of GS and PS and depletion of genetic variance. We assumed a fixed marker density and genome size.

Multiple frameworks to model dominance in polyploids are available; here, only digenic dominance is considered, while other frameworks allow for additional intra-locus interactions (Gallais, 2003). It does not seem likely that other valuations of various possible heterozygotes or inclusion of additional intra-locus interactions would change the relative performances of the strategies presented here, because the superfluity of Two-Pool GCA seems to arise from the increased frequency of heterozygotes in autopolyploids rather than their valuation. However, further study may reveal unexpected results.

We assessed H_0 as a predictor of various responses. H_0 appeared to explain the variance of responses among strategies well, but it is possible that its components—mean dominance degree, the variance of the dominance degrees, and the square root of the number of QTL—could reveal different patterns of strategy performance if used as predictors rather than H_0. We plotted genetic gain of the core strategies with use of true values after 50 years with use of each component as a predictor of responses with both other components held constant in all possible combinations (Supplemental Fig. 30—38). In general, we observed similar patterns as with use of H_0 for mean dominance degree and the square root of the number of QTL, with the relative performance of Two-Pool GCA increasing as each of these increased. The relative performance of Two-Pool GCA increased as mean dominance degree increased regardless of whether incomplete dominance, complete dominance, or overdominance was simulated; notably, overdominance did not decrease the relative advantage of Two-Pool GCA (Rembe et al., 2019). However, for the variance of dominance degrees, if the mean dominance degree was low then advantage of Two-Pool GCA increased as the variance of dominance degrees increased, even though the variance of dominance degrees has an inverse relationship with H_0. This seemed to be because selection on GCA led to directional dominance in the breeding population when loci with positive dominance degrees were present. This trend reversed to expectation as mean dominance degree and the number of QTL increased.
