# Supplementary material for: Clonal diploid and autopolyploid breeding strategies to harness heterosis: insights from stochastic simulation
Source: Theor Appl Genet. 2023 Jun 8;136(7):147. doi: 10.1007/s00122-023-04377-z (PMC10250475; doi:10.1007/s00122-023-04377-z)
Supplement: Supplementary file 6 — Supplementary file6 (PDF 1935 kb) [file 122_2023_4377_MOESM6_ESM.pdf]

Clonal diploid and autopolyploid breeding strategies to harness heterosis: insights from stochastic simulation. Theoretical and Applied Genetics. Marlee R. Labroo, Jeffrey B. Endelman, Dorcus C. Gemenet, Christian R. Werner, R. Chris Gaynor, Giovanny Eduardo Covarrubias-Pazaran (Excellence in Breeding Platform, Consultative Group of International Agricultural Research; covaruberpaz@gmail.com)

Supplemental Table 1. Program sizes for given strategies, estimation methods, and selection intensities.

**True, Low Intensity**	One-Pool Breeding Value	One-Pool Predicted Cross Performance	Two-Pool Breeding Value	Two-Pool GCA	Two-Pool Breeding Value + GCA	Two-Pool Doubled Haploid GCA	Two-Pool Doubled Haploid Breeding Value + GCA
Number of Pools	1	1	2	2	2	2	2
Number of Intrapool Parents per Pool, *r*	200	200	100	100	100	100	100
Number of Intrapool Crosses per Pool, *x*	100	100	50	50	50	50	40
Number of Progeny per Intrapool Cross, *y*	13	13	4	5	5	3	4
Total Number of Intrapool Progeny, *z*	1300	1300	400	500	500	300	320
Total Number of Interpool Parents, *v*	0	0	400	500	400	300	240
Total Number of Interpool Crosses	0	0	800	1000	800	600	480
Number of Progeny per Interpool Cross	0	0	1	1	1	1	1
Total Number of Interpool Progeny, *w*	0	0	800	1000	800	600	480
Total Number of Evaluation Plots	1300	1300	1200	1000	1300	600	800
Total Number of Doubled Haploid Plots	0	0	0	0	0	900	960
Total Number of Genotyping Plots	0	0	0	0	0	0	0
Total Cost (Plots)	1300	1300	1200	1000	1300	1500	1760

**True, High Intensity**	One-Pool Breeding Value	One-Pool Predicted Cross Performance	Two-Pool Breeding Value	Two-Pool GCA	Two-Pool Breeding Value + GCA	Two-Pool Doubled Haploid GCA	Two-Pool Doubled Haploid Breeding Value + GCA
Number of Pools	1	1	2	2	2	2	2
Number of Intrapool Parents per Pool, *r*	40	40	20	20	20	20	20
Number of Intrapool Crosses per Pool, *x*	20	20	10	10	10	10	10
Number of Progeny per Intrapool Cross, *y*	65	65	20	25	25	15	16
Total Number of Intrapool Progeny, *z*	1300	1300	400	500	500	300	320
Total Number of Interpool Parents, *v*	0	0	400	500	400	300	240
Total Number of Interpool Crosses	0	0	800	1000	800	600	480
Number of Progeny per Interpool Cross	0	0	1	1	1	1	1
Total Number of Interpool Progeny, *w*	0	0	800	1000	800	600	480
Total Number of Evaluation Plots	1300	1300	1200	1000	1300	600	800
Total Number of Doubled Haploid Plots	0	0	0	0	0	900	960
Total Number of Genotyping Plots	0	0	0	0	0	0	0
Total Cost (plot equivalents)	1300	1300	1200	1000	1300	1500	1760

**Genomic Estimated, Low Intensity**	One-Pool Breeding Value	One-Pool Predicted Cross Performance	Two-Pool Breeding Value	Two-Pool GCA	Two-Pool Breeding Value + GCA	Two-Pool Doubled Haploid GCA	Two-Pool Doubled Haploid Breeding Value + GCA
Number of Pools	1	1	2	2	2	2	2
Number of Intrapool Parents per Pool, *r*	200	200	100	100	100	100	100
Number of Intrapool Crosses per Pool, *x*	100	100	50	50	50	50	40
Number of Progeny per Intrapool Cross, *y*	13	13	4	5	5	3	4
Total Number of Intrapool Progeny, *z*	1300	1300	400	500	500	300	320
Total Number of Interpool Parents, *v*	0	0	400	500	400	300	240
Total Number of Interpool Crosses	0	0	800	1000	800	600	480
Number of Progeny per Interpool Cross	0	0	1	1	1	1	1
Total Number of Interpool Progeny, *w*	0	0	800	1000	800	600	480
Total Number of Evaluation Plots	1300	1300	1200	1000	1300	600	800
Total Number of Doubled Haploid Plots	0	0	0	0	0	900	960
Total Number of Genotyping Plots	1300	1300	1200	1500	1300	900	800
Total Cost (plot equivalents)	2600	2600	2400	2500	2600	2400	2560

**Genomic Estimated, High Intensity**	One-Pool Breeding Value	One-Pool Predicted Cross Performance	Two-Pool Breeding Value	Two-Pool GCA	Two-Pool Breeding Value + GCA	Two-Pool Doubled Haploid GCA	Two-Pool Doubled Haploid Breeding Value + GCA
Number of Pools	1	1	2	2	2	2	2
Number of Intrapool Parents per Pool, *r*	40	40	20	20	20	20	20
Number of Intrapool Crosses per Pool, *x*	20	20	10	10	10	10	10
Number of Progeny per Intrapool Cross, *y*	65	65	20	25	25	15	16
Total Number of Intrapool Progeny, *z*	1300	1300	400	500	500	300	320
Total Number of Interpool Parents, *v*	0	0	400	500	400	300	240
Total Number of Interpool Crosses	0	0	800	1000	800	600	480
Number of Progeny per Interpool Cross	0	0	1	1	1	1	1
Total Number of Interpool Progeny, *w*	0	0	800	1000	800	600	480
Total Number of Evaluation Plots	1300	1300	1200	1000	1300	600	800
Total Number of Doubled Haploid Plots	0	0	0	0	0	900	960
Total Number of Genotyping Plots	1300	1300	1200	1500	1300	900	800
Total Cost (plot equivalents)	2600	2600	2400	2500	2600	2400	2560

**Phenotypic, Low Intensity**	One-Pool Phenotypic Value	One-Pool Cross Performance	Two-Pool Phenotypic Value	Two-Pool GCA	Two-Pool Phenotypic Value + GCA	Two-Pool Doubled Haploid GCA	Two-Pool Doubled Haploid Phenotypic Value + GCA
Number of Pools	1	1	2	2	2	2	2
Number of Intrapool Parents per Pool, *r*	200	200	100	100	100	100	100
Number of Intrapool Crosses per Pool, *x*	100	100	50	50	50	50	50
Number of Progeny per Intrapool Cross, *y*	26	26	9	13	13	5	5
Total Number of Intrapool Progeny, *z*	2600	2600	900	1300	1300	500	500
Total Number of Interpool Parents, *v*	0	0	900	1300	600	500	300
Total Number of Interpool Crosses	0	0	1800	2600	1200	1000	600
Number of Progeny per Interpool Cross	0	0	1	1	1	1	1
Total Number of Interpool Progeny, *w*	0	0	1800	2600	1200	1000	600
Total Number of Evaluation Plots	2600	2600	2700	2600	2500	1000	1100
Total Number of Doubled Haploid Plots	0	0	0	0	0	1500	1500
Total Number of Genotyping Plots	0	0	0	0	0	0	0
Total Cost (plot equivalents)	2600	2600	2700	2600	2500	2500	2600

**Phenotypic, High Intensity**	One-Pool Phenotypic Value	Two-Pool Phenotypic Value	Two-Pool GCA	Two-Pool Phenotypic Value + GCA	Two-Pool Doubled Haploid GCA	Two-Pool Doubled Haploid Phenotypic Value + GCA
Number of Pools	1	2	2	2	2	2
Number of Intrapool Parents per Pool, *r*	40	40	20	20	20	20
Number of Intrapool Crosses per Pool, *x*	20	10	10	10	10	10
Number of Progeny per Intrapool Cross, *y*	130	42	63	50	25	23
Total Number of Intrapool Progeny, *z*	2600	840	1260	1000	500	460
Total Number of Interpool Parents, *v*	0	840	1260	760	500	360
Total Number of Interpool Crosses	0	1680	2520	1520	1000	720
Number of Progeny per Interpool Cross	0	1	1	1	1	1
Total Number of Interpool Progeny, *w*	0	1680	2520	1520	1000	720
Total Number of Evaluation Plots	2600	2520	2520	2520	1000	1180
Total Number of Doubled Haploid Plots	0	0	0	0	1500	1380
Total Number of Genotyping Plots	0	0	0	0	0	0
Total Cost (plot equivalents)	2600	2520	2520	2520	2500	2560

Supplemental Table 2. Full breeding strategy descriptions for various scenarios. Variable values are given in Supplemental Table 1.

	Genomic Estimated Values and (True Values)	Phenotypic Values, Fast Multiplication	Phenotypic Values, Slow Multiplication
One-Pool Breeding Value	1. Make x crosses with y progeny per cross among the r selected parents.<br>**2. Multiply and genotype the z intra-pool progeny from 1. Predict breeding value using RRBLUP_D on 2,000 most recently evaluated intra-pool individuals (or calculate), select 2 best individuals per family (cross), and recycle to 1.**<br>3. Phenotype multiplied z intra-pool progeny from 2.	1. Make x crosses with y progeny per cross among the r selected parents.<br>2. Multiply the z intra-pool progeny from 1.<br>**3. Phenotype multiplied z intra-pool progeny from 2. Select 2 best individuals per family (cross) on phenotype and recycle to 1.**	1. Make x crosses with y progeny per cross among the r selected parents.<br>2. Multiply the z intra-pool progeny from 1.<br>3. Multiply the z intra-pool progeny from 2.<br>**4. Phenotype multiplied z intra-pool progeny from 3. Select 2 best individuals per family (cross) on phenotype and recycle to 1.**
One-Pool Cross Performance	1. Make the x selected crosses with y progeny per cross.<br>**2. Multiply and genotype the z intrapool progeny from 1. Predict cross performance using RRBLUP_D on 2,000 most recently evaluated intra-pool individuals (or calculate), select the 2 best crosses within sets of families that satisfy maximum avoidance of inbreeding, and recycle to 1.**<br>3. Phenotype multiplied z intra-pool progeny from 2.		
Two-Pool Breeding Value	1. Within each pool, make x crosses with y progeny per cross among the r selected parents.	1. Within each pool, make x crosses with y progeny	1. Within each pool, make x crosses with y progeny per

	**2. Multiply and genotype the z intra-pool progeny from 1. Predict breeding value using RRBLUP_D on 2,000 most recently evaluated inter-pool individuals (or calculate), select 2 best individuals per family within pools, and recycle to 1. For the inter-pool crossing block, select two random individuals per pool. Cross the z intra-pool progeny per pool to both individuals selected from the opposing pool, with 1 progeny per cross, totaling w inter-pool progeny.**<br>3. Phenotype multiplied z intra-pool seedling progeny from 2. Multiply and genotype w inter-pool progeny from 2.<br>4. Phenotype multiplied w inter-pool progeny from 3.	per cross among the r selected parents.<br>2. Multiply the z intra-pool progeny from 1. For the inter-pool crossing block, select two random individuals per pool. Cross the z intra-pool progeny per pool to both individuals selected from the opposing pool, with 1 progeny per cross, totaling w inter-pool progeny.<br>**3. Phenotype multiplied z intra-pool progeny from 2. Select 2 best individuals per family (cross) on phenotype and recycle to 1. Multiply the w inter-pool progeny from 2.**<br>4. Phenotype the multiplied w inter-pool progeny from 2.	cross among the r selected parents.<br>2. Multiply the z intra-pool progeny from 1.<br>3. Multiply the z intra-pool progeny from 2.<br>**4. Phenotype multiplied z intra-pool progeny from 3. Select 2 best individuals per family (cross) on phenotype and recycle to 1. For the inter-pool crossing block, select two random individuals per pool. Cross the z intra-pool progeny per pool to both individuals selected from the opposing pool, with 1 progeny per cross, totaling w inter-pool progeny.**<br>5. Multiply the w inter-pool progeny from 4.<br>6. Multiply the w inter-pool progeny from 5.<br>7. Phenotype the multiplied w inter-pool progeny from 6.
Two-Pool GCA	1. Within each pool, make x crosses with y progeny per cross among the r selected parents.<br>**2. Genotype the z intra-pool progeny from 1. Predict GCA using RRBLUP_GCA on 2,000 most recently evaluated inter-pool individuals (or calculate),**	1. Within each pool, make x crosses with y progeny per cross among the r selected parents.<br>2. For the inter-pool crossing block, select two random testers per pool. Cross the z intra-pool	1. Within each pool, make x crosses with y progeny per cross among the r selected parents.<br>2. Multiply the z intra-pool progeny from 1 to induce flowering

	**select 2 best individuals per family within pools, and recycle to 1. For the inter-pool crossing block, select two random testers per pool. Cross the z intra-pool progeny per pool to both testers from the opposing pool, with 1 progeny per cross, totaling w inter-pool progeny.**<br>3. Multiply and genotype the w inter-pool progeny from 2.<br>4. Phenotype the multiplied w inter-pool progeny from 3.	progeny per pool to both testers from the opposing pool, with 1 progeny per cross, totaling w inter-pool progeny.<br>3. Multiply the w inter-pool progeny from 2.<br>**4. Phenotype multiplied inter-pool progeny from 3. Select within pools on GCA and recycle to 1.**	3. Multiply the z intra-pool progeny from 2 to induce flowering.<br>4. For the inter-pool crossing block, select two random testers per pool. Cross the z intra-pool progeny per pool to both testers from the opposing pool, with 1 progeny per cross, totaling w inter-pool progeny.<br>5. Multiply the w inter-pool progeny from 4.<br>6. Multiply the w inter-pool progeny from 5.<br>**7. Phenotype multiplied w inter-pool progeny from 6. Select within pools on GCA and recycle to 1.**
Two-Pool Doubled Haploid GCA	1. Within each pool, make x crosses with y progeny per cross among the r selected parents.<br>**2. Make 1 doubled haploid per z intra-pool progeny and genotype. Predict GCA using RRBLUP_GCA on 2,000 most recently evaluated inter-pool individuals (or calculate), select 2 best individuals per family within pools, and recycle to 1.**<br>3. For the inter-pool crossing block, select two random testers per pool. Cross the z intra-pool	1. Within each pool, make x crosses with y progeny per cross among r selected parents.<br>2. Make 1 doubled haploid per z intra-pool progeny.<br>3. For the inter-pool crossing block, select two random testers per pool. Cross the z intra-pool progeny per pool to both testers from the opposing pool, with 1 progeny per cross, totaling w inter-pool progeny.	1. Within each pool, make x crosses with y progeny per cross among r selected parents.<br>2. Make 1 doubled haploid per z intra-pool progeny.<br>3. Multiply z doubled haploids from 2 to induce flowering.<br>4. Multiply z doubled haploids from 3 to induce flowering.<br>5. For the inter-pool crossing block, select two random testers per pool. Cross the z intra-pool progeny per pool to both testers from the opposing pool, with 1

	progeny per pool to both testers from the opposing pool, with 1 progeny per cross, totaling *w* inter-pool progeny.<br>4. Multiply and the genotype the *w* inter-pool progeny from 3.<br>5. Phenotype the multiplied *w* inter-pool progeny from 4.	4. Multiply *w* inter-pool progeny from 3.<br>**5. Phenotype multiplied *w* inter-pool progeny from 4. Select within pools on GCA and recycle to 1.**	progeny per cross, totaling *w* inter-pool progeny.<br>6. Multiply *w* inter-pool progeny from 5.<br>7. Multiply *w* inter-pool progeny from 6.<br>**8. Phenotype multiplied inter-pool progeny from 7. Select within pools on GCA and recycle to 1.**
Two-Pool Breeding Value + GCA	1. Within each pool, make *x* crosses with *y* progeny per cross among the *r* selected parents.<br>**2. Multiply and genotype *z* intra-pool progeny from 1. Predict GCA using RRBLUP_GCA on 2,000 most recently evaluated inter-pool individuals (or calculate), select 2 best individuals per family within pools, and recycle to 1. Predict breeding value using RRBLUP_D on 2,000 most recently evaluated inter-pool individuals (or calculate) and advance the top ~75% of individuals per family per pool to the inter-pool crossing block. Of the *v* individuals advanced per pool, select two random testers.**<br>3. Phenotype the multiplied *z* intra-pool progeny from 2. For the	1. Within each pool, make *x* crosses with *y* progeny per cross among the *r* selected parents.<br>2. Multiply the *z* intra-pool progeny from 1. For the inter-pool crossing block, select two random testers per pool. Cross the *z* intra-pool progeny per pool to both testers from the opposing pool, with 1 progeny per cross.<br>3. Phenotype the *z* multiplied intra-pool progeny from 2. Multiply the inter-pool progeny from 2. After phenotyping completes, select the top 75% of intra-pool individuals per family per pool on phenotype, and cull inter-	1. Within each pool, make *x* crosses with *y* progeny per cross among the *r* selected parents.<br>2. Multiply the *z* intra-pool progeny from 1.<br>3. Multiply the *z* intra-pool progeny from 2.<br>4. Phenotype the multiplied *z* intra-pool progeny from 3. For the inter-pool crossing block, select two random testers per pool. Cross the *z* intra-pool progeny per pool to both testers from the opposing pool, with 1 progeny per cross. After crossing and phenotyping complete, select the top 75% of intra-pool individuals per family per pool on phenotype, and cull inter-pool crosses which do not result from these *v*

	inter-pool crossing block, cross the *v* advanced intra-pool progeny per pool to both testers from the opposing pool, with 1 progeny per cross, totaling *w* inter-pool progeny.<br>4. Multiply and genotype the *w* inter-pool progeny from 3.<br>5. Phenotype the *w* inter-pool progeny from 4.	pool crosses which do not result from these *v* individuals. This results in *w* inter-pool progeny.<br>**4. Phenotype the *w* inter-pool progeny from 3. Select within pools on GCA and recycle to 1.**	individuals. This results in *w* inter-pool progeny.<br>5. Multiply the *w* inter-pool progeny from 4.<br>6. Multiply the *w* inter-pool progeny from 5.<br>**7. Phenotype inter-pool progeny from 6. Select within pools on GCA and recycle to 1.**
Two-Pool Doubled Haploid Breeding Value + GCA	1. Within each pool, make *x* crosses with *y* progeny per cross among the *r* selected parents.<br>**2. Make 1 doubled haploid per *z* intra-pool progeny and genotype. Predict GCA using RRBLUP_GCA on 2,000 most recently evaluated inter-pool individuals (or calculate), select 2 best individuals per family within pools, and recycle to 1. Predict breeding value using RRBLUP_D on 2,000 most recently evaluated inter-pool individuals (or calculate) and advance the top ~75% of individuals per family per pool to the inter-pool crossing block. Of the *v* individuals advanced per pool, select two random testers.**<br>3. Multiply the *z* doubled haploids from 2. For the inter-pool	1. Within each pool, make *x* crosses with *y* progeny per cross among the *r* selected parents.<br>2. Make 1 doubled haploid per *z* intra-pool progeny.<br>3. Multiply the *z* doubled haploids. For the inter-pool crossing block, select two random testers per pool. Cross the *z* intra-pool progeny per pool to both testers from the opposing pool, with 1 progeny per cross.<br>4. Phenotype the *z* intra-pool doubled haploids. Multiply inter-pool progeny from 3. After phenotyping completes, select the top 75% of intra-pool individuals per family per pool on	1. Within each pool, make *x* crosses with *y* progeny per cross among the *r* parents.<br>2. Make 1 doubled haploid per *z* intra-pool progeny.<br>3. Multiply the *z* doubled haploids from 2.<br>4. Multiply doubled haploids from 3.<br>5. Phenotype the *z* intra-pool doubled haploids. For the inter-pool crossing block, select two random testers per pool. Cross the *z* intra-pool progeny per pool to both testers from the opposing pool, with 1 progeny per cross. After phenotyping completes, select the top 75% of intra-pool individuals per family per pool on phenotype, and cull inter-pool crosses which do not result from these *v*

crossing block, cross the *v* advanced intra-pool progeny per pool to both testers from the opposing pool, with 1 progeny per cross, totaling *w* inter-pool progeny.

4. Phenotype the multiplied *z* doubled haploids from 3. Multiply and genotype the *w* inter-pool progeny from 3.
5. Phenotype the *w* inter-pool progeny from 4.

phenotype, and cull inter-pool crosses which do not result from these *v* individuals. This results in *w* inter-pool progeny.

5. **Phenotype the *w* inter-pool progeny from 4. Select within pools on GCA and recycle to 1.**

individuals. This results in *w* inter-pool progeny.

6. Multiply the *w* inter-pool progeny from 5.
7. Multiply the *w* inter-pool progeny from 6.
8. **Phenotype the *w* inter-pool progeny from 7. Select within pools on GCA and recycle to 1.**

Supplemental Table 3. Genotype frequencies for diploids, autotetraploids, and autohexaploids at Hardy-Weinberg equilibrium if allele frequencies $p = q = 0.5$. As ploidy increases, the frequency of homozygous genotypes decreases.

Diploid Genotype	Diploid Genotype Frequency*	Autotetraploid Genotype	Autotetraploid Genotype Frequency*	Autohexaploid Genotype	Autohexaploid Genotype Frequency*
0	0.25	0	0.06	0	0.02
				1	0.09
		1	0.25	2	0.23
1	0.5	2	0.38	3	0.31
		3	0.25	4	0.23
				5	0.09
2	0.25	4	0.06	6	0.02

*With $p = q = 0.5$ at Hardy-Weinberg equilibrium

Supplemental Table 4. Inbreeding depression values for simulated autotetraploid populations and their corresponding diploid populations produced by genome reduction. The code to generate the populations is available at https://github.com/gaynorr/ClonalHybridStrategies.

Replicate	HWE Mean, 4x	Fully Inbred Mean, 4x	HWE Mean, 2x	Fully Inbred Mean, 2x	Inbreeding Depression, 4x	Inbreeding Depression, 2x
1	10.88	0	7.26	0	-10.88	-7.26
2	10.04	0	6.69	0	-10.04	-6.69
3	11.61	0	7.74	0	-11.61	-7.74
4	12.45	0	8.3	0	-12.45	-8.3
5	11.94	0	7.96	0	-11.94	-7.96
6	12.2	0	8.13	0	-12.2	-8.13
7	10.97	0	7.31	0	-10.97	-7.31
8	11.38	0	7.59	0	-11.38	-7.59
9	11.89	0	7.92	0	-11.89	-7.92
10	11.57	0	7.71	0	-11.57	-7.71
